# Pyrazolyl-s-triazine with indole motif as a novel of epidermal growth factor receptor/cyclin-dependent kinase 2 dual inhibitors

**DOI:** 10.3389/fchem.2022.1078163

**Published:** 2022-11-25

**Authors:** Ihab Shawish, Mohamed S. Nafie, Assem Barakat, Ali Aldalbahi, Hessa H. Al-Rasheed, M. Ali, Walhan Alshaer, Mazhar Al Zoubi, Samha Al Ayoubi, Beatriz G. De la Torre, Fernando Albericio, Ayman El-Faham

**Affiliations:** ^1^ Department of Math and Sciences, College of Humanities and Sciences, Prince Sultan University, Riyadh, Saudi Arabia; ^2^ Department of Chemistry, College of Science, King Saud University, Riyadh, Saudi Arabia; ^3^ Department of Chemistry, Faculty of Science, Suez Canal University, Ismaïlia, Egypt; ^4^ Cell Therapy Center, The University of Jordan, Amman, Jordan; ^5^ Department of Basic Medical Sciences, Faculty of Sciences, Yarmouk University, Irbid, Jordan; ^6^ KwaZulu-Natal Research Innovation and Sequencing Platform (KRISP) School of Laboratory Medicine and Medical Sciences, College of Health Sciences, University of KwaZulu-Natal, Durban, South Africa; ^7^ Peptide Science Laboratory, School of Chemistry and Physics, University of KwaZulu-Natal, Durban, South Africa; ^8^ CIBER-BBN (Networking Centre on Bioengineering, Biomaterials and Nanomedicine) and Department of Organic Chemistry, University of Barcelona, Barcelona, Spain; ^9^ Chemistry Department, Faculty of Science, Alexandria University, Alexandria, Egypt

**Keywords:** pyrazolyl-s-triazine, indole, anticancer profile, EGFR/CDK-2, apoptosis

## Abstract

A series of pyrazolyl-*s*-triazine compounds with an indole motif was designed, synthesized, and evaluated for anticancer activity targeting dual EGFR and CDK-2 inhibitors. The compounds were tested for cytotoxicity using the MTT assay. Compounds **3h**, **3i**, and **3j** showed promising cytotoxic activity against two cancer cell lines, namely A549, MCF-7, and HDFs (non-cancerous human dermal fibroblasts). Compound **3j** was the most active candidate against A549, with an IC_50_ of 2.32 ± 0.21 μM. Compounds **3h** and **3i** were found to be the most active hybrids against MCF-7 and HDFs, with an IC_50_ of 2.66 ± 0.26 μM and 3.78 ± 0.55 μM, respectively. Interestingly, **3i** showed potent EGFR inhibition, with an IC_50_ of 34.1 nM compared to Erlotinib (IC_50_ = 67.3 nM). At 10 μM, this candidate caused 93.6% and 91.4% of EGFR and CDK-2 inhibition, respectively. Furthermore, **3i** enhanced total lung cancer cell apoptosis 71.6-fold (43.7% compared to 0.61% for the control). Given the potent cytotoxicity exerted by **3i** through apoptosis-mediated activity, this compound emerges as a promising target-oriented anticancer agent.

## 1 Introduction

The discovery of effective drugs for the treatment of various types of cancer continues to be a major challenge for the research community (Abbot et al., 2017). In this regard, despite its serious side effects, chemotherapy is one of the most widely used therapeutic strategies to limit the spread of cancer cells. Accordingly, efforts are still ongoing to develop chemotherapeutic drugs that selectively target cancer cells, thereby reducing the side effects of the treatment. In this context, compounds with a *s*-triazine moiety have shown potent activity against a wide spectrum of cancer cells (Liu, 2015). Indeed, such compounds have been reported to exert antimicrobial (Kumar et al., 2014), anti-inflammatory (Marín-Ocampo et al., 2019; Asadi et al., 2021), antimalarial (Adhikari et al., 2020), antifungal and antibacterial (Bhat et al., 2019), antiviral (Viira et al., 2016), and antioxidant activity. However, the greatest attention has been devoted to the anticancer activity of *s*-triazine derivatives. In this regard, their antitumor activity targets a wide range of cancer cell lines, and they are therefore ideal candidates on which to base the development of more effective drugs for the treatment of cancer, including leukemia, breast cancer, colon cancer, cervical cancer, and many others (Liu, 2015; Viira et al., 2016; Cascioferro et al., 2017; Dugan and Pollyea, 2018; Yan et al., 2018; Bhat et al., 2019; Lolak et al., 2020; Hu et al., 2021; Mehmood et al., 2021; Reddy et al., 2021; Sharma et al., 2021; Tayyab Imtiaz et al., 2021).

**FIGURE 1:**
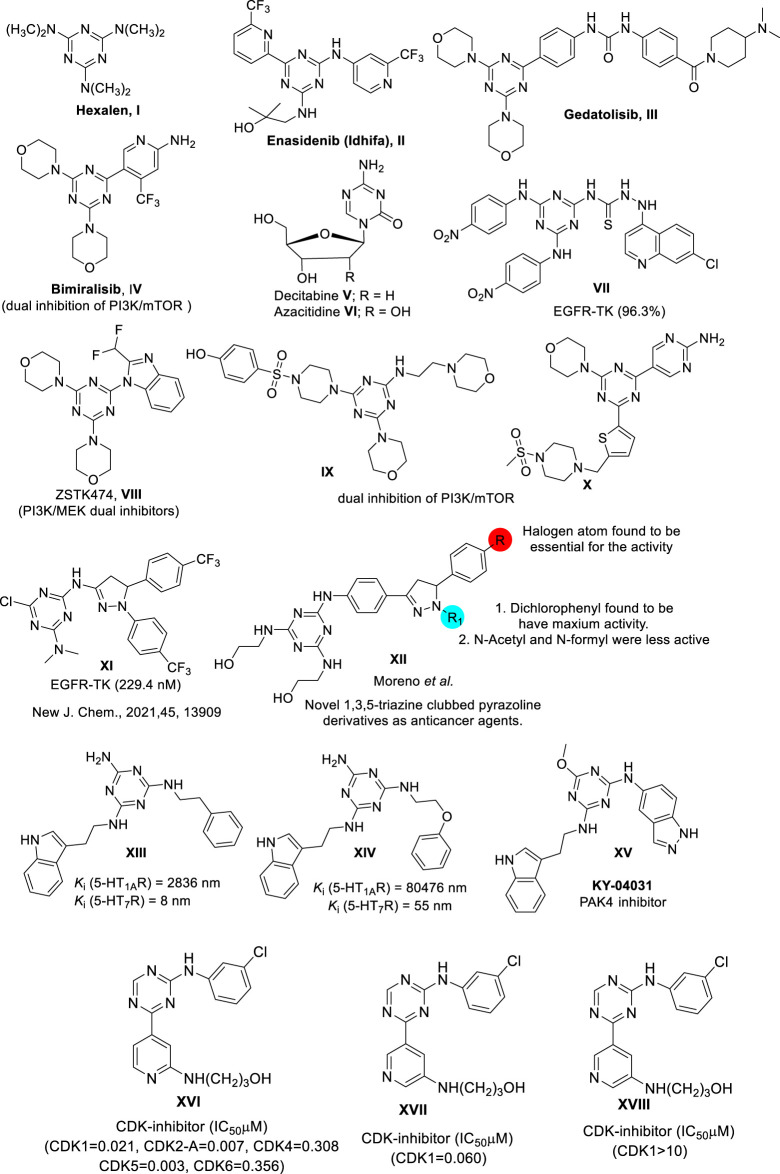
Selected *s*-triazine derivatives as an anticancer agent exhibiting a range of mechanisms of target.

The examples given below are representative *s*-triazine-based drugs approved by the U.S. FDA (Figure 1). Hexalen (**I**), also referred to as Altretamine (Figure 1), is an antineoplastic drug used for the treatment of refractory ovarian cancer (Keldsen et al., 2003). Approved by the U.S. FDA in 2017, Enasidenib (Idhifa) (**II**) is used for the treatment of IDH2-positive acute leukemia (Kim, 2017). Another example of this class of *s-*triazine-based anticancer drugs is Gedatolisib (**III**), which inhibits the PI3K/mTOR signaling cascade and is used to treat breast cancer (Shor et al., 2022). Bimiralisib (PQR309) (**IV**) is a novel brain-penetrant dual inhibitor of PI3K-mTOR that shows potent selectivity compared to other PI3K lipid kinases (Zoncu et al., 2011; Beaufils et al., 2017; Wang and Tortorella, 2022). Recently, the antineoplastic drugs Decitabine (**V**) (Guan et al., 2015) and Azacitidine (**VI**) (Salim et al., 2016) have been used to target acute myeloid leukemia and chronic myelomonocytic leukemia, respectively.

The following lead molecules also bear the *s*-triazine moiety and show biological activity of interest: **VII**(Rahim et al., 2018); **VIII**(Van Dort et al., 2015); **IX**; and **X** (Zhang et al., 2020; Hu et al., 2021). These compounds have shown remarkable *in vitro,* and *in vivo* capacity to inhibit Epidermal Growth Factor Receptor EGFR-TK, PI3K/MEK, and PI3K/mTOR, respectively. Compound **XI** designed based on *s*-triazine nucleus tethered pyrazole motif targeted epidermal growth factor receptor-tyrosine kinase (EGFR-TK) inhibitors (Raghu et al., 2021). **XII** is an example of novel s-triazine clubbed 2-pyrazoline derivative, and it has shown potent inhibitory activity against several tumor cell lines, including lung, leukemia, colon, melanoma, ovary, CNS, renal, breast, and prostate cancers (Moreno et al., 2018). Lead compound **XIII** (KY-04031) shows a combination of indole and indazole pharmacophores-based s-triazine core, and it is the first-in-class anticancer drug targeting p21-activated kinase 4 (PAK4) (Ryu et al., 2014). Compounds **XIV** and **XV** are other interesting molecules. They represent a new series of indoleaminotriazine core fused to an alkyl aryl moiety, and they show high affinity (Ki < 60 nM) and selectivity as 5-HT_7_ receptors (Kułaga et al., 2020). In this regard, 5-HT_7_ receptor antagonists are attractive therapeutic targets for cancer and CNS disorders. Moreover, these receptors more specifically targeted against prostate cancer (Cinar et al., 2021), triple-negative breast cancer (Gautam et al., 2016), and lung cancer (Du et al., 2020). On the other hand, overexpression of cyclin-dependent kinase 2 (CDK-2) is a major factor causing abnormal regulation of the cell cycle, which is directly associated with excessive proliferation in cancer cells (Lapenna and Giordano, 2009). Consequently, the development of novel CDK-2 inhibitors has drawn the attention of the research community because of the expected activity of these compounds against a wide range of cancer cell lines (Davies et al., 2002; Knockaert et al., 2002; Oudah et al., 2019). In this regard, the conjugated compounds **XVI**, **XVII**, and **XVIII** are examples of *s*-triazine analogs that have shown significant inhibitory activity against CDK-2 and therefore significant activity against a wide spectrum of cancer cell lines (Kumar et al., 2019).

**FIGURE 2:**
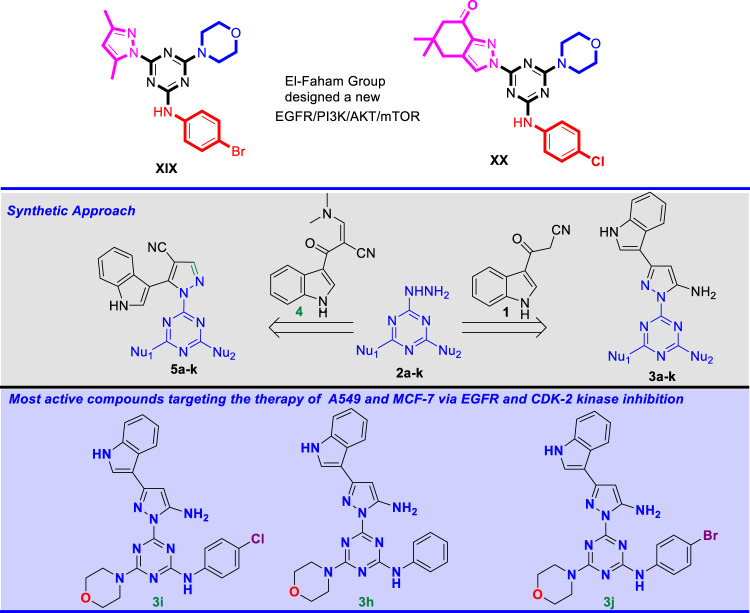
Novel pyrazolyl-s-triazine derivatives synthesized by our group targeting the EGFR/PI3K/AKT/mTOR cascade and CDK-2 inhibition.

Based on this finding, our research group recently developed a new hybrid of the 1,3,5-triazine core structure, **XIX** (Shawish et al., 2022b) and **XX** (Shawish et al., 2022a) (Figure 2) targeting the EGFR/PI3K/AKT/mTOR cascades. Here we report a novel series of compounds bearing three distinct pharmacophores, namely pyrazole, 1,3,5-triazine, and indole motifs. The anticancer activity of these conjugated compounds was assessed against the two cancer cell lines A549 (non-small cell lung cancer), MCF-7 (breast cancer), and HDFs (non-cancerous human dermal fibroblasts). EGFR and CDK-2 were also explored as molecular targets.

## 2 Materials and methods

### 2.1 Chemistry

#### 2.1.1 Material and methods

Full general notes about material and methods are provided in the SI.

##### 2.1.1.1 Synthesis of 3-(1*H*-indol-3-yl)-3-oxopropanenitrile, 1

Cyanoacetyl indole derivative **1** was prepared by cyanoacetylation of indole following the synthetic pathway reported by Bergman and co-workers (Bergman et al., 2004); yield (91%); m. p. 241 °C; ^1^H-NMR (DMSO-*d*_6_): δ 4.49 (s, 2H, CH_2_), 7.22-7.25 (m, 2H, Ar-H), 7.49 (d, 1H, Ar-H), 8.13 (d, 1H, Ar-H), 8.36 (s, 1H, Ar-H), 12.18 (s, 1H, NH); ^13^C-NMR (DMSO-*d*_6_): δ 182.9, 136.7, 135.5, 125.2, 123.4, 123.2, 121.2, 116.5, 114.5, 112.4, 29.4.

##### 2.1.1.2 Synthesis of *mono*-hydrazinyl-*s*-triazine derivatives 2a-k

**2a-k** were synthesized following the method reported in (Farooq et al., 2019; Shawish et al., 2022a; Shawish et al., 2022b).

##### 2.1.1.3 Synthesis of (Z)-3-(dimethylamino)-2-(1*H*-indole-3-carbonyl)acrylonitrile, 4

Enaminonitrile-indole derivative **4** was prepared by M. Radwan and coworkers, starting with cyanoacetyl indole derivative **1** (Radwan and El-Sherbiny, 2007). m. p. 162 °C (Lit [45]161-163°C); ^1^H NMR (400 MHz, DMSO-*d*_6_, ppm) *δ*: 3.26 (s, 3H, NCH_3_), 3.35 (s, 3H, NCH_3_), 7.12–7.20 (m, 2H, Ar-H), 7.45 (d, 1H, *J* = 7.6 Hz, Ar-H), 8.00 (s, 1H, C=CHN), 8.14 (d, 1H, *J* = 7.2 Hz, Ar-H), 8.28 (s, 1H, Ar-H), 11.76 (br s, 1H, NH). ^13^C NMR (100 MHz, DMSO-*d*_6_, ppm) *δ*: 47.4 (N-CH3), 77.6 (C-CN), 111.9, 114.7 (CN), 121.1, 121.8, 122.5, 126.8, 131.2, 135.8, 158.7 (C-N (Me)_2_), 181.7 (CO).

##### 2.1.1.4 General procedure for the synthesis of pyrazolyl-amine-*s*-triazine derivatives 3a-k from 3-(1*H*-indol-3-yl)-3-oxopropanenitrile 1 (GP1)

3-(1*H*-Indol-3-yl)-3-oxopropanenitrile **1** (1 equiv.) was suspended in ethanol with stirring at room temperature for a few minutes. Hydrazinyl-*s*-triazine derivative (1 equiv.) and *p*-toluenesulfonic acid (1 equiv.) were then added to this suspension, following the method reported by I. Ahmad and coworkers (Ahmad et al., 2012). The solution was then allowed to reflux (12-18 h) with frequent monitoring of its progress by TLC (ethyl acetate/*n*-hexane 8:2 or MeOH/CHCl_3_, 1:9). The solution became dark yellow immediately after adding *p*-toluenesulfonic acid and it turned clear during the first hour of the reflux. After the reaction was complete, depending on the TLC analysis, which showed the target products as bright blue spots in all cases, the mixture was cooled to room temperature and treated with 10% Na_2_CO_3_ (w/w) solution to afford the crude product as an off-white to yellow precipitate, which was subsequently filtered, washed with water several times, and dried. Column chromatography (ethyl acetate/*n*-hexane 6:4) was used to purify some of the prepared pyrazolyl-amine-*s*-triazine derivatives, achieving a yield of 70-85%.

##### 2.1.1.5 1-(4,6-Di (piperidin-1-yl)-1,3,5-triazin-2-yl)-3-(1*H*-indol-3-yl)-1*H*-pyrazol-5-amine, 3a

3-(Cyanoacetyl)indole of **1** and dipiperidine triazine hydrazine **2a** were used in the general procedure (GP1) to obtain the target pyrazolyl-amin-*s*-triazine derivative **3a** as an off-white solid in 86% yield, without the need for further purification. m. p. 288-290°C; ^1^H-NMR (DMSO-*d*_6_): *δ* 11.27 (s, 1H, NH), 8.27 (d, 1H, *J* = 7.2 Hz, C=CHN), 7.75 (s, 1H, Ar-H), 7.38 (d, 1H, *J* = 8.0 Hz, Ar-H), 7.07 (m, 2H, Ar-H), 6.69 (s, 2H, NH_2_), 5.74 (s, 1H, C-H, pyrazole), 3.76 (s, 8H, 4CH_2_N-), 1.64 (s, 4H, 2CH_2_-), 1.54 (s, 8H, 4CH_2_-); ^13^C-NMR (DMSO-*d*_6_): *δ* 164.1, 163.7 (C=N, Triaz.), 151.1 (C-pyraz.), 149.9, 146.2, 138.3, 137.1, 128.8, 128.50, 126.0, 125.7, 109.3, 85.5 (C-pyraz.), 44.5 (CH_2_N-), 25.9 (CH_2_), 24.8 (CH_2_). Anal. Calc. for C_24_H_29_N_9_ (443.56); C, 64.99; H, 6.59; N, 28.42. Found C, 65.89; H, 6.55; N, 28.36; HRMS-ESI (m/z) calculated for [M + H]^+^ 444.56; found: 444.4621.

##### 2.1.1.5 1-(4,6-Dimorpholino-1,3,5-triazin-2-yl)-3-(1*H*-indol-3-yl)-*1H*-pyrazol-5-amine, 3b

4,4'-(6-Hydrazinyl-1,3,5-triazine-2,4-diyl)dimorpholine **2b** and 3-(cyanoacetyl)indole of **1** were used in the general procedure (GP1) to obtain the desired product **3b** as an off-white precipitate in 89% yield; m. p. 188-189°C; ^1^H NMR (400 MHz, DMSO-*d*_6_) *δ* 11.32 (d, *J* = 2.6 Hz, 1H, NH), 8.34 – 8.23 (m, 1H, C=CHN), 7.77 (d, *J* = 2.7 Hz, 1H, Ar-H), 7.41 (d, *J* = 7.9 Hz, 1H, Ar-H), 7.11 (dt, *J* = 18.8, 7.0 Hz, 2H,Ar-H), 6.70 (s, 2H, NH_2_), 5.78 (s, 1H, C-H, pyrazole), 3.93 – 3.71 (m, 8H, 4CH_2_O-), 3.68 (t, *J* = 4.6 Hz, 8H, 4CH_2_N-); ^13^C NMR (101 MHz, DMSO-*d*_6_) *δ* 165.0, 163.6 (C=N, Triaz.), 151.3 (C-pyraz.), 150.3, 137.1, 125.7, 125.4, 122.1, 122.1, 120.2, 112.1, 109.7, 86.1 (C-pyraz.), 66.5 (2CH_2_O), 44.1 (2CH_2_N). Anal. Calc. for C_22_H_25_N_9_O_2_ (447.50); C, 59.05; H, 5.63; N, 28.17. Found C, 59.12; H, 5.75; N, 28.09; HRMS-ESI (m/z) calculated for [M + H]^+^ 448.50; found: 448.4691.

##### 2.1.1.6 3-(1*H*-Indol-3-yl)-1-(4-morpholino-6-(piperidin-1-yl)-1,3,5-triazin-2-yl)-*1H*-pyrazol-5-amine, 3c

3-(cyanoacetyl)indole of **1** and 3-(*1H*-indol-3-yl)-1-(4-morpholino-6-(piperidin-1-yl)-1,3,5-triazin-2-yl)-*1H*-pyrazol-5-amine **2c** were used in the general procedure (GP1) to obtain the target product **3c** as an off-white solid in 89% yield; m. p. 178-179°C; ^1^H NMR (400 MHz, DMSO-*d*_6_) *δ* 11.32 (s, 1H, NH), 8.35 – 8.23 (m, 1H, C=CHN), 7.76 (s, 1H, Ar-H), 7.41 (d, *J* = 8.1 Hz, 1H, Ar-H), 7.11 (dt, *J* = 21.7, 7.2 Hz, 2H, H-C_5_, H-C_6_), 6.72 (s, 2H, NH_2_), 5.77 (s, 1H, C-H, pyrazole), 3.76 (s, 4H, 2CH_2_O), 3.46 (m, 8H, 4CH_2_N-), 1.71 – 1.59 (m, 2H, -CH_2_), 1.55 (s, 4H, 2-CH_2_); ^13^C NMR (101 MHz, DMSO-*d*_6_) *δ* 165.1, 164.5, 163.7 (3C = N, Triaz.), 151.2(C-pyraz.), 150.1, 137.1, 125.7, 125.4, 125.3, 122.1, 120.1, 112.2, 109.7, 86.2 (C-pyraz.), 66.5 (2CH_2_O), 44.5, 44.1 (2CH_2_N), 26.0 (2CH_2_), 24.8 (2CH_2_). Anal. Calc. for C_23_H_27_N_9_O (445.53); C, 62.01; H, 6.11; N, 28.30. Found C, 61.91; H, 6.01; N, 28.29; HRMS-ESI (m/z) calculated for [M + H]^+^ 446.53; found: 446.4965.

##### 2.1.1.7 4-(5-Amino-3-(1*H*-indol-3-yl)-1*H*-pyrazol-1-yl)-*N*-phenyl-6-(piperidin-1-yl)-1,3,5- triazin-2-amine, 3d

3-(Cyanoacetyl)indole **1** and 4-hydrazinyl-*N*-phenyl-6-(piperidin-1-yl)-1,3,5-triazin-2-amine **2d** were used in the general procedure (GP1) to obtain the target compound **3d** as a pale yellow solid in 91% yield; m. p. 266-268°C; ^1^H NMR (500 MHz, DMSO-*d*_6_) *δ* 11.40 (s, 1H, NH), 9.76 (s, 1H, NH), 8.25 (s, 1H, C=CHN), 7.82 (s, 1H), 7.73 (s, 1H), 7.60 – 7.49 (m, 1H), 7.47 – 7.40 (m, 1H), 7.33 (d, *J* = 7.8 Hz, 2H), 7.12 (dt, *J* = 19.3, 7.4 Hz, 3H), 7.02 (t, *J* = 7.3 Hz, 2H, NH_2_), 5.81 (s, 1H, C-H, pyraz.), 3.80 (s, 4H, 2CH_2_N), 1.62 (q, *J* = 5.9 Hz, 2H, -CH_2_), 1.55 (q, *J* = 5.6 Hz, 4H, 2CH_2_-); ^13^C NMR (126 MHz, DMSO-*d*_6_) *δ* 164.5, 163.0 (C=N, Triaz.), 152.3 (C-pyraz), 150.1, 146.1, 140.2, 138.3, 137.2, 129.2 (2C, C-Ar), 128.7, 126.2, 126.1, 125.6, 123.0, 122.3, 120.3, 112.3, 86.3 (C-pyarz.), 44.8 (2CH_2_N), 26.0, 24.7 (2CH_2_-), 21.3 (2CH_2_). Anal. Calc. for C_25_H_25_N_9_ (451.54); C, 66.50; H, 5.58; N, 27.92. Found C, 66.39; H, 5.45; N, 27.82; HRMS-ESI (m/z) calculated for [M + H]^+^ 452.54; found: 452.6198.

##### 2.1.1.8 4-(5-Amino-3-(1*H*-indol-3-yl)-*1H*-pyrazol-1-yl)-*N*-(4-chlorophenyl)-6-(piperidin-1-yl)-1,3,5-triazin-2-amine, 3e

3-(cyanoacetyl)indole of **1** and *N*-(4-chlorophenyl)-4-hydrazinyl-6-(piperidin-1-yl)-1,3,5-triazin-2-amine **2e** were used in the general procedure (GP1) to prepare the desired pyrazolyl-amine-*s*-triazine derivative **3e** as an off-white solid in 93% yield, without the need for further purification; m. p. 295-296°C; ^1^H NMR (500 MHz, DMSO-*d*_6_) *δ* 11.31 (s, 1H, NH), 9.85 (s, 1H, NH), 8.27 (s, 1H, C=CHN), 7.76 (s, 2H), 7.57 – 7.43 (m, 1H), 7.43 – 7.34 (m, 3H), 7.09 (td, *J* = 15.6, 7.5 Hz, 2H), 6.86 (s, 2H, NH_2_), 5.74 (s, 1H, C-H, pyraz.), 3.78 (s, 4H, 2CH_2_N), 1.62 (q, *J* = 6.0 Hz, 2H, CH_2_-), 1.54 (d, *J* = 8.2 Hz, 4H, 2CH_2_-); ^13^C NMR (126 MHz, DMSO-*d*_6_) *δ* 164.5, 163.4 (C=N, Triaz.), 151.4 (C-pyraz.), 150.6, 146.1, 139.4, 138.3, 137.2, 128.9 (2C-Ar), 128.7, 126.4, 126.1, 125.6, 122.2, 119.9, 112.2, 109.7, 85.8 (C-pyraz.), 44.8 (2CH_2_N), 25.9, 24.7 (2CH_2_-), 21.3 (CH_2_-). Anal. Calc. for C_25_H_24_ClN_9_ (485.98); C, 61.79; H, 4.98; N, 25.94. Found C, 61.82; H, 5.09; N, 25.77; HRMS-ESI (m/z) calculated for [M + H]^+^ 486.98; found: 486.8796.

##### 2.1.1.9 4-(5-Amino-3-(*1H*-indol-3-yl)-*1H*-pyrazol-1-yl)-*N*-(4-bromophenyl)-6-(piperidin-1-yl)-1,3,5-triazin-2-amine, 3f

3-(Cyanoacetyl)indole **1** and *N*-(4-bromophenyl)-4-hydrazinyl-6-(piperidin-1-yl)-1,3,5-triazin-2-amine **2f** were used in the general procedure (GP1) to obtain the target **3f** an off-white precipitate in 96% yield; m. p. 271-274°C; ^1^H NMR (500 MHz, DMSO-*d*_6_) *δ* 11.31 (s, 1H, NH), 9.85 (s, 1H, NH), 8.27 (s, 1H, C=CHN), 7.76 (s, 2H), 7.50 (s, 2H), 7.40 (d, *J* = 8.0 Hz, 2H), 7.10 (h, *J* = 7.8, 7.4 Hz, 3H), 6.85 (s, 2H, NH_2_), 5.74 (s, 1H, C-H, pyraz.), 3.78 (s, 4H, 2CH_2_N), 1.62 (q, *J* = 6.0 Hz, 2H, CH_2_-), 1.57 – 1.37 (m, 4H, 2CH_2_-); ^13^C NMR (126 MHz, DMSO-*d*_6_) *δ* 164.5, 163.4 (C=N, Triaz.), 151.4 (C-pyraz.), 150.6, 146.1, 139.8, 138.3, 137.2, 131.8, 128.7, 126.1, 125.7, 125.6, 122.2, 119.9, 114.3, 112.2, 109.7, 85.8 (C-pyraz.), 44.8 (2CH_2_N), 25.9, 24.7 (2CH_2_-), 21.3 (CH_2_-). Anal. Calc. for C_25_H_24_BrN_9_ (530.43); C, 56.61; H, 4.56; N, 23.77. Found C, 56.59; H, 4.48; N, 23.87; HRMS-ESI (m/z) calculated for [M + H]^+^ 531.43; found: 531.3310.

##### 2.1.1.10 4-(5-Amino-3-(*1H*-indol-3-yl)-*1H*-pyrazol-1-yl)-*N*-(4-methoxyphenyl)-6-(piperidin-1-yl)-1,3,5-triazin-2-amine, 3g

The general procedure (GP3.1) was applied to afford the target product as pale-yellow precipitate, 81% yield, m. p. 288-290°C; ^1^H NMR (500 MHz, DMSO-*d*_6_) *δ* 11.32 (s, 1H, NH), 9.86 (s, 1H, NH), 8.27 (s, 1H, C=CHN), 7.76 (s, 2H, Ar-H), 7.55 – 7.47 (m, 1H), 7.43 – 7.35 (m, 3H, Ar-H), 7.10 (td, J = 14.9, 7.1 Hz, 2H, Ar-H), 6.86 (s, 2H, NH_2_), 5.74 (s, 1H, C_b_-H), 3.78 (s, 3H, OCH_3_), 3.37 (s, 4H, 2CH_2_N), 1.61 (q, J = 6.0, 5.5 Hz, 2H), 1.54 (d, J = 7.9 Hz, 4H); ^13^C NMR (126 MHz, DMSO-*d*_6_) δ 164.5, 163.4 (C=N, Triaz.), 151.4 (C-pyraz.), 150.5, 146.1, 139.4, 138.3, 137.2 (C1), 128.9, 128.7, 126.4, 126.1, 125.6, 122.2, 119.9, 112.2, 109.7, 85.9 (C-pyraz.), 79.7, 44.8 (OCH_3_), 31.2 (2CH_2_N), 25.9, 24.7, 21.3. Anal. Calc. for C_26_H_27_N_9_O (481.56); C, 64.85; H, 5.65; N, 26.18. Found C, 64.92; H, 5.55; N, 26.28; HRMS-ESI (m/z) calculated for [M + H]^+^ 482.56; found: 482.4896.

##### 2.1.1.11 4-(5-Amino-3-(1*H*-indol-3-yl)-*1H*-pyrazol-1-yl)-6-morpholino-*N*-phenyl-1,3,5-triazin-2-amine, 3h

3-(Cyanoacetyl)indole of **1** and 4-hydrazinyl-6-morpholino-*N*-phenyl-1,3,5-triazin-2-amine **2h** were used in the general procedure (GP1) to obtain the desired pyrazolyl-amine-*s*-triazine derivative **3h** as an off-white solid, which was purified by column chromatography (ethyl acetate/*n*-hexane 6:4) and collected in 82% yield; m. p. 262-264°C; ^1^H NMR (500 MHz, DMSO-*d*_6_) *δ* 11.33 (s, 1H, NH), 9.82 (s, 1H, NH), 8.27 (s, 1H, C=CHN), 8.02 (d, *J* = 27.6 Hz, 1H), 7.77 (s, 2H), 7.47 – 7.24 (m, 3H), 7.17-6.99 (m, 3H), 6.78 (m, 2H, NH_2_), 5.75 (s, 1H, C-H, pyraz.), 3.80 (s, 4H, 2CH_2_O), 3.73 – 3.64 (m, 4H, 2CH_2_N); ^13^C NMR (126 MHz, DMSO-*d*_6_) *δ* 165.2, 163.4 (C=N, Triaz.), 151.5 (C-pyraz), 150.6, 140.2, 137.2, 129.2 (3Ar-C), 125.6, 123.0, 122.1, 120.5, 120.1 (3Ar-C), 112.1, 109.7, 85.8 (C-pyraz), 66.5 (2CH_2_O), 44.2 (2CH_2_N). Anal. Calc. for C_24_H_23_N_9_O (453.51); C, 63.56; H, 5.11; N, 27.80. Found C, 63.50; H, 5.07; N, 27.88; HRMS-ESI (m/z) calculated for [M + H]^+^ 454.51; found: 454.6009.

##### 2.1.1.12 4-(5-Amino-3-(1*H*-indol-3-yl)-*1H*-pyrazol-1-yl)-*N*-(4-chlorophenyl)-6-morpholino-1,3,5-triazin-2-amine, 3i

3-(Cyanoacetyl)indole of **3i** and *N*-(4-chlorophenyl)-4-hydrazinyl-6-morpholino-1,3,5-triazin-2-amine **2i** were used in the general procedure (GP1) to obtain the target pyrazolyl-amine-*s*-triazine product **3i** as pure off-white solid in 93% yield; m. p. 256-258°C; ^1^H NMR (500 MHz, DMSO-*d*_6_) *δ* 11.33 (s, 1H, NH), 9.95 (s, 1H, NH), 8.55 – 8.21 (m, 1H, C=CHN), 8.10 (s, 1H), 7.77 (s, 2H), 7.38 (t, *J* = 9.2 Hz, 3H), 7.25 – 7.03 (m, 2H), 6.82 (d, *J* = 104.1 Hz, 2H, NH_2_), 5.74 (s, 1H, C-pyraz.), 3.79 (s, 4H, 2CH_2_O), 3.68 (t, *J* = 4.8 Hz, 4H, 2CH_2_N); ^13^C NMR (126 MHz, DMSO-*d*_6_) *δ* 165.2, 163.4 (C=N, Triaz.), 151.5 (C-pyraz), 150.7, 139.2, 137.2, 129.0 (3Ar-C), 126.6, 125.6, 122.2 (4Ar-C), 120.0, 112.2, 109.7, 85.9 (C-pyraz.), 66.4 (2CH_2_O), 44.3 (2CH_2_N). Anal. Calc. for C_24_H_22_ClN_9_O (487.95); C, 59.08; H, 4.54; N, 25.84. Found C, 59.13; H, 4.63; N, 25.78; HRMS-ESI (m/z) calculated for [M + H]^+^ 488.95; found: 489.0012.

##### 2.1.1.13 4-(5-Amino-3-(*1H*-indol-3-yl)-*1H*-pyrazol-1-yl)-*N*-(4-bromophenyl)-6-morpholino-1,3,5-triazin-2-amine, 3j

*N-*(4-Bromophenyl)-4-hydrazinyl-6-morpholino-1,3,5-triazin-2-amine **2j** and 3-(cyanoacetyl)indole of **one** were used in the general procedure (GP1) to obtain the target pyrazolyl-amine-*s*-triazine product **3j** as an off-white solid in 92% yield and without further purification; m. p. 268-270°C; ^1^H NMR (500 MHz, DMSO-*d*_6_) δ 11.32 (s, 1H, NH), 9.93 (s, 1H, NH), 8.54 – 8.22 (m, 1H, C=CHN), 8.02 (d, *J* = 22.5 Hz, 1H), 7.72 (d, *J* = 42.7 Hz, 2H), 7.50 (d, *J* = 8.4 Hz, 2H), 7.39 (d, *J* = 8.2 Hz, 1H), 7.20 – 7.06 (m, 2H), 6.99 – 6.55 (m, 2H, NH_2_), 5.74 (s, 1H, C-H, pyraz.), 3.78 (s, 4H, 2CH_2_O), 3.68 (t, *J* = 4.8 Hz, 4H, 2CH_2_N); ^13^C NMR (126 MHz, DMSO-*d*_6_) *δ* 165.1, 163.3 (C=N, Triaz.), 151.5 (C-pyraz), 150.7, 139.6, 137.2, 131.9 (3Ar-C), 125.6, 123.2, 122.2 (2Ar-C), 120.0, 114.5, 112.2, 109.7, 85.9 (C-pyraz), 66.4 (2CH_2_O), 44.3 (2CH_2_N). Anal. Calc. for C_24_H_22_BrN_9_O (532.41); C, 54.14; H, 4.17; N, 23.68. Found C, 54.08; H, 4.09; N, 23.74; HRMS-ESI (m/z) calculated for [M + H]^+^ 533.41; found: 533.3619.

##### 2.1.1.14 4-(5-Amino-3-(*1H*-indol-3-yl)-*1H*-pyrazol-1-yl)-*N-*(4-methoxyphenyl)-6-morpholino-1,3,5-triazin-2-amine, 3k

4-Hydrazinyl-*N-*(4-methoxyphenyl)-6-morpholino-1,3,5-triazin-2-amine **2k** and 3-(cyanoacetyl)indole of **i** were used in the general procedure (GP1) to obtain the target pyrazolyl-amine-*s*-triazine derivative **3k** as an off-white solid in 94% yield; m. p. 299-300°C; ^1^H NMR (500 MHz, DMSO-*d*_6_) *δ* 11.29 (s, 1H, NH), 9.70 (s, 1H, NH), 8.33 – 8.14 (m, 1H, C=CHN), 7.87 (s, 1H), 7.74 (s, 1H), 7.67 – 7.53 (m, 1H), 7.38 (d, *J* = 8.0 Hz, 1H), 7.18 – 7.00 (m, 2H), 6.93 (d, *J* = 14.2 Hz, 3H, 2Ar-H + NH), 6.69 (s, 1H, NH), 5.72 (s, 1H, C-H, pyraz.), 3.79 (d, *J* = 28.2 Hz, 4H, 2CH_2_O), 3.71 (s, 3H, OCH_3_), 3.69 – 3.63 (m, 4H, 2CH_2_N); ^13^C NMR (126 MHz, DMSO-*d*_6_) *δ* 165.4, 163.8, 163.3 (3C = N, Triaz.), 155.4, 151.6 (C-pyraz.), 137.1 (C1), 133.0, 128.8, 125.6, 125.4, 122.8, 122.1 (2Ar-C), 121.9, 120.0, 114.4 (2Ar-C), 112.1, 109.7, 85.8 (C-pyraz.), 66.5 (2CH_2_O), 55.7 (OCH_3_), 44.2 (2CH_2_N). Anal. Calc. for C_25_H_25_N_9_O_2_ (483.54); C, 62.10; H, 5.21; N, 26.07. Found C, 62.23; H, 5.11; N, 26.17; HRMS-ESI (m/z) calculated for [M + H]^+^ 484.54; found: 484.6033.

##### 2.1.1.15 General procedure for the synthesis of pyrazolyl-carbonitrile-*s*-triazine derivatives from enaminenitrile-indole (GP2)

3-(Dimethylamino)-2-(1*H*-indole-3-carbonyl)acrylonitrile **4** (1 equiv.) was suspended in EtOH with stirring at room temperature, followed by the addition of hydrazinyl-*s*-triazine derivative (1 equiv.). The reaction mixture was refluxed for 18-24 h. It became clear immediately with heating while a pale-yellow precipitate appeared in the first 2 h of reflux. The progress of the reaction was monitored by TLC (ethyl acetate/*n*-hexane 7:3), which showed the appearance of a blue-bright spot, representing the desired pyrazolyl-carbonitrile-*s*-triazine derivative. After the completion of the reaction, the flask was cooled, and the yellow precipitate of the product was filtered and washed with cold EtOH. In most cases, the pyrazolyl-carbonitrile-*s*-triazine derivatives **5a-k** obtained required further purification by column chromatography (ethyl acetate/*n*-hexane 1:1) to remove trace amounts of the starting material.

In the case of phenylhydrazinyl-*s*-triazine derivatives **2e-g** and **2h-k**, the application of the previous procedure resulted in a mixture of the desired product with other side products, which negatively affected the yield. To overcome this problem, a modified procedure was applied by adding p-toluene sulfonic acid (1 equiv.) to the reaction mixture (Ahmad et al., 2012). After 18-24 h of reflux, a 10% NaHCO_3_ solution was stirred with the off-white precipitate of the product, which was then filtered and washed with water several times to obtain the targeted pyrazolyl-carbonitrile-*s*-triazine derivatives **5e-g** and **5h-k** in good yield and as pure off-white solids, without the need for further purification, except for a small number of products that required column chromatography (ethyl acetate/*n*-hexane 6:4) for purification.

##### 2.1.1.16 2-(4,6-Di (piperidin-1-yl)-1,3,5-triazin-2-yl)-3-(1*H*-indol-3-yl)-2,3-dihydro-1*H*-pyrazole-4-carbonitrile, 5a

3-(Dimethylamino)-2-(1H-indole-3-carbonyl)acrylonitrile **4** and hydrazine-*s*-triazine derivative **2a** were used in the general procedure (GP2) to obtain the crude pyrazolyl-carbonitrile-*s*-triazine derivative **5a**, which was then purified by column chromatography (ethyl acetate/*n*-hexane 4:6) and collected as a yellow solid in 88% yield; m. p. 262-264°C; ^1^H NMR (400 MHz, DMSO-*d*_6_) *δ* 11.70 (d, *J* = 2.9 Hz, 1H, NH), 8.33 (s, 1H, C=CHN), 7.79 (d, *J* = 2.8 Hz, 1H, H-C_a_), 7.47 (d, *J* = 8.2 Hz, 1H, Ar-H), 7.15 – 6.96 (m, 3H, Ar-H), 3.57 (t, *J* = 5.1 Hz, 4H, 2-CH_2_N-), 3.27 – 3.12 (m, 4H, 2-CH_2_N-), 1.45 (q, *J* = 6.0 Hz, 4H, 2-CH_2_-), 1.36 (s, 4H, 2-CH_2_-), 1.05 (s, 4H, 2-CH_2_-); ^13^C NMR (101 MHz, DMSO-*d*_6_) *δ* 170.9, 164.7, 163.4 (3C = N, Triaz.), 145.7 (C_pyraz_), 143.4, 136.7, 127.2 (C_pyraz_) (d, *J* = 33.5 Hz), 125.9, 122.4 (d, *J* = 30.2 Hz), 120.8, 119.0 (d, *J* = 29.3 Hz), 114.9 (CN), 112.7, 103.4, 94.5 (C_pyraz_), 44.2 (4CH_2_N-), 25.7, 24.6. Anal. Calc. for C_25_H_27_N_9_ (453.55); C, 66.20; H, 6.00; N, 27.85. Found C, 66.15; H, 5.89; N, 27.83; HRMS-ESI (m/z) calculated for [M + H]^+^ 454.55; found: 454.6107.

##### 2.1.1.17 1-(4,6-Dimorpholino-1,3,5-triazin-2-yl)-5-(1*H*-indol-3-yl)-1*H*-pyrazole-4 carbonitrile, 5b

3-(Dimethylamino)-2-(1H-indole-3-carbonyl)acrylonitrile **4** and dimorpholino-triazine hydrazine derivative **2b** were used in the general procedure (GP2) to obtain the target product as a yellow solid in 84% yield; m. p. 328-330°C; ^1^H NMR (500 MHz, DMSO-*d*_6_) *δ* 11.65 (d, *J* = 2.9 Hz, 1H, NH), 8.30 (d, *J* = 24.5 Hz, 1H, C=CHN), 7.77 (d, *J* = 2.8 Hz, 1H, H-C_pyraz_), 7.55 – 7.37 (m, 1H, Ar-H), 7.18 – 7.04 (m, 1H, Ar-H), 7.03 – 6.91 (m, 2H, Ar-H), 3.79 – 3.58 (m, 4H, 2-CH_2_O-), 3.44 (s, 4H, 2-CH_2_O-), 3.11 (d, *J* = 23.8 Hz, 8H, 4-CH_2_N-); ^13^C NMR (126 MHz, DMSO-*d*_6_) *δ* 164.9, 163.1 (C=N, Triaz.), 145.9 (C_pyraz_), 143.6, 136.6, 127.4 (C_pyraz_), 125.9, 122.5, 120.8, 119.1, 114.7, 112.7, 103.5, 94.9 (C_pyraz_), 66.4, 66.2 (4CH_2_O-), 43.7 (4CH_2_N-). Anal. Calc. for C_23_H_23_N_9_O_2_ (457.50); C, 60.38; H, 5.07; N, 27.55. Found C, 60.30; H, 5.21; N, 27.48; HRMS-ESI (m/z) calculated for [M + H]^+^ 458.50; found: 458.6100.

##### 2.1.1.18 5-(1*H*-Indol-3-yl)-1-(4-morpholino-6-(piperidin-1-yl)-1,3,5-triazin-2-yl)-1*H*-pyrazol4-carbonitrile, 5c

3-(Dimethylamino)-2-(1*H*-indole-3-carbonyl)acrylonitrile **4** and the morpholine piperidine-triazine hydrazine derivative **2c** were used in the general procedure (GP2) to obtain the target pyrazolyl-carbonitrile-*s*-triazine derivative **5c** as a yellow solid in 77% yield; m. p. 291-293°C; ^1^H NMR (500 MHz, DMSO-*d*_6_) *δ* 11.65 (d, *J* = 2.7 Hz, 1H, NH), 8.30 (s, 1H, C=CHN), 7.75 (s, 1H, H-C_pyraz_), 7.44 (s, 1H, Ar-H), 7.11 (t, *J* = 7.3 Hz, 1H, Ar-H), 6.96 (d, *J* = 7.3 Hz, 2H, Ar-H), 3.55 (s, 4H, 4H, 2-CH_2_O-), 3.10 (d, *J* = 32.5 Hz, 8H, 4-CH_2_N-), 1.42 (d, *J* = 5.6 Hz, 2H, CH_2_), 1.33 (s, 2H, CH_2_), 1.03 (s, 2H, CH_2_); ^13^C NMR (126 MHz, DMSO-*d*_6_) *δ* 165.0, 164.6, 163.2 (3C = N, Triaz.), 145.8 (C_pyraz_), 143.5, 136.6, 127.3, 125.9, 122.5, 120.7, 119.0, 114.8, 112.7, 103.4, 94.7 (C_pyraz_), 66.2 (2CH_2_O-), 44.2 (d, *J* = 10.8 Hz (4CH_2_N-), 43.7 (4CH_2_N-), 25.6 (2CH_2_), 24.5 (-CH_2_). Anal. Calc. for C_24_H_25_N_9_O (455.53); C, 63.28; H, 5.53; N, 27.67. Found C, 63.19; H, 5.45; N, 27.75; HRMS-ESI (m/z) calculated for [M + H]^+^ 456.53; found: 456.4899.

##### 2.1.1.19 5-(1*H*-Indol-3-yl)-1-(4-(phenylamino)-6-(piperidin-1-yl)-1,3,5-triazin-2-yl)-1*H*-pyrazole-4-carbonitrile, 5d

3-(Dimethylamino)-2-(1*H*-indole-3-carbonyl)acrylonitrile **4,** aniline-piperidine-*s*-triazine hydrazine derivative **2d**, and *p*-toluenesulfonic acid were used in the general procedure (GP2) to obtain the crude product **5d**, which was recrystallized from ethanol and collected as a white crystals in 72% yield; m. p. 298-299°C; ^1^H NMR (400 MHz, DMSO-*d*_6_) *δ* 11.91 (s, 1H, NH), 9.98 (s, 1H, NH), 8.33 – 8.23 (m, 3H, C=CHN +2Ar-H), 7.71 (d, *J* = 8.1 Hz, 2H, H-C_pyraz_ + Ar-H), 7.49 (d, *J* = 7.9 Hz, 1H), 7.37 (t, *J* = 7.7 Hz, 2H), 7.20 (dt, *J* = 18.7, 7.2 Hz, 2H), 7.05 (t, *J* = 7.4 Hz, 1H), 3.83 (d, *J* = 5.7 Hz, 4H, 2-CH_2_N-), 1.66 (q, *J* = 5.7 Hz, 2H, -CH_2_), 1.58 (s, 4H, 2CH_2_-); ^13^C NMR (101 MHz, DMSO-*d*_6_) *δ* 183.1, 164.6, 163.4 (3C = N, Triaz.), 154.2, 142.6 (C_pyraz_), 139.8, 136.8, 131.6, 129.4 (2Ar-C), 126.9, 123.2, 122.1 (2Ar-C), 121.7, 120.3, 116.5 (2 Ar-C), 112.5, 103.9, 79.8, 44.8 (2CH_2_N-), 25.9 (2CH_2_), 24.7 (CH_2_). Anal. Calc. for C_26_H_23_N_9_ (461.53); C, 67.66; H, 5.02; N, 27.31. Found C, 67.55; H, 5.12; N, 27.39; HRMS-ESI (m/z) calculated for [M + H]^+^ 462.53; found: 462.3393.

##### 2.1.1.20 1-(4-((4-Chlorophenyl)amino)-6-(piperidin-1-yl)-1,3,5-triazin-2-yl)-5-(1*H*-indol-3-yl)-1*H*-pyrazole-4-carbonitrile, 5e

*N-*(4-Chlorophenyl)-4-hydrazinyl-6-(piperidin-1-yl) -1,3,5- triazin-2-amine **2e,** *p*-toluenesulfonic acid, and 3-(dimethylamino)-2-(1*H*-indole-3-carbonyl)acrylonitrile **4** were used in the general procedure (GP2) to obtain the target product **5e** as a white solid in 87% yield; m. p. 308-310°C; ^1^H NMR (500 MHz, DMSO-*d*_6_) *δ* 11.85 (s, 1H, NH), 10.04 (s, 1H, NH), 8.25 – 8.18 (m, 3H, C=CHN +2Ar-H), 7.75 – 7.67 (m, 2H), 7.44 (d, *J* = 8.1 Hz, 1H), 7.42 – 7.36 (m, 2H), 7.19 – 7.08 (m, 2H), 3.88 – 3.67 (m, 4H, 2-CH_2_N-), 1.63 (q, *J* = 5.9 Hz, 2H, -CH_2_), 1.54 (s, 4H, 2-CH_2_); ^13^C NMR (126 MHz, DMSO-*d*_6_) *δ* 183.0, 163.4 (C=N, Triaz.), 154.2, 142.6 (C_pyraz_), 138.8, 136.8, 131.6 (2Ar-C), 129.2 (2Ar-C), 126.9, 123.1, 122.1, 121.7 (2Ar-C), 116.5, 112.5 (2Ar-C), 103.9, 79.7, 44.9 (2CH_2_N-), 25.9 (2CH_2_), 24.6 (CH_2_). Anal. Calc. for C_26_H_22_ClN_9_ (495.98); C, 62.96; H, 4.47; N, 25.42. Found C, 63.06; H, 4.39; N, 25.50; HRMS-ESI (m/z) calculated for [M + H]^+^ 496.98; found: 497.0091.

##### 2.1.1.21 1-(4-((4-Bromophenyl)amino)-6-(piperidin-1-yl)-1,3,5-triazin-2-yl)-5-(1*H-*indol-3-yl)-1*H*-pyrazole-4-carbonitrile, 5f

3-(Dimethylamino)-2-(1*H*-indole-3-carbonyl)acrylonitrile **4**, *N-*(4-bromophenyl)-4-hydrazinyl-6-(piperidin-1-yl)-1,3,5-triazin-2-amine **2f,** and *p*-toluenesulfonic acid were used in the general procedure (GP2) to obtain the desired pyrazolyl-*s*-triazine derivative **5f** as a yellow crystals in 67% yield; m. p. 308-310°C; ^1^H NMR (500 MHz, DMSO-*d*_6_) *δ* 11.76 (s, 1H, NH), 8.34 (d, *J* = 2.1 Hz, 1H, C=CHN), 7.82 (s, 1H), 7.62 (d, *J* = 8.4 Hz, 1H), 7.44 (d, *J* = 8.2 Hz, 2H), 7.11 (t, *J* = 7.9 Hz, 3H), 6.98 (t, *J* = 7.8 Hz, 2H), 3.63 (d, *J* = 59.9 Hz, 4H, 2-CH_2_N-), 1.46 (s, 2H, -CH_2_), 1.34 (s, 4H, 2-CH_2_); ^13^C NMR (126 MHz, DMSO-*d*_6_) *δ* 164.9 (C=N, Triaz.), 145.6 (C_pyraz_), 143.8, 136.7, 131.8, 127.6 (2Ar-C) 122.6 (2Ar-C), 122.3, 120.8 (2Ar-C), 118.8 (2Ar-C), 114.8, 112.9 (2Ar-C), 103.0, 94.3 (C_pyraz._), 44.2 (2CH_2_N-), 25.7 (2CH_2_), 24.4 (CH_2_). Anal. Calc. for C_26_H_22_BrN_9_ (540.43); C, 57.78; H, 14.79; N, 23.33. Found C, 57.81; H, 14.63; N, 23.22; HRMS-ESI (m/z) calculated for [M + H]^+^ 541.43; found: 541.3961.

##### 2.1.1.22 5-(1*H*-Indol-3-yl)-1-(4-((4-methoxyphenyl)amino)-6-(piperidin-1-yl)-1,3,5-triazin-2-yl)-1*H*-pyrazole-4-carbonitrile, 5g

4-Hydrazinyl-*N-*(4-methoxyphenyl)-6-(piperidin-1-yl)-1,3,5-triazin-2-amine **2g**, 3-(dimethylamino)-2-(1*H*-indole-3-carbonyl)acrylonitrile **4**, and *p*-toluenesulfonic acid were used in the general procedure (GP2) to obtain the desired pyrazolyl-*s*-triazine derivative **5g** as an off-white solid in 85% yield; m. p. 273-275°C; ^1^H NMR (400 MHz, DMSO-*d*_6_) *δ* 11.91 (s, 1H, NH), 9.85 (s, 1H, NH), 8.42 (s, 1H, C=CHN), 8.29 (d, *J* = 11.1 Hz, 2H, 2Ar-H), 8.22 – 8.03 (m, 1H), 7.61 (d, *J* = 8.5 Hz, 1H), 7.50 (d, *J* = 8.1 Hz, 1H), 7.20 (dt, *J* = 17.6, 7.3 Hz, 2H), 6.95 (d, *J* = 8.8 Hz, 2H), 3.80 (s, 3H, OCH_3_), 3.75 (s, 4H, 2-CH_2_N-), 1.72 – 1.61 (m, 2H, -CH_2_), 1.55 (s, 4H, 2-CH_2_); ^13^C NMR (101 MHz, DMSO-*d*_6_) *δ* 183.1, 164.8, 163.6 (3C = N, Triaz.), 155.6, 154.2, 142.4 (C_pyraz._), 136.8, 132.8, 131.6, 126.9, 123.2, 122.1, 121.9 (2Ar-C), 121.7, 116.6, 114.5 (2Ar-C), 112.5, 103.9, 79.8, 55.8 (OCH_3_), 44.8 (2CH_2_N-), 26.0 (2CH_2_), 24.7 (2CH_2_). Anal. Calc. for C_27_H_25_N_9_O (491.56); C, 65.97; H, 5.13; N, 25.65. Found C, 66.09; H, 5.20; N, 25.55; HRMS-ESI (m/z) calculated for [M + H]^+^ 492.56; found: 492.4812.

##### 2.1.1.23 5-(1*H*-Indol-3-yl)-1-(4-morpholino-6-(phenylamino)-1,3,5-triazin-2-yl)-1*H*-pyrazole-4-carbonitrile, 5h

2-(1*H*-Indole-3-carbonyl)acrylonitrile **4**, 4-hydrazinyl-6-morpholino-*N*-phenyl-1,3,5-triazin-2-amine **2h**, and *p*-toluenesulfonic acid were used in the general procedure (GP3.2) to obtain the crude product of the target derivative **5h**, which was subsequently purified by column chromatography (ethyl acetate/*n*-hexane 7:3) and collected as an off-white solid in 75% yield; m. p. 314-316°C; ^1^H NMR (400 MHz, DMSO-*d*_6_) *δ* 11.93 (s, 1H, NH), 10.07 (s, 1H, NH), 8.38 – 8.21 (m, 3H, C=CHN +2Ar-H), 7.72 (d, *J* = 8.1 Hz, 2H, H-C_pyraz._+Ar-H), 7.50 (d, *J* = 8.1 Hz, 1H), 7.37 (t, *J* = 7.7 Hz, 2H), 7.20 (dt, *J* = 17.7, 7.2 Hz, 2H), 7.06 (t, *J* = 7.5 Hz, 1H), 4.02 – 3.75 (m, 4H, 2-CH_2_O-), 3.70 (s, 4H, 2-CH_2_N-); ^13^C NMR (101 MHz, DMSO-*d*_6_) *δ* 183.1, 165.2, 163.3 (3C = N, Triaz.), 154.3, 142.8 (C_pyraz_), 139.6, 136.8, 131.0, 129.5, 126.9, 123.0, 121.6, 120.5, 116.5, 112.9, 103.9, 66.5 (2CH_2_O-), 44.3 (2CH_2_N-). Anal. Calc. for C_25_H_21_N_9_O (463.51); C, 64.78; H, 4.57; N, 27.20. Found C, 64.85; H, 4.51; N, 27.13; HRMS-ESI (m/z) calculated for [M + H]^+^ 464.51; found: 464.6622.

##### 2.1.1.24 1-(4-((4-Chlorophenyl)amino)-6-morpholino-1,3,5-triazin-2-yl)-5-(1*H*-indol-3-yl)-1*H*-pyrazole-4-carbonitrile, 5i

3-(Dimethylamino)-2-(1*H*-indole-3-carbonyl)acrylonitrile **4**, *N-*(4-chlorophenyl)-4-hydrazinyl-6-morpholino-1,3,5-triazin-2-amine **2i**, and *p*-toluenesulfonic acid were used in the general procedure (GP2) to obtain the target derivative **5i**, which was subsequently purified by column chromatography ethyl acetate/*n*-hexane (7:3) and collected as an off-white solid in 81% yield; m. p. 318-320°C; ^1^H NMR (400 MHz, DMSO-*d*_6_) *δ* 11.91 (s, 1H, NH), 10.17 (s, 1H, NH), 8.27 (dt, *J* = 25.1, 9.6 Hz, 3H, C=CHN+2Ar-H), 7.82 – 7.60 (m, 2H), 7.50 (dd, *J* = 8.1, 4.4 Hz, 1H), 7.47 – 7.35 (m, 2H), 7.24 – 7.11 (m, 2H), 3.94 – 3.76 (m, 4H, 2-CH_2_O-), 3.76 – 3.64 (m, 4H, 2-CH_2_N-); ^13^C NMR (101 MHz, DMSO-*d*_6_) *δ* 183.0, 165.1, 163.2 (3C = N, Triaz.), 154.2, 142.7 (C_pyraz._), 138.6, 136.8, 131.6, 129.2 (2Ar-C), 126.9, 123.1, 122.0 (2Ar-C), 121.7, 116.4 (2Ar-C), 112.5, 103.8, 66.4 (2CH_2_O-), 44.3 (2CH_2_N-); Anal. Calc. for C_25_H_20_ClN_9_O (497.95); C, 60.30; H, 4.05; N, 25.32. Found C, 60.27; H, 4.13; N, 25.28; HRMS-ESI (m/z) calculated for [M + H]^+^ 498.95; found: 499.0065.

##### 2.1.1.25 1-(4-((4-Bromophenyl)amino)-6-morpholino-1,3,5-triazin-2-yl)-5-(*1H*-indol-3-yl)-*1H*-pyrazole-4-carbonitrile, 5j

*N-*(4-Bromophenyl)-4-hydrazinyl-6-morpholino-1,3,5-triazin-2-amine **2j**, 3 (dimethylamino)-2-(*1H*-indole-3-carbonyl)acrylonitrile **4**, and *p*-toluenesulfonic acid were used in the general procedure (GP3.2) to obtain the desired pyrazolyl-*s*-triazine derivative **5j** as an off-white solid in 72% yield; m. p. 218-220°C; ^1^H NMR (500 MHz, DMSO-*d*_6_) δ 11.69 – 11.29 (m, 1H, NH), 8.56 (s, 1H, C=CHN), 8.12 (s, 2H), 7.88 (s, 2H), 7.47 (s, 2H), 7.19 – 7.07 (m, 3H), 3.47-3.27 (s, 8H, overlapping of 2-CH_2_O- and 2-CH_2_N-); ^13^C NMR (126 MHz, DMSO-*d*_6_) δ 174.4, 162.8, 162.2 (3C = N, Triaz.), 155.9, 148.8, 144.7, 136.7, 124.7, 122.8 (2Ar-C), 121.6, 120.6 (2Ar-C), 116.4, 112.5, 107.0, 105.4, 87.0, 66.5 (2CH_2_O-), 43.5 (2CH_2_N-). Anal. Calc. for C_25_H_20_BrN_9_O (542.40); C, 55.36; H, 3.72; N, 23.24. Found C, 55.41; H, 3.67; N, 23.20; HRMS-ESI (m/z) calculated for [M + H]^+^ 543.40; found: 543.5009.

##### 2.1.1.26 5-(1*H*-Indol-3-yl)-1-(4-((4-methoxyphenyl)amino)-6-morpholino-1,3,5-triazin-2-yl)-1*H*-pyrazole-4-carbonitrile, 5k

4-Hydrazinyl-*N-*(4-methoxyphenyl)-6-morpholino-1,3,5-triazin-2-amine **2k,** 3-(dimethylamino)-2-(1*H*-indole-3-carbonyl)acrylonitrile **4**, and *p*-toluenesulfonic acid were use in the general procedure (GP2) to obtain the target pyrazolyl-*s*-triazine derivative **5k** as an off-white precipitate in 82% yield; m. p. 293-295°C; ^1^H NMR (400 MHz, DMSO-*d6*) *δ* 11.93 (s, 1H, NH), 9.95 (s, 1H, NH), 8.43 (s, 1H, C=CHN), 8.35 – 8.26 (m, 3H), 7.61 (d, *J* = 8.5 Hz, 1H), 7.50 (d, *J* = 7.7 Hz, 1H), 7.29 – 7.15 (m, 2H), 6.95 (d, *J* = 8.8 Hz, 2H), 3.81 (s, 4H, 2-CH_2_O-), 3.74 (s, 3H, OCH_3_), 3.68 (s, 4H, 2-CH_2_N-); ^13^C NMR (101 MHz, DMSO-*d6*) *δ* 183.1, 165.3, 163.6 (3C = N, Triaz.), 155.6, 154.3, 142.6, 136.8, 132.6, 131.6, 127.0, 123.2, 122.1, 116.6, 114.6, 112.6, 103.9, 66.5 (2CH_2_O), 55.7 (d, *J* = 13.4 Hz, OCH_3_), 44.3 (2CH_2_N). Anal. Calc. for C_26_H_23_N_9_O_2_ (493.53); C, 63.28; H, 4.70; N, 25.54. Found: C, 63.18; H, 4.66; N, 25.64; HRMS-ESI (m/z) calculated for [M + H]^+^ 494.53; found: 494.6096.

### 2.2 Biology

#### 2.2.1 Cell culture

The parental MCF7 (breast cancer cell line), A549 (non-small cell lung cancer cell line), and human dermal fibroblast (HDF) (human dermal cell line) cells were obtained from the American Type Culture Collection (ATCC, Manassas, VA, United States). MCF7 and A549 cells were cultured in RPMI 1640 medium (EuroClone, Italy) supplemented with 10% (v/v) heat-inactivated fetal bovine serum (FBS) (EuroClone, Italy), 1% penicillin-streptomycin (EuroClone, Italy), and 2 mM l-glutamine.

#### 2.2.2 Cytotoxicity using MTT assay

The cytotoxic activity of the tested compounds were tested using MTT assay. Different concentartions (0.01-100 µM) were employed and the absorbance of the solution was measured at 560 nm using a Glomax plate reader (Promega, United States). Then, the cell viability was recored and IC_50_ values were calcualted. (Mosmann, 1983; Nafie et al., 2020c).

#### 2.2.3 EGFR/CDK2 enzyme inhibition

EGFR-TK assay kit (ADP-Glo™ kinase assay, Cat No. V9261, Promega, United States) kinase assay was performed to evaluate the capacity of **3h**, **3i**, and **3j** to inhibit EGFR. The percentage of autophosphorylation inhibition by compounds was calculated using the curves of percentage inhibition of eight concentrations of each compound, IC_50_ was calculated using the GraphPad prism7 software (Hisham et al., 2019).

#### 2.2.4 Flow cytometry using annexin V/PI staining

A549 cells were seeded into 6-well culture plates (3-5× 10^5^ cells/well) and incubated overnight. Cells were then treated for 48 h with **3h**, **3i**, and **3j** at their IC_50_ values. Next, media supernatants and cells were collected and rinsed with ice-cold PBS. The cells were then suspended in the flow cytometry solutions and cells were stained with Annexin V/PI, and data was acquired using a Cytoflex FACS machine. Data were analyzed using cytExpert software. (Hisham et al., 2019; Khodair et al., 2019; Nafie et al., 2020b).

#### 2.2.5 Gene expression analysis using RT-PCR

Gene expression analyis for the most active candidate **3i** was invetsigated in untreated and treated A549 cells, for determination of the pro-apoptotic genes P53, Bax, and Caspases 3, 8, and 9, and the anti-apoptotic gene Bcl-2, (Mosmann, 1983; Hisham et al., 2019; Nafie et al., 2020c)**.**

### 2.3 *In Silico* studies

#### 2.3.1 Molecular docking

In this study, AutoDock Vina (Trott and Olson, 2009) software was used to dock the compounds under investigation to the EGFR (PDB = 1M17) and CDK-2 (PDB = 2A4L) protein structures, respectively. Maestro was used to optmize protein and ligand structures and favor them energetically. Next, grid-box dimensions were used to identify the locations of the co-crystallized ligands inside the proteins. Results from molecular docking were interpreted by binding activities in terms of binding energy and ligand-receptor interactions. Chimera was used to complete the visualization (Petrescu et al., 2000).

#### 2.3.2 ADME pharmacokinetics

Following the procedures outlined in (Nafie et al., 2020a; Nafie and Boraei, 2022), the ADME pharmacokinetics parameters of the most effective drugs were determined using a suite of software that included the websites “MolSoft,” “Molinspiration,” and “SwissADME."

## 3 Results and discussion

### 3.1 Chemistry

A new series of pyrazolyl-indole-*s*-triazine derivatives were prepared via two synthetic approaches. *Mono*-hydrazinyl-*s*-triazine derivatives were used as a common starting material in both synthetic pathways and were prepared following the method reported in (Farooq et al., 2019; Shawish et al., 2022a; Shawish et al., 2022b). In addition, the first synthetic approach involved another starting material, namely cyanoacetyl indole **1**, which was prepared following the method described by Bergman and coworkers (Bergman et al., 2004). In contrast, the second approach required the building block enaminonitrile-indole **4**, which was obtained from cyanoacetyl indole **one** and DMF-DMA following the method reported by M. Radwan and coworkers (Radwan and El-Sherbiny, 2007).

**SCHEME 1:**
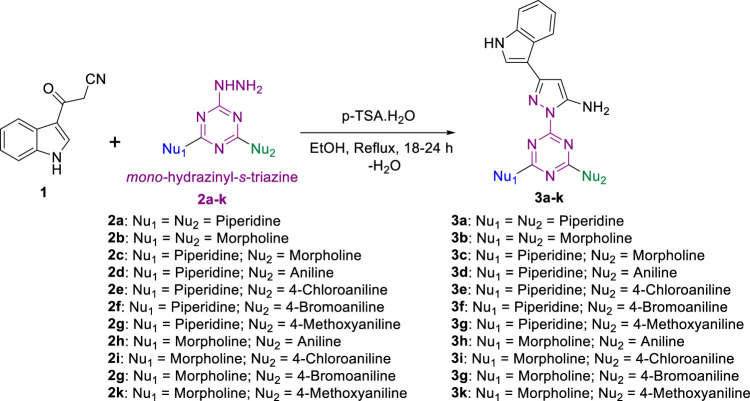
Synthesis of pyrazolyl-amine-indole-*s*-triazine derivatives **3a-k.**

Initially, **one** was reacted with several mono-hydrazinyl-*s*-triazine derivatives **2a-k** in the presence of *p*-toluenesulfonic acid (TsOH) to render the corresponding pyrazolyl-amino-indole-*s*-triazine derivatives **3a-k**, as depicted in Scheme 1, following the reported method with slight modifications (Bergman et al., 2004). Optimal reaction conditions were achieved by using ethanol (EtOH) as solvent and by activating the carbonyl group *via* TsOH as a catalyst. The reaction involved using 1 equiv. of each reactant and refluxing the mixture for 18-24 h. The progress of the reaction was monitored by TLC [ethyl acetate (EtOAc)/*n*-hexane 8:2 or MeOH/CHCl_3_, 1:9]. The resulting target derivatives **3a-k** were collected in good yield (82-94%) and without the need for further purification.

**SCHEME 2:**
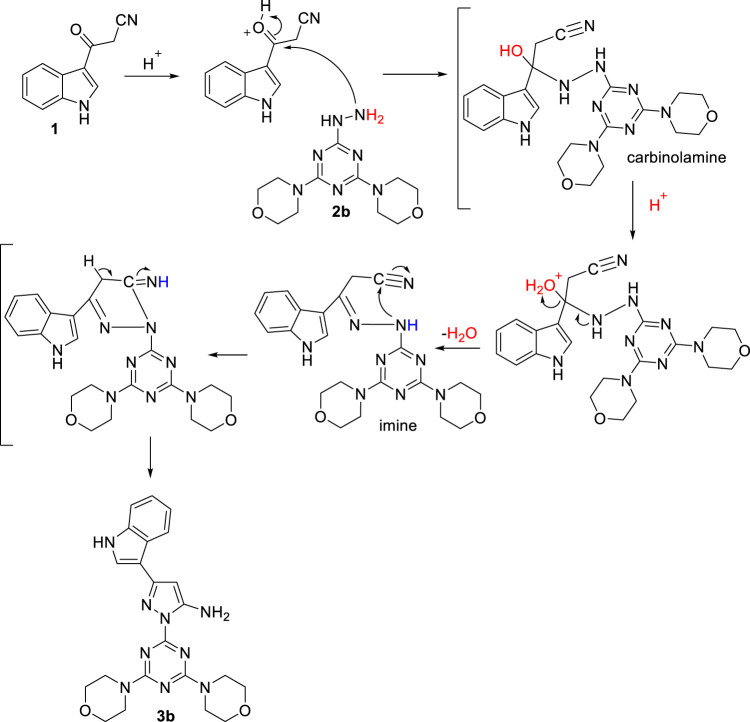
Proposed reaction mechanism for the formation of **3b**.

To illustrate the reaction mechanism of this pathway, 1-(4,6-dimorpholino-1,3,5-triazin-2-yl)-3-(1*H*-indol-3-yl)-1*H*-pyrazol-5-amineincludes **3b** was selected as a representative model. The first step reaction mechanism includes a nucleophilic addition of the terminal primary amino group of the hydrazine derivative **2b** to the carbonyl group to produce the carbinolamine intermediate, which is subsequently protonated by the catalytic acid, followed by H_2_O elimination to give the corresponding imine intermediate. Simultaneously, an addition reaction of the internal nitrogen of hydrazine to the carbon atom of the nitrile group takes place to produce the target pyrazolyl-amine-indole-*s*-triazine derivative **3b**, as shown in Scheme 2.

The structural elucidation of **3b** was performed using several spectroscopic techniques, which revealed full agreement with the proposed structure. The ^1^H-NMR spectrum (*Supporting materials*) of **3b** shows a triplet peak at *δ* 3.68 ppm, which corresponds to the eight methylene protons adjacent to the nitrogen of morpholine, and another signal at *δ* 3.71-3.98 ppm, assigned to the eight protons of methylene next to the oxygen of morpholine. The pyrazolyl proton appears at the chemical shift of 5.78 ppm, while the protons of the amine group appear as singlet at 6.7 ppm. The four phenyl protons of the indole appear at 7.11 (2H), 7.41 (1H), and 7.77 (1H), respectively. The characteristic downfield signal at 8.34 ppm corresponds to the α-H with respect to the nitrogen in the indole moiety (N-C**H** = C). The ^13^C-NMR spectrum of **3b** (*Supporting materials*) shows characteristic signals at *δ* 165.0 and 163.6 ppm, which are assigned to the triazine carbons. The pyrazolyl carbon that carries the amino group appears to be de-shielded at 151.3 ppm due to the inductive influence of the amino group, while the relatively upfielded shifts at 122.1 and 86.1 ppm correspond to the two other pyrazolyl carbons. The signals between *δ* 109.7 and150.3 are assigned to the eight carbons of the indole moiety. The signal at *δ* 66.5 ppm corresponds to morpholine carbons adjacent to oxygen, while the signal at 44.1 ppm is assigned to the two morpholine carbons adjacent to the nitrogen atom.

In addition, the high-resolution mass analysis of **3b** fully agrees with the proposed structure, with the [M + H]^+^ peak at m/z 448.4691, which is consistent with the calculated value of m/z 448.50.

**SCHEME 3:**
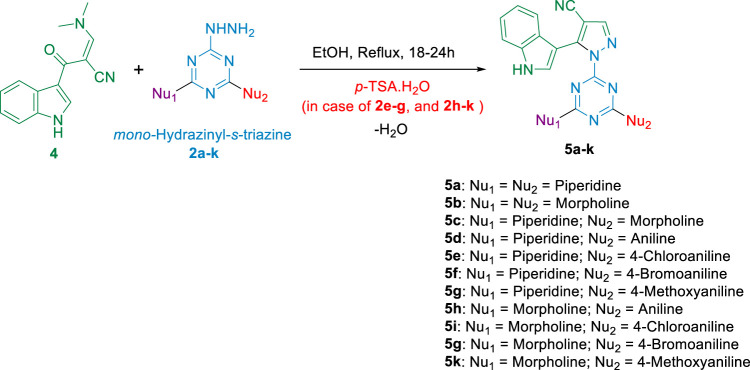
Synthesis of pyrazolyl-carbonitrile-indole-*s*-triazine derivatives **5a-k**.

Next, the second set of the desired compounds **5a-k** was prepared by treating enaminonitrile-indole **4** with aliphatic nitrogen containing hydrazinyl-*s*-triazine derivatives **2a-c** in refluxed EtOH for 18-24 h and monitored by TLC to afford the target pyrazolyl-carbonitrile-indole-*s*-triazine derivatives **5a-c,** which were collected in a very good to excellent yield (78-88%) **(** Scheme 3 **)**. When the previous conditions were applied for aniline- and *p*-substituted-aniline-hydrazinyl-*s*-triazine derivatives **2d-k**, the reaction yield was very low due to the formation of a mixture of side products, as indicated by TLC analysis. To overcome this issue, a modified procedure was applied using *p-*TSA as a catalyst to activate the water elimination step in the reaction mechanism, which in turn significantly improved the progress of the reaction. This improvement was evidenced by TLC, which showed single bright blue spots corresponding to the target products **5d-k**, which were subsequently collected in a good yield and without the need for further purification in most cases **(** Scheme 3 **)**.

**SCHEME 4:**
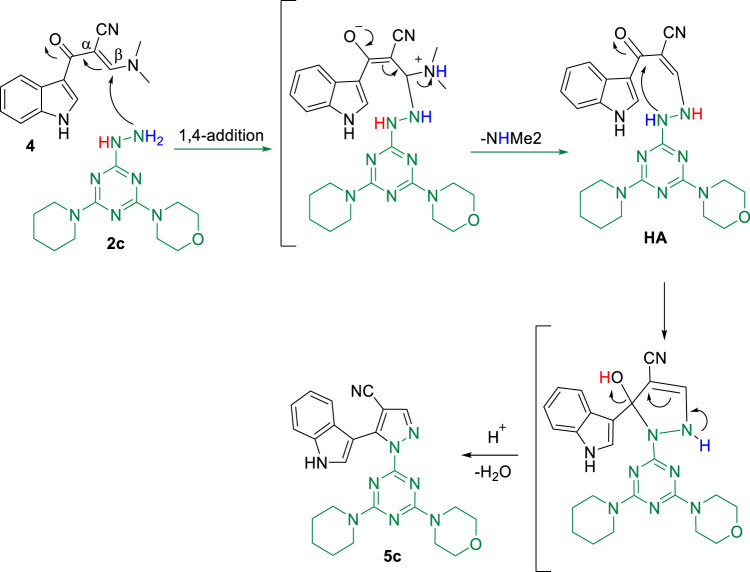
Proposed reaction mechanism for the desired compound **5c** as a model example.

According to our assumption, the formation of the final compounds proceeded as described next. The initial step involves nucleophilic 1,4-addition of the terminal amino-nitrogen of hydrazinyl-*s*-triazine derivative **2c** to the *β* carbon of the enamine intermediate **4**, followed by amine-exchange of the dimethylamino group to form the hydrazinyl-acrylonitrile adduct intermediate **(HA)**. Subsequently, intramolecular cyclodehydration takes place to produce the target pyrazolyl-carbonitrile-indole-*s*-triazine derivative **5c**, as illustrated in **(** Scheme 4 **)**.

The spectral data collected by NMR (^1^H and ^13^C), IR, and MS are all consistent with the proposed structure of **5c**. The ^1^H-NMR spectrum (*Supporting materials*) reveals three signals corresponding to methylene protons of the piperidinyl moiety at *δ* 1.03, 1.33, and 1.42 ppm, respectively. The signals at *δ* 3.10 ppm and 3.60 ppm correspond to methylene protons adjacent to nitrogen and oxygen in the piperidinyl and morpholino moieties, respectively. The five indole protons and the pyrazolyl proton appear between 6.9 and 8.3 ppm, and the singlet signal at *δ* 8.30 ppm is characteristic of α-H with respect to nitrogen in the indole moiety (N-C**H** = C). The downfield shift at *δ* 11.65 ppm is assigned to the N**H** proton of the indole. The ^13^C-NMR spectrum of **5c** (*Supporting materials*) shows a signal at *δ* 163.2, 164.6, and 165.0 ppm, which correspond to triazine carbons. The nitrile carbon and the indole carbons are represented by the 11 signals between 94.7 and 145.8 ppm, considering that the signal at *δ* 94.7 ppm is characteristic of the pyrazolyl carbon bearing the nitrile group. The signal at *δ* 66.2 ppm is assigned to the two carbons adjacent to oxygen in the morpholino moiety, while the carbons adjacent to nitrogen on the morpholino and piperidinyl moieties appear at *δ* 43.7 and 44.2 ppm, respectively. In addition, the high-resolution mass analysis of **5c** shows the [M + H]^+^ peak at m/z 456.4899, which is consistent with the calculated value of m/z 456.53.

### 3.2. Biology

#### 3.2.1. *In vitro* antiproliferative activity and cytotoxicity assay

**FIGURE 3:**
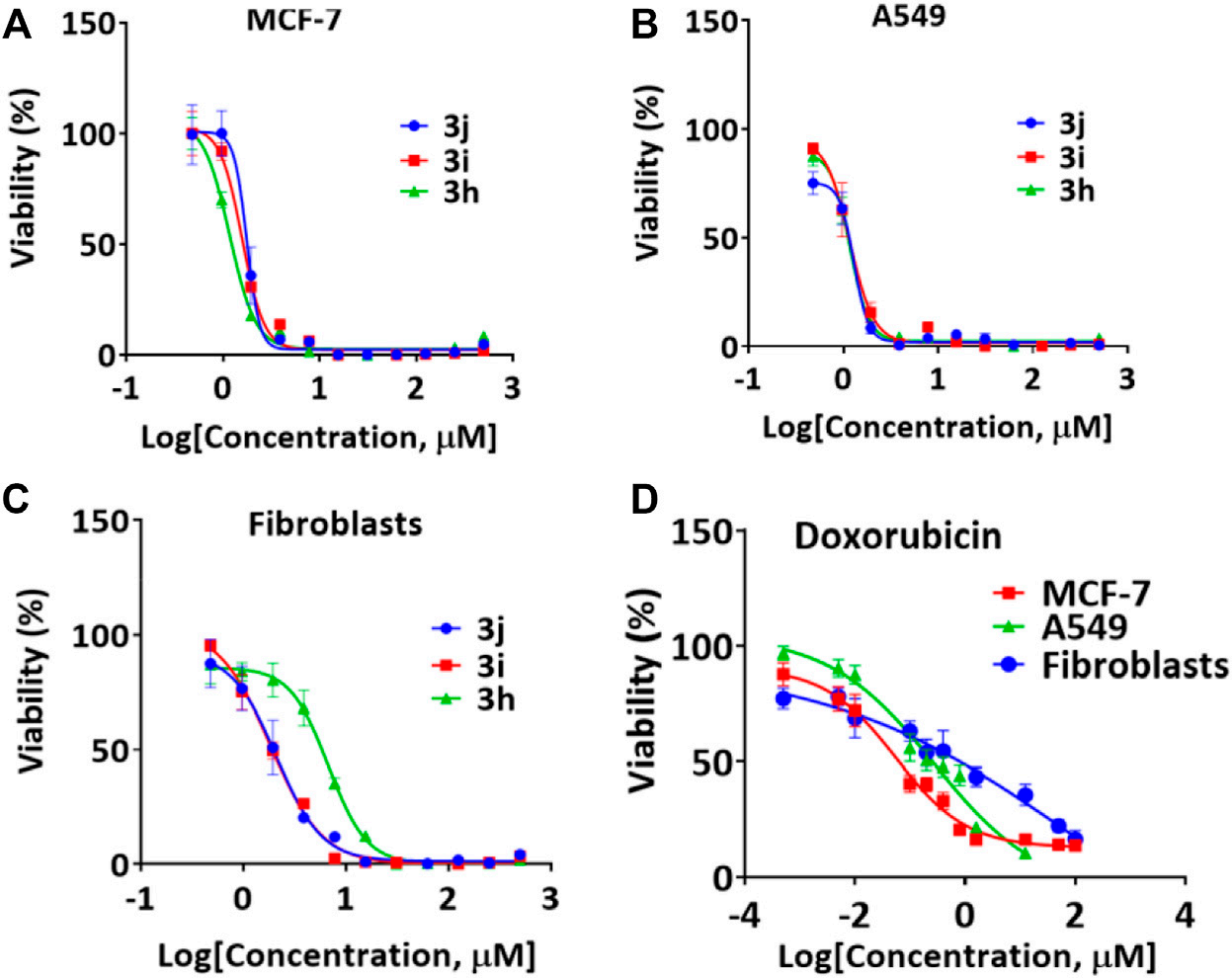
Representative cell viability results used to calculate the IC_50_ values for compounds **(A) 3h (B) 3i**, **(C) 3j**, and **(D)** Doxorubicin.

We performed a set of biological studies to determine the potential bioactivity of pyrazolyl-amine-indole-*s*-triazines **3a-k** and pyrazolyl-carbonitrile-indole-*s*-triazine derivatives **5a-k**. The antiproliferative activity of **3a-k** and **5a-k** was studied against two cancer cell lines, namely A549 (non-small cell lung cancer) and MCF-7 (breast cancer), and against HDFs (non-cancerous human dermal fibroblasts). Cell viability assays revealed selective anticancer activity of most of the compounds compared with the HDFs (Table 1; Figure 3). However, 4-(5-amino-3-(1*H*-indol-3-yl)-1*H*-pyrazol-1-yl)-6-morpholino-*N*-phenyl-1,3,5-triazin-2-amine **3h**, 4-(5-amino-3-(*1H*-indol-3-yl)-1*H*-pyrazol-1-yl)-*N*-(4-chlorophenyl)-6-morpholino-1,3,5-triazin-2-amine **3i**, and 4-(5-amino-3-(*1H*-indol-3-yl)-1*H*-pyrazol-1-yl)-*N*-(4-bromophenyl)-6-morpholino-1,3,5-triazin-2-amine **3j** showed the most potent activity, with the following IC_50_ values: **3h** [IC_50_ = 2.65 ± 0.22 μM/ml (A549); 2.66 ± 0.26 μM/ml (MCF-7); 14.00 μM/ml (HDFs)]; **3i** [IC_50_ = 2.40 ± 0.64 μM/ml (A549); 3.28 ± 0.16 μM/ml (MCF-7); 3.78 ± 0.55 μM/ml (HDFs)]; and **3j** [IC_50_ = 2.32 ± 0.21 μM/ml (A549); 3.30 ± 0.32 μM/ml (MCF-7); 3.96 ± 0.73 μM/ml (HDFs)]. Interestingly, these three active derivatives belong to pyrazolyl-amine-indole-*s*-triazine series and they share a morpholine moiety accompanied by aniline or *p*-substituted aniline (*p*-Cl or *p*-Br). The IC_50_ values of these three compounds against the A549 and MCF-7 cell lines were relatively higher than those reported for the reference drug doxorubicin [IC_50_ = 0.28 ± 0.11 μM/ml (A549); 0.06 ± 0.02 μM/ml (MCF-7)], thereby indicating lower activity of these derivatives against the cancer cells than the reference drug. However, the IC_50_ values of **3h, 3i,** and **3j** against HDFs (14.00 ± 2.67, 3.78 ± 0.55, and 3.96 ± 0.73 μM/ml, respectively) were markedly higher than the those reported for doxorubicin (0.42 ± 0.27 μM/ml). This observation indicates that the toxicity of the prepared derivatives is significantly less towards healthy cells than doxorubicin and that these novel compounds therefore have a better therapeutic index. On the other hand, most of the pyrazolyl-carbonitrile-indole-*s*-triazine derivatives **5a-k** showed low activity against the cancer cells (Table 2). By comparison of the chemical strutcures of the series of **3a-k** to the **5a-k**; the presence of the amino-group may be has good effect on the reactivity rather than the nitrile group exist in the **5a-k** series.

**TABLE 1:** IC_50_ values of the tested compounds **3a-k** after the MTT assay showing variable responses of the treated cell lines.

Anticancer activity against Human Cancer Cell lines IC_50_ ± STDEV (µM/ml)*			
Entry	A549	MCF-7	HDFs
3a	>500	>500	>500
3b	3.08 ± 1.27	11.66 ± 1.77	4.47 ± 1.43
3c	4.82 ± 0.29	12.61 ± 1.03	6.82 ± 1.34
3d	10.56 ± 2.88	11.73 ± 4.43	22.88 ± 5.20
3e	9.57 ± 1.26	15.22 ± 0.74	11.23 ± 0.69
3f	14.83 ± 0.83	13.20 ± 0.55	6.8 ± 1.02
3g	6.23 ± 0.48	7.68 ± 0.21	4.36 ± 0.83
3h	2.65 ± 0.22	2.66 ± 0.26	14.00 ± 2.67
3i	2.40 ± 0.64	3.28 ± 0.16	3.78 ± 0.55
3j	2.32 ± 0.21	3.30 ± 0.32	3.96 ± 0.73
3k	3.64 ± 1.26	4.11 ± 0.50	8.9 ± 1.07
Doxorubicin	0.28 ± 0.11	0.06 ± 0.02	0.42 ± 0.27

* IC_50_ values ware expressed as the average ±SD of four independent experiments

**TABLE 2:** IC_50_ values of the tested compounds **5a-k** after the MTT assay showing variable responses of the treated cell lines.

Anticancer activity against Human Cancer Cell lines IC_50_ ± STDEV (µM/ml)*			
Entry	A549	MCF-7	HDFs
5a	66.13 ± 5.71	65.25 ± 1.43	>500
5b	>500	>500	>500
5c	222.71 ± 88.30	152.20 ± 50.07	537.48 ± 71.61
5d	10.33 ± 2.64	16.83 ± 0.78	114.62 ± 14.78
5e	>500	>500	>500
5f	>500	>500	>500
5g	>500	>500	>500
5h	7.62 ± 0.85	8.52 ±0 .22	23.81 ± 3.37
5i	44.54 ± 19.24	59.83 ± 0.81	>500
5j	23.17 ± 2.27	51.06 ± 10.27	>500
5k	>500	>500	>500
Doxorubicin	0.28 ± 0.11	0.06 ± 0.02	0.42 ± 0.27

* IC_50_ values ware expressed as the average ±SD of four independent experiments

#### 3.2.2 Enzyme targeting

To study effective molecular docking, the inhibitory capacity of **3h, 3i,** and **3j** was tested against EGFR and CDK-2. Interestingly, **3i** showed potent EGFR inhibition, with an IC_50_ value of 34.1 nM compared to Erlotinib (IC_50_ = 67.3 nM), causing 93.6% and 91.4% inhibition of EGFR and CDK-2, respectively, at 10 µM (Table 3). In addition, **3j** and **3h** revealed IC_50_ values of 68.4 and 64.2 nM as EGFR inhibitors and 136.4 and 112.2 nM as CDK-2 inhibitors. These findings highlight the promising cytotoxicity in tested cancer cells and validate the designed synthetic strategy proposed.

**TABLE 3:** IC_50_ of the tested compounds against EGFR and CDK-2.

Compound	EGFR inhibition IC_50_ [nM]*	CDK-2 inhibition [nM]*
3 h	64.2 ± 3.12	112.2 ± 3.01
3i	34.1 ± 1.58	108.3 ± 3.12
3j	68.4 ± 2.98	136.4 ± 3.14
Erlotinib	67.3 ± 2.54	-
Roscovitine	-	140 ± 3.4

* Values are expressed as Mean ± SD, of three independent replicates. IC_50_ values were calculated using sigmoidal non-linear regression curve fit of percentage inhibition against five concentrations of each compound.

#### 3.2.3 Flow cytometry

**FIGURE 4:**
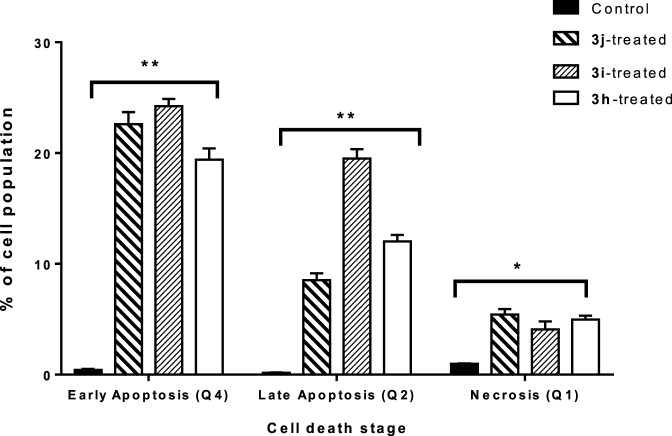
Bar representation of the percentage of early, intermediate, and late apoptotic cell population in untreated and **3j-**, **3i-**, and **3h-**treated A549 cells, with the IC_50_ value at 48 h, using Annexin V/Propidium Iodide staining “Q1 (intermediate apoptosis, AV-/PI+), Q2 (late apoptotic cells, AV+/PI+), Q3 (normal cells, AV-/PI-), and Q4 (early apoptotic cells, AV+/PI-). Data shown are the average of three independent experimental runs (Mean ± SD).

The capacity of **3h**, **3i**, and **3j** to induce apoptosis in A549 cancer cells was tested using Annexin V/PI staining (IC_50_ = 1.2 µM, 48 h). Compound **3j** enhanced total apoptotic lung cancer cell death 50.9-fold (31.1% compared to 0.61% for the control) (Figure 4). The percentage of cells undergoing early apoptosis increased to 22.6%, and the percentage of those undergoing late apoptosis to 8.5% compared to the 0.42% and 0.16%, respectively, observed in the control group. In addition, **3j** caused a 5.58-fold increase in necrosis-mediated cell death (5.42% compared to 0.97% for the control). Compound **3i** enhanced total apoptotic lung cancer cell death 71.6-fold (43.7% compared to 0.61% for the control). The percentage of cells undergoing early apoptosis was increased to 24.2%, and the percentage of those undergoing late apoptosis to 19.5%. In addition, this compound caused a 4.2-fold increase in necrosis-mediated cell death (4.08% compared to 0.97% for the control). Compound **3h** enhanced total apoptotic lung cancer cell death 51.4-fold (31.4% compared to 0.61% for the control). The percentage of cells undergoing early apoptosis increased to 19.4%, and the percentage of those undergoing late apoptosis to 12%. In addition, this compound caused a 5.1-fold increase in necrosis-mediated cell death (4.98% compared to 0.97% for the control). Consequently, compound **3i** greatly induced apoptosis in A549 cells and showed a greater ratio than **3j** and **3h** This finding is consistent with the dual enzymatic target’s inhibition and cytotoxic activity.

#### 3.2.4 RT-PCR

**FIGURE 5:**
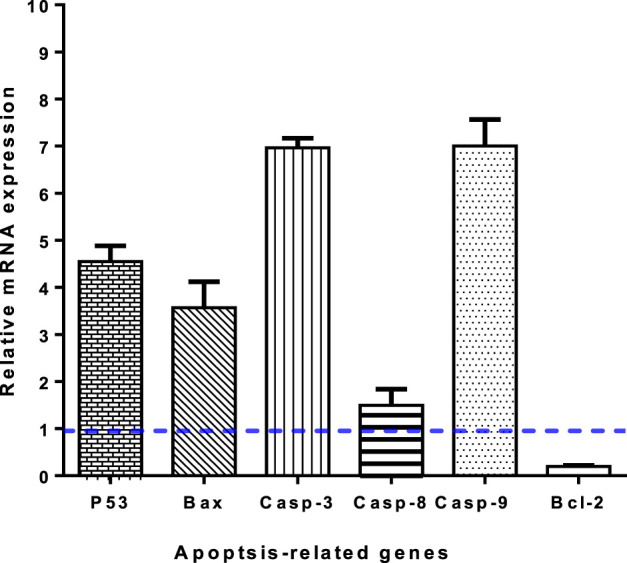
Gene expression levels of apoptosis-related genes of untreated and **3i-**treated A549 cells with the IC_50_ value. Values are expressed as Mean ± SD of three independent replicates. Data were normalized using β-actin as house-keeping gene. The dashed line represents the gene expression level of untreated control. Fold change was calculated using 2^^- ΔΔCT^

To validate apoptotic cell death, RT-PCR was performed on both untreated and treated A549 cells (Figure 5). Treatment with **3i** led to an increase in the expression of pro-apoptotic genes P53, Bax, caspases 3, 8, and 9, with a fold of change of 4.55, 3.56, 6.96, 1.49, and 7, respectively. At the same time, it led to a decrease in the expression of the anti-apoptotic gene Bcl-2, with a 0.23-fold change. These results are consistent with apoptosis-inducing activity through enzyme inhibition. Caspases are essential for both the beginning and end of the cell death process during mitochondria-mediated apoptosis. Loss of mitochondrial potential (ΔΨm) can be induced by upregulating pro-apoptotic components at the expense of anti-apoptotic proteins like Bcl-2. The release of cytochrome c and the loss of mitochondrial potential are two outcomes of activating the intrinsic apoptotic pathway, which can be triggered by increasing pro-apoptotic proteins over anti-apoptotic ones, thereby triggering cascade reactions of caspases 3 and 9, which lead to cell death through caspase-dependent apoptosis.

#### 3.2.5 Molecular docking

CDK-2 and EGFR are involved in signaling pathways that regulate cell survival and death, respectively, and they may provide novel targets for effective therapies for patients with lung cancer. A molecular docking study was performed to highlight the virtual mechanism of binding to EGFR and CDK-2. Cocrysatllized liagnd of EGFR protein formed a H-bond interaction with Met 769 inside its activ site, while the Cocrysatllized liagnd of CDK-2 formed ion-dipole interaction with Lys 89 inside its active site. Both amino acids represents the active residues for EGFR and CDK-2 proteins.

**FIGURE 6:**
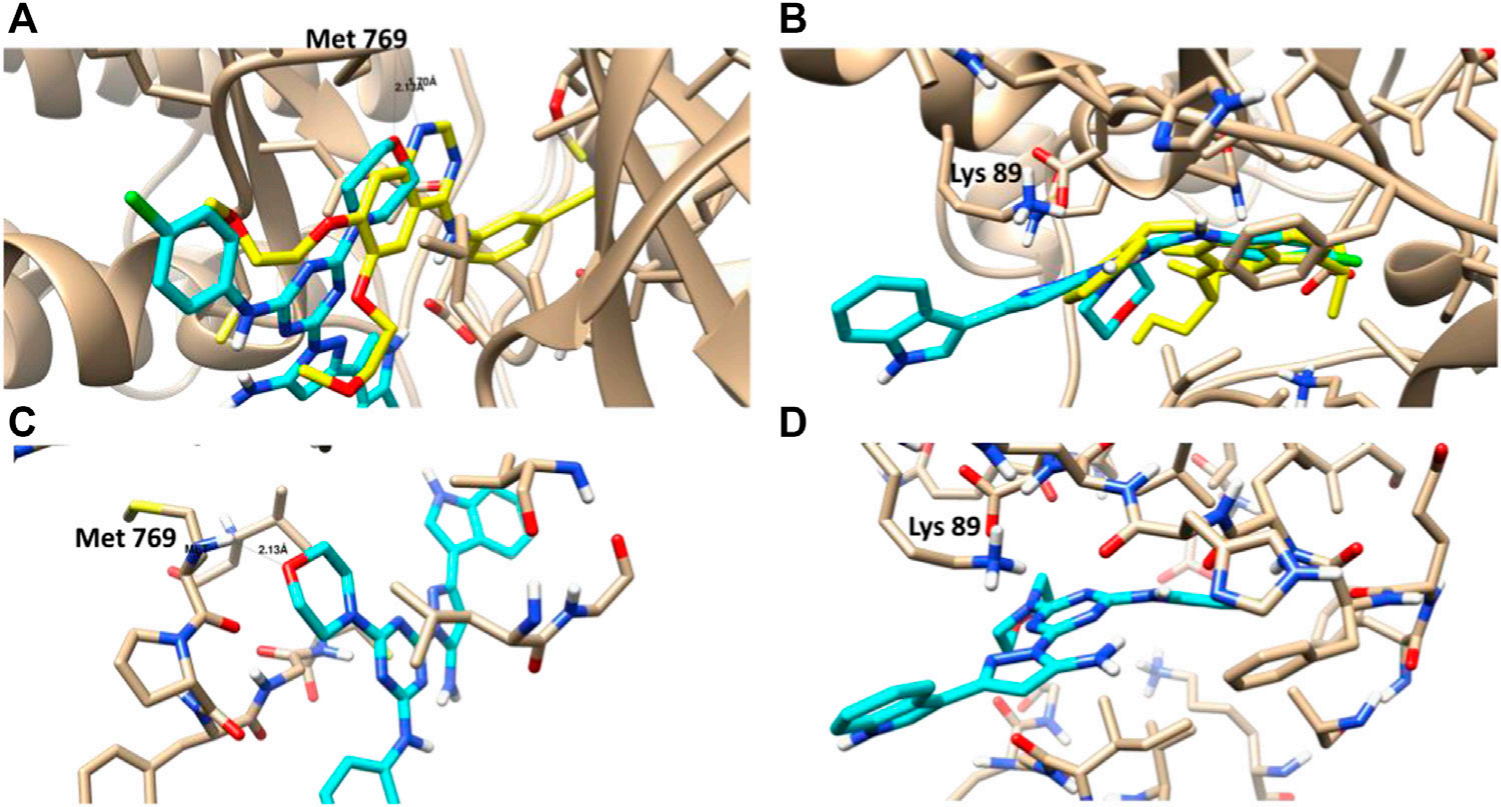
**(A,B)**: Molecular representation of the docked compound **3i** (Cyan) with the co-crystallized ligand (yellow) towards the EGFR and CDK-2 proteins. **(C,D)**: Interactive binding mode of the docked compound **3i** with the key amino acids; Met 769 for EGFR and Lys 89 for CDK-2.

Compound **3i** showed good binding affinity to EGFR and CDK-2, with binding energies of -23.6 and -21.3 kcal/mol, and it formed good interactions with the key amino acids of these two proteins, such as the co-crystallized ligands (Figure 6). Compound 3i formed a hydrogen bond with Met 769 inside the EGFR protein as H-bond acceptor with bond length (2.1 Å), and it formed ion-dipole interaction with Lys 89 inside the CDK-2 protein like the co-crystazllized ligands of both proteins. Inhibitory activities of compound **3i** against EGFR and CDK-2 activities matched with its binding affinities towards both proteins illustaretd by docking studies. Therefore, the docking results highlighted the pyrazolyl-*s*-triazine with indole motif for interactions with targeted proteins EGFR and CDK-2, as illustrated by the 3D binding disposition.

#### 3.2.6 ADME pharmacokinetics

Compounds **3i** and **3h** with highlighted cytotoxic and EGFR/CDK2 activity were further studied for their physicochemical properties and drug-likeness. The compounds, especially **3i**, showed promising values according to Lipinsiki’s rule of five of “molecular weight, number of rotatable bonds, H-bond donor, and acceptors along with number of violations” (Table 4).

**TABLE 4:** Molecular properties of and drug-likeness.

#	Molsoft			Molinspiration 2018.10					SwissADME
	HBA	HBD	Solubility (mg/L)	MWt (D)	MV (A^3^)	PSA (A^2^)	nrotb	nviolations	Drug likeness (Lipinski Pfizer filter)
3i	5	4	0.64	487.9	411.3	122.8	5	0	“Yes, drug like”
MW ≤ 500, HBA ≤10 and HDD ≤5									
3 h	5	4	4.2	453.5	397.8	122.8	5	0	

“Mwt: Molecular Weight, MV: molecular volume, PAS: polar surface area, nrotb: number of rotatable bonds, nviolations: number of violations, HBA: hydrogen bond acceptor, HBD: Hydrogen Bond Donor".

## 4 Conclusion

In conclusion, a series of novel analogs of pyrazolyl-indole-*s*-triazine derivatives were synthesized and achieved in high chemical yields. An MTT assay was used to determine the antitumor activity and safety profiles of the target compounds in two cancer cell lines, namely A549 and MCF-7 cells, as well as in HDFs. The IC_50_ values revealed that the pyrazolyl-amine-indole-*s*-triazine derivatives **3h, 3i,** and **3j** exhibited potent activity against the cancer cell lines. In addition, regarding the safety profile, these three compounds outperformed the reference drug doxorubicin in HDFs, showing IC_50_ values between 3.96 and 14.00 µM (IC_50_ of 0.42 µM for doxorubicin). Furthermore, **3i** exhibited remarkable inhibitory activity against EGFR, with an IC_50_ value of 34.1 nM compared to the reference drug Erlotinib (IC_50_ value of 67.3 nM). Consequently, at 10 μM, **3i** inhibited EGFR by 93.6% and CDK-2 by 91.4%. In addition, the flow cytometry study revealed that **3i** enhanced the total lung cancer apoptosis 71.6-fold, which represents an increase of 43.7% compared to 0.61% in the control. On the basis of our findings, we propose that the novel synthesized derivatives **3h, 3i,** and **3j** be considered as lead compounds for further research into target-oriented anticancer agents.

## Data Availability

The original contributions presented in the study are included in the article/Supplementary Material further inquiries can be directed to the corresponding authors.
