# Corrigendum: Pyrazolyl-s-triazine with indole motif as a novel of epidermal growth factor receptor/cyclin-dependent kinase 2 dual inhibitors

**DOI:** 10.3389/fchem.2022.1131801

**Published:** 2023-01-11

**Authors:** Ihab Shawish, Mohamed S. Nafie, Assem Barakat, Ali Aldalbahi, Hessa H. Al-Rasheed, M. Ali, Walhan Alshaer, Mazhar Al Zoubi, Samha Al Ayoubi, Beatriz G. De La Torre, Fernando Albericio, Ayman El-Faham

**Affiliations:** ^1^ Department of Math and Sciences, College of Humanities and Sciences, Prince Sultan University, Riyadh, Saudi Arabia; ^2^ Department of Chemistry, College of Science, King Saud University, Riyadh, Saudi Arabia; ^3^ Department of Chemistry, Faculty of Science, Suez Canal University, Ismaïlia, Egypt; ^4^ Cell Therapy Center, The University of Jordan, Amman, Jordan; ^5^ Department of Basic Medical Sciences, Faculty of Sciences, Yarmouk University, Irbid, Jordan; ^6^ KwaZulu-Natal Research Innovation and Sequencing Platform (KRISP), School of Laboratory Medicine and Medical Sciences, College of Health Sciences, University of KwaZulu-Natal, Durban, South Africa; ^7^ Peptide Science Laboratory, School of Chemistry and Physics, University of KwaZulu-Natal, Durban, South Africa; ^8^ CIBER-BBN (Networking Centre on Bioengineering, Biomaterials and Nanomedicine) and Department of Organic Chemistry, University of Barcelona, Barcelona, Spain; ^9^ Chemistry Department, Faculty of Science, Alexandria University, Alexandria, Egypt

**Keywords:** pyrazolyl-s-triazine, indole, anticancer profile, EGFR/CDK-2, apoptosis

In the original article, there was an error in the **Affiliation** for Samha Al Ayoubi^2^. Instead of “Samha Al Ayoubi ^2^”, it should be “**Samha Al Ayoubi** ^**1**^”.

The authors apologize for this error and state that this does not change the scientific conclusions of the article in any way. The original article has been updated.

