# Supplementary material for: Pyrazolyl-s-triazine with indole motif as a novel of epidermal growth factor receptor/cyclin-dependent kinase 2 dual inhibitors
Source: Front Chem. 2022 Nov 25;10:1078163. doi: 10.3389/fchem.2022.1078163 (PMC9732672; doi:10.3389/fchem.2022.1078163)
Supplement: Supplementary file 1 [file DataSheet1.docx]

**SUPPORTING INFORMATION**

**Pyrazolyl-*s*-Triazine with Indole Motif as a Novel of Epidermal Growth Factor Receptor/ [Cyclin-Dependent Kinase 2 Dual Inhibitors](https://pubmed.ncbi.nlm.nih.gov/30543440/#:~:text=Although%20the%20role%20of%20CDK2,exploit%20in%20anticancer%20drug%20development.)**

**Ihab Shawish ^1,2^, Mohamed S. Nafie ^3^,** **Assem Barakat ^2,^*, Ali Aldalbahi ^2^, Hessa H. Al-Rasheed^2^, M. Ali^2^, Walhan Alshaer ^4^,** **Mazhar Al Zoubi ^5^, Samha Al Ayoubi ^2^, Beatriz G. de la Torre ^6,7^, Fernando Albericio, ^7,8^* Ayman El-Faham ^9,^***

^1^Department of Math and Sciences, College of Humanities and Sciences, Prince Sultan University, P.O. Box 66833, Riyadh 11586, Saudi Arabia[, ishawish@psu.edu.sa](mailto:,%20ishawish@psu.edu.sa) (I.S.); [sayoubi@psu.edu.sa](mailto:sayoubi@psu.edu.sa) (S.A.).

^2^Department of Chemistry, College of Science, King Saud University, P.O. Box 2455, Riyadh 11451, Saudi Arabia, [ambarakat@ksu.edu.sa](mailto:ambarakat@ksu.edu.sa) (A.B.); [aaldalbahi@ksu.edu.sa](mailto:aaldalbahi@ksu.edu.sa) (A.D.); halbahli@ksu.edu.sa (H.H.A-R); [maly.c@ksu.edu.sa](mailto:maly.c@ksu.edu.sa) (M.A.).

^3^Department of Chemistry, Faculty of Science, Suez Canal University, Ismailia 41522, Egypt, [mohamed_nafie@science.suez.edu.eg](mailto:mohamed_nafie@science.suez.edu.eg) (M.S.N.).

^4^Cell Therapy Center, The University of Jordan, Amman 11942, Jordan, walhanjordan@yahoo.com (W.A.).

^5^Department of Basic Medical Sciences, Faculty of Sciences, Yarmouk University, Irbid 211-63, Jordan, mszoubi@yu.edu.jo (M.S.Z.).

^6^KwaZulu-Natal Research Innovation and Sequencing Platform (KRISP), School of Laboratory Medicine and Medical Sciences, College of Health Sciences, University of KwaZulu-Natal, Durban 4041, South Africa, garciadelatorreb@ukzn.ac.za (B.G.T.).

^7^ Peptide Science Laboratory, School of Chemistry and Physics, University of KwaZulu-Natal, Durban 4001, South Africa, albericio@ukzn.ac.za (F.A.).

^8^CIBER-BBN (Networking Centre on Bioengineering, Biomaterials and Nanomedicine) and Department of Organic Chemistry, University of Barcelona, 08028 Barcelona, Spain (F.A.).

^9^Chemistry Department, Faculty of Science, Alexandria University, P.O. Box 426, Ibrahimia, 12321 Alexandria, Egypt, [ayman.elfaham@alexu.edu.eg](mailto:ayman.elfaham@alexu.edu.eg); [aymanel_faham@hotmail.com](mailto:aymanel_faham@hotmail.com) (A.E-F.).

***** Correspondence: [ambarakat@ksu.edu.sa](mailto:ambarakat@ksu.edu.sa) (A.B.); albericio@ukzn.ac.za (F.A.); [ayman.elfaham@alexu.edu.eg](mailto:ayman.elfaham@alexu.edu.eg) (A.E-F.)

**Table of contents**

1. **Material and methods**
2. Selected copy of the (^1^HNMR, ^13^CNMR and MS) spectrum of the synthesized compounds.

**Figure S1** ^1^H NMR of compound **3a**

**Figure S2** ^1^H and ^13^C compound **3b**

**Figure S3**  ^1^H and ^13^C compound **3c**

**Figure S4** ^1^H and ^13^C compound **3d**

**Figure S5** ^1^H and ^13^C compound **3e**

**Figure S6** ^1^H and ^13^C compound **3f**

**Figure S7** ^1^H and ^13^C compound **3g**

**Figure S8** ^1^H and ^13^C compound **3h**

**Figure S9**  ^1^H and ^13^C compound **3i**

**Figure S10** ^1^H and ^13^C compound **3j**

**Figure S11** ^1^H and ^13^C compound **3k**

**Figure S12** ^1^H and ^13^C compound **5a**

**Figure S13** ^1^H and ^13^C compound **5b**

**Figure S14** ^1^H and ^13^C compound **5c**

**Figure S15** ^1^H and ^13^C compound **5d**

**Figure S16** ^1^H and ^13^C compound **5e**

**Figure S17** ^1^H and ^13^C compound **5f**

**Figure S18** ^1^H and ^13^C compound **5g**

**Figure S19** ^1^H and ^13^C compound **5h**

**Figure S20** ^1^H and ^13^C compound **5i**

**Figure S21** ^1^H and ^13^C compound **5j**

**Figure S22** ^1^H and ^13^C compound **5k**

1. **Material and methods**

Unless stated otherwise, reagents were obtained from commercial sources such as Sigma-Aldrich Company (Chemie GmbH, Taufkirchen, Germany) and used without further purification. New compounds were fully characterized. Thin-layer chromatography (TLC) was performed on Merck Silica Gel 60 F254, 20 x 20 cm plates, and visualized using a 254 nm UV lamp. A standard rotary evaporator was used for vacuumed removal of the solvents. ^1^H- and ^13^C- NMR spectra were recorded in CDCl_3_ and DMSO-*d*_6_ on a JEOL spectrometer (JEOL, Tokyo, Japan) (400 or 500 MHz) and referenced to residual solvent signals (*δ* 2.50 and 7.26 *δ*, respectively). ^1^H-NMR data were reported as chemical shift (*δ* ppm) and the multiplicity of the signals are indicated as s = singlet, d = doublet, t = triplet, q = quartet, m= multiplet, and dd = doublet of doublets. Coupling constants (*J*) are given in Hz and reported to the nearest 0.1 Hz. ^13^C-NMR was reported as chemical shifts (*δ* ppm). Infrared spectra were recorded on a Thermo Scientific Nicolet iS10 FT-IR spectrometer (Thermo Fisher Scientific, Waltham, MA, USA).

**NMR CHARTS**

**
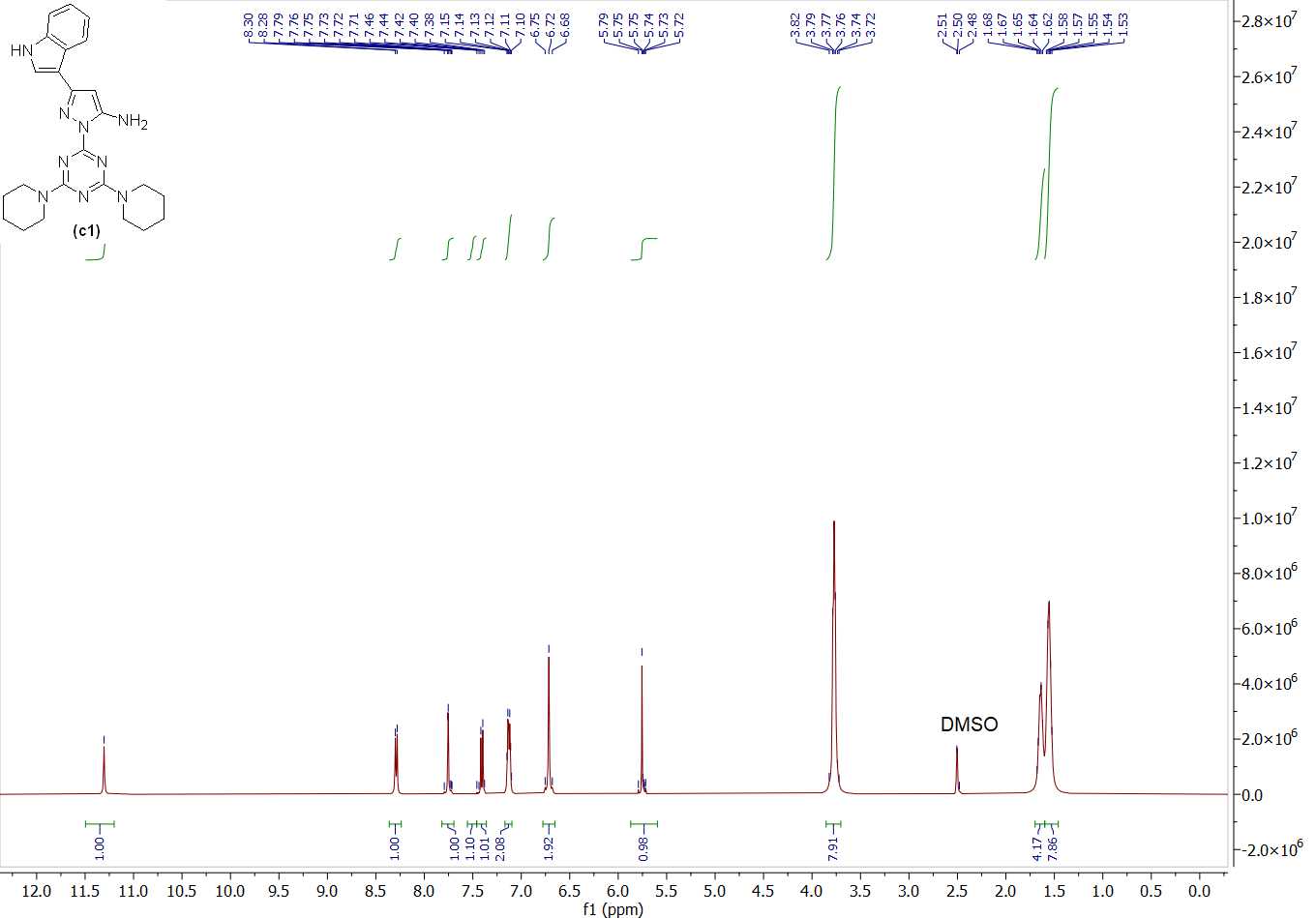
**

**
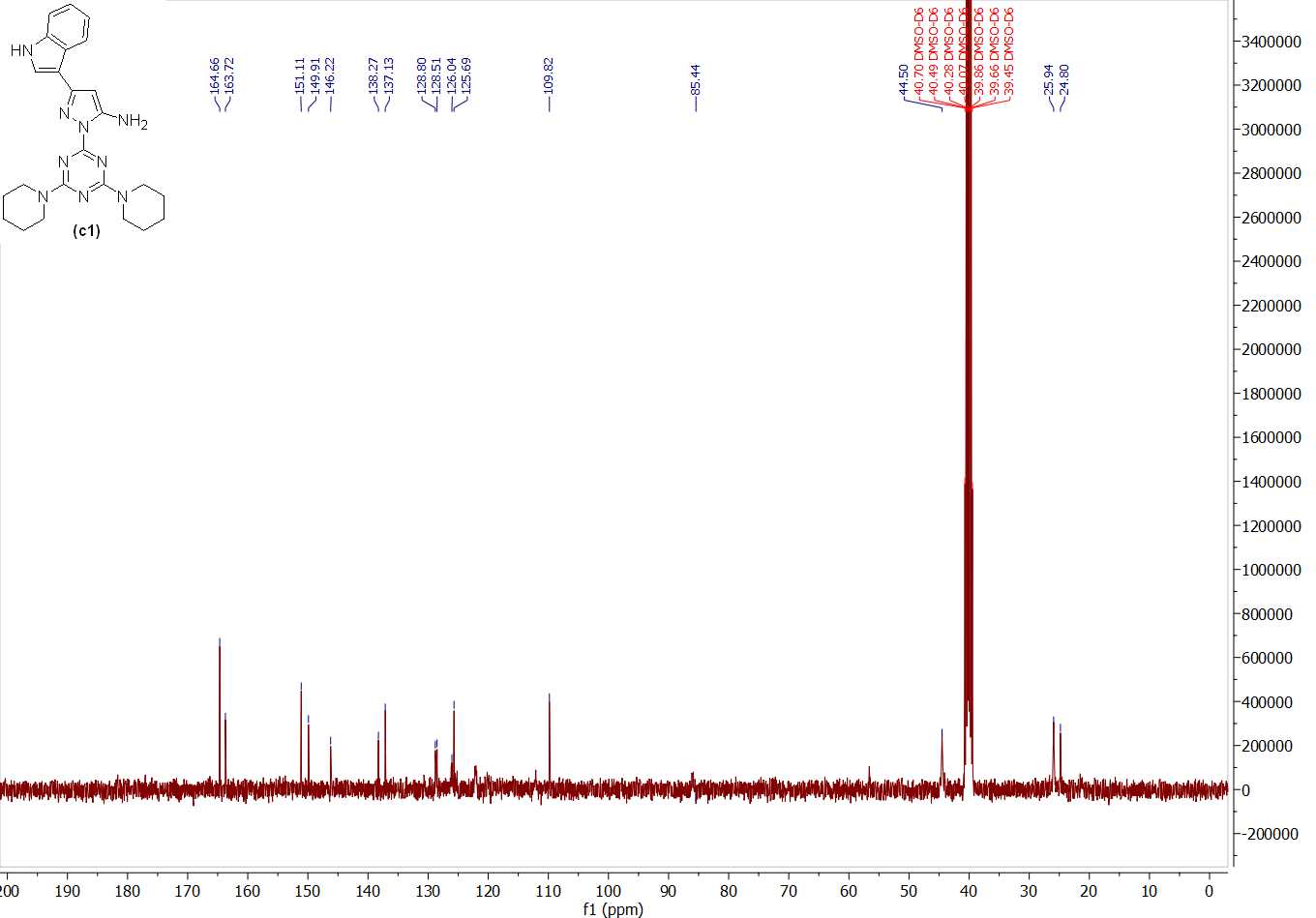
**

**Figure S1:** ^1^H-NMR and ^13^C-NMR of **3a**

**
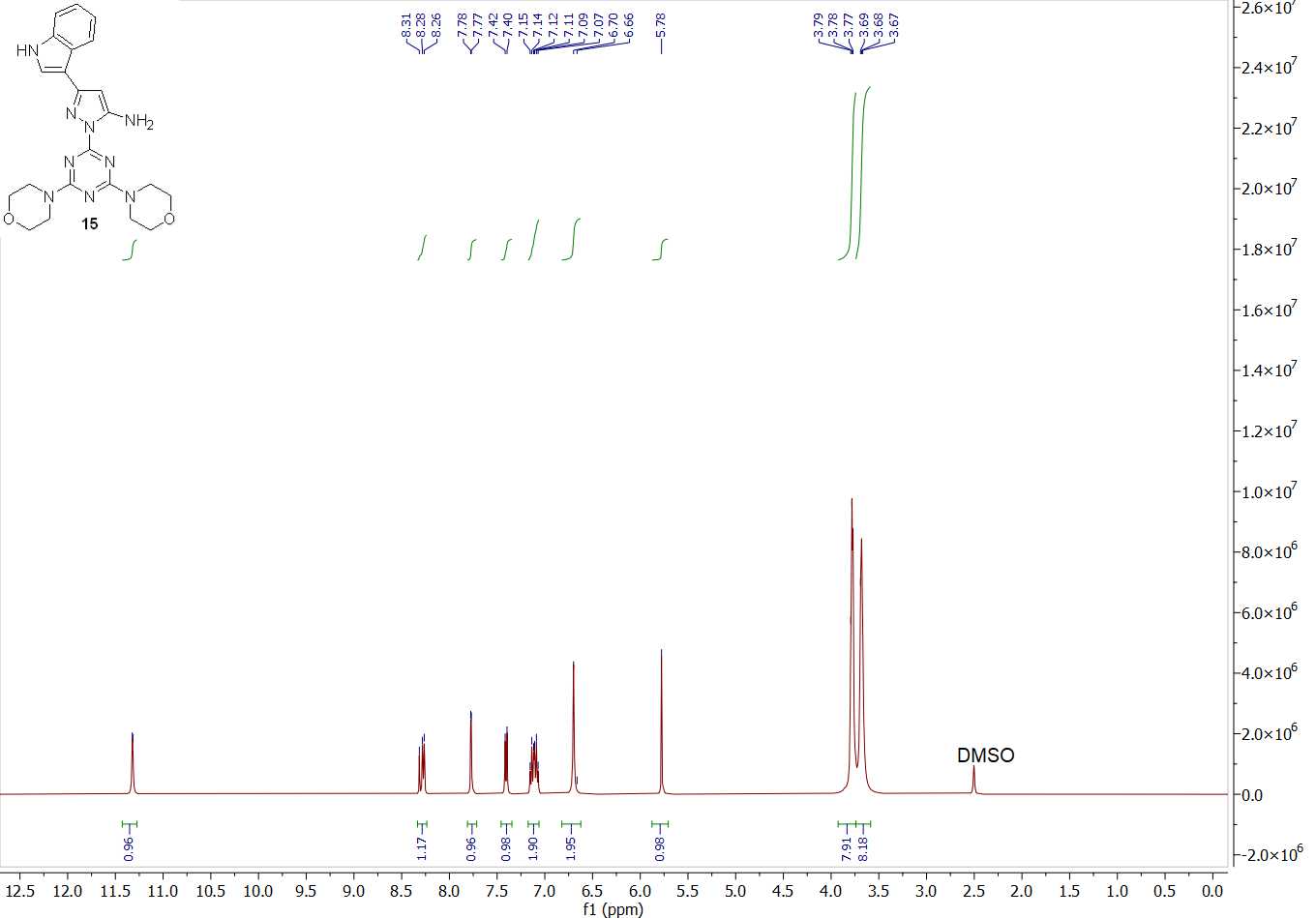
**

**
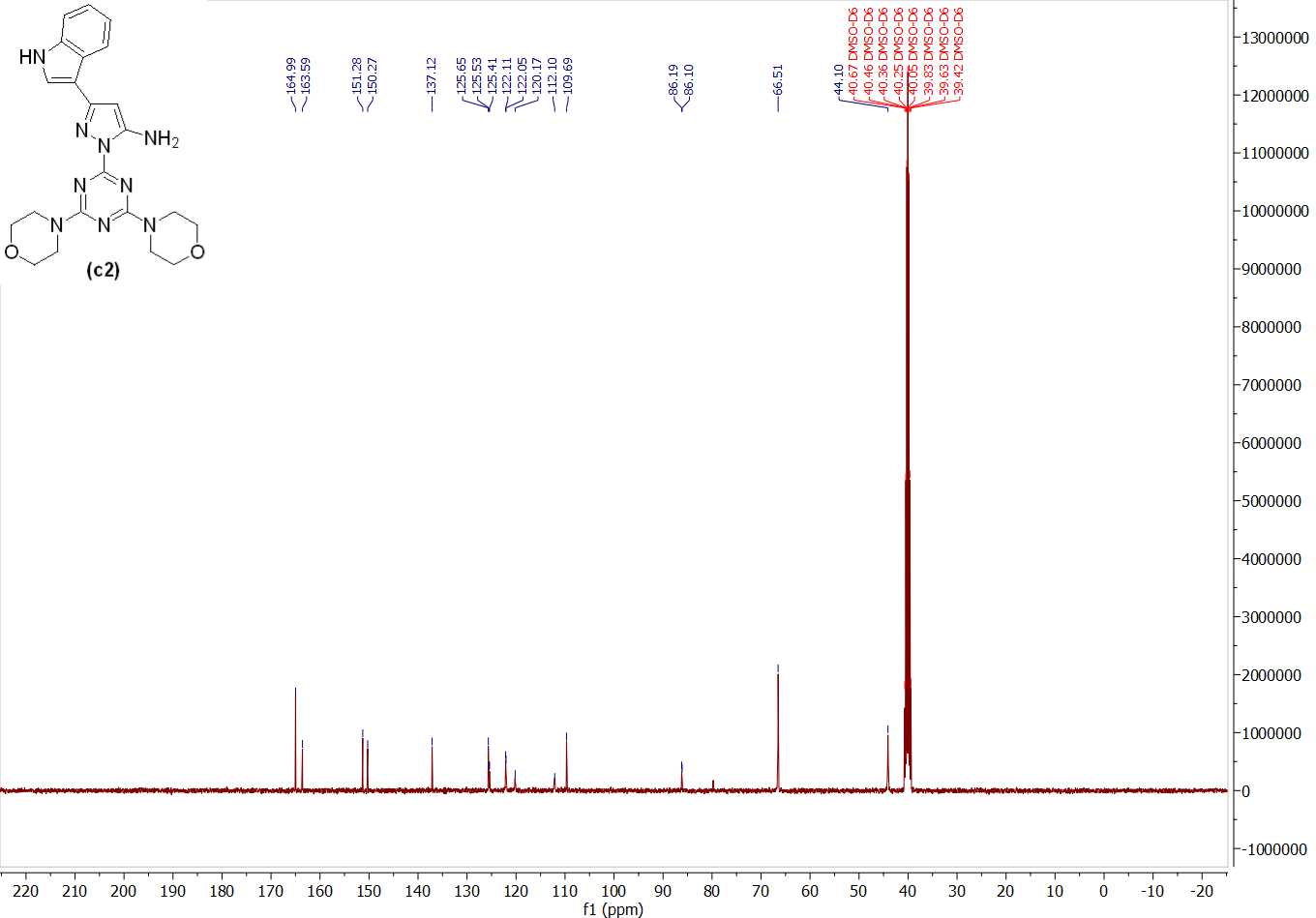
**

**Figure S2:** ^1^H-NMR and ^13^C-NMR of **3b**


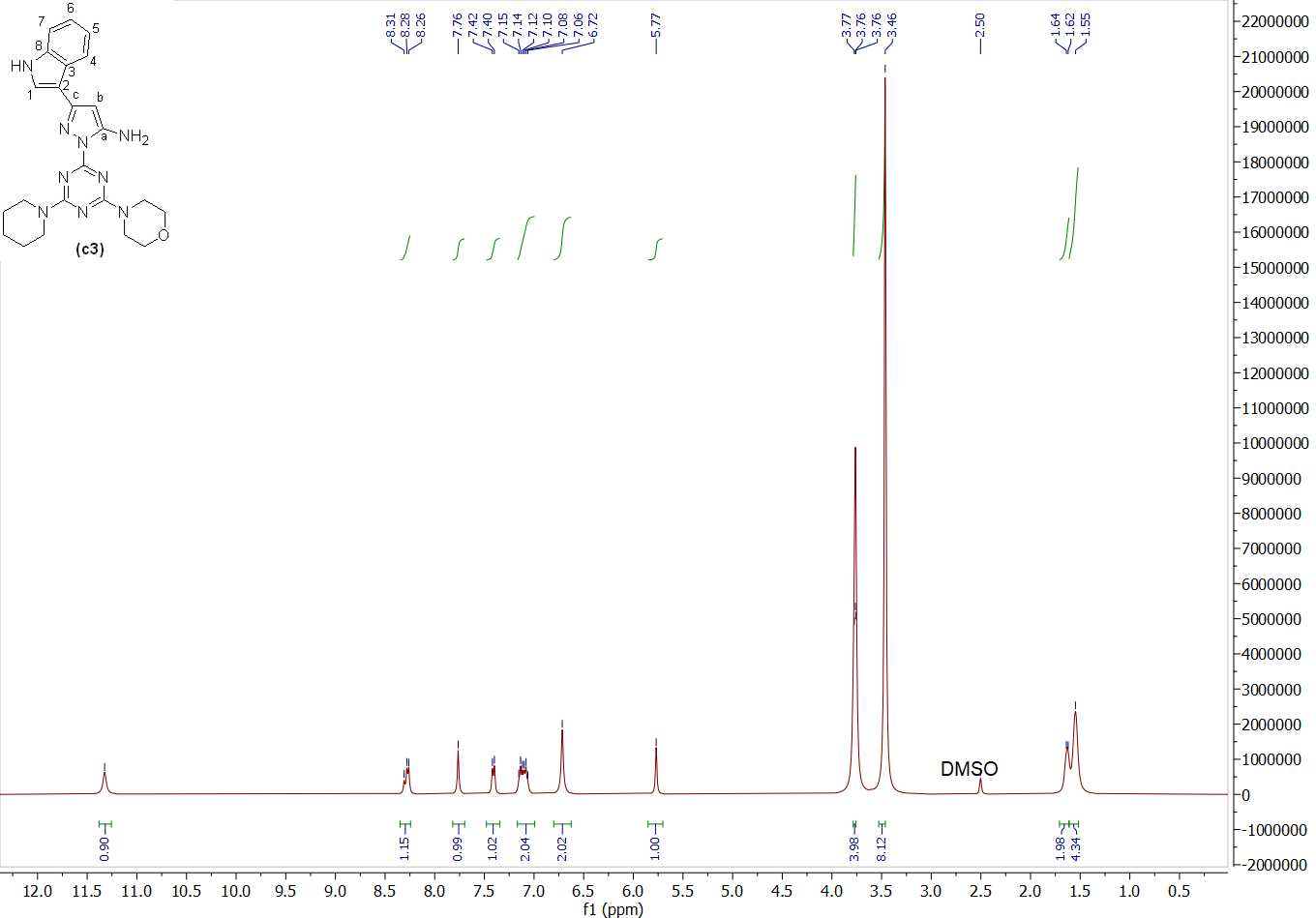


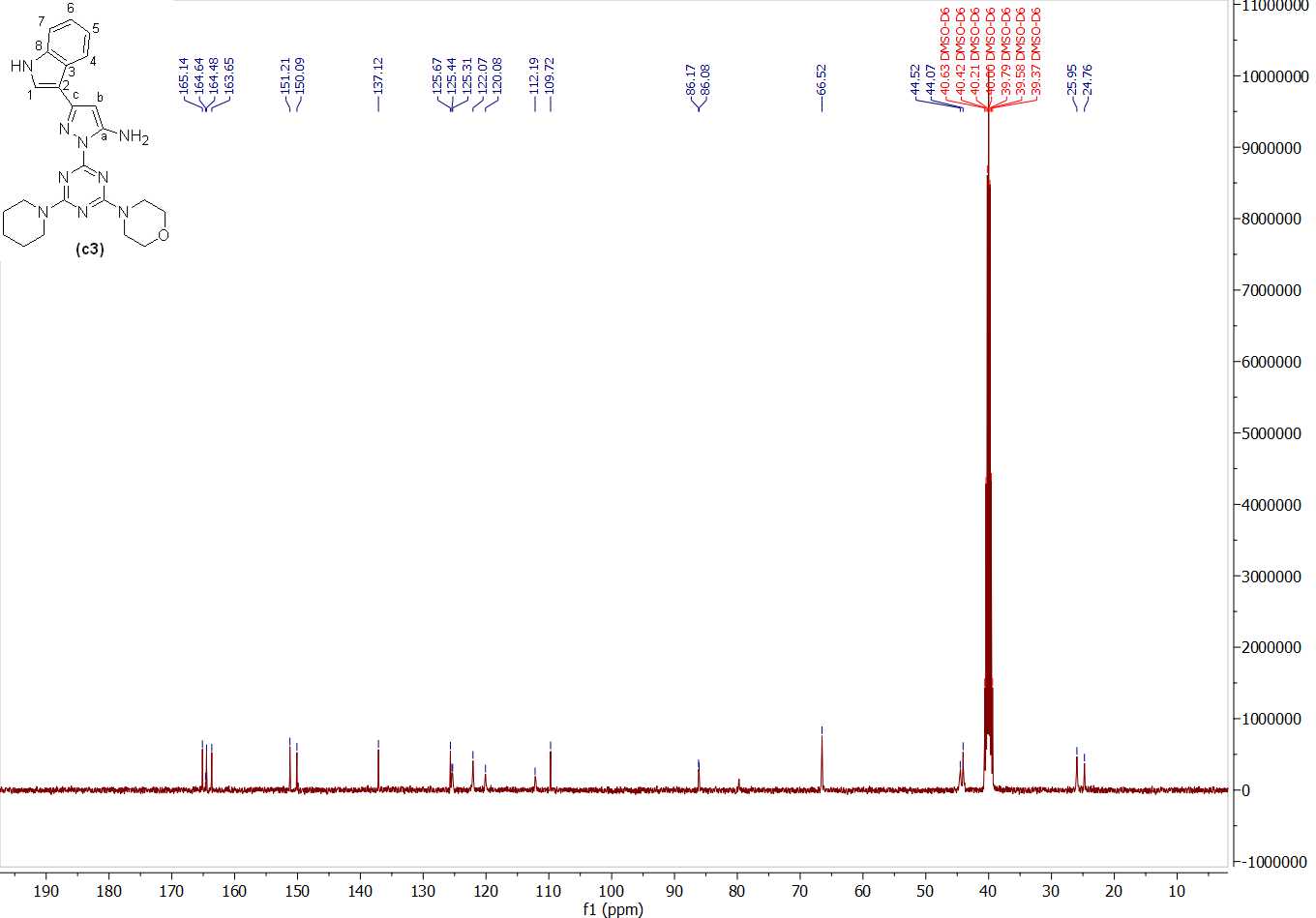


**Figure S3:** ^1^H-NMR and ^13^C-NMR of **3c**

**
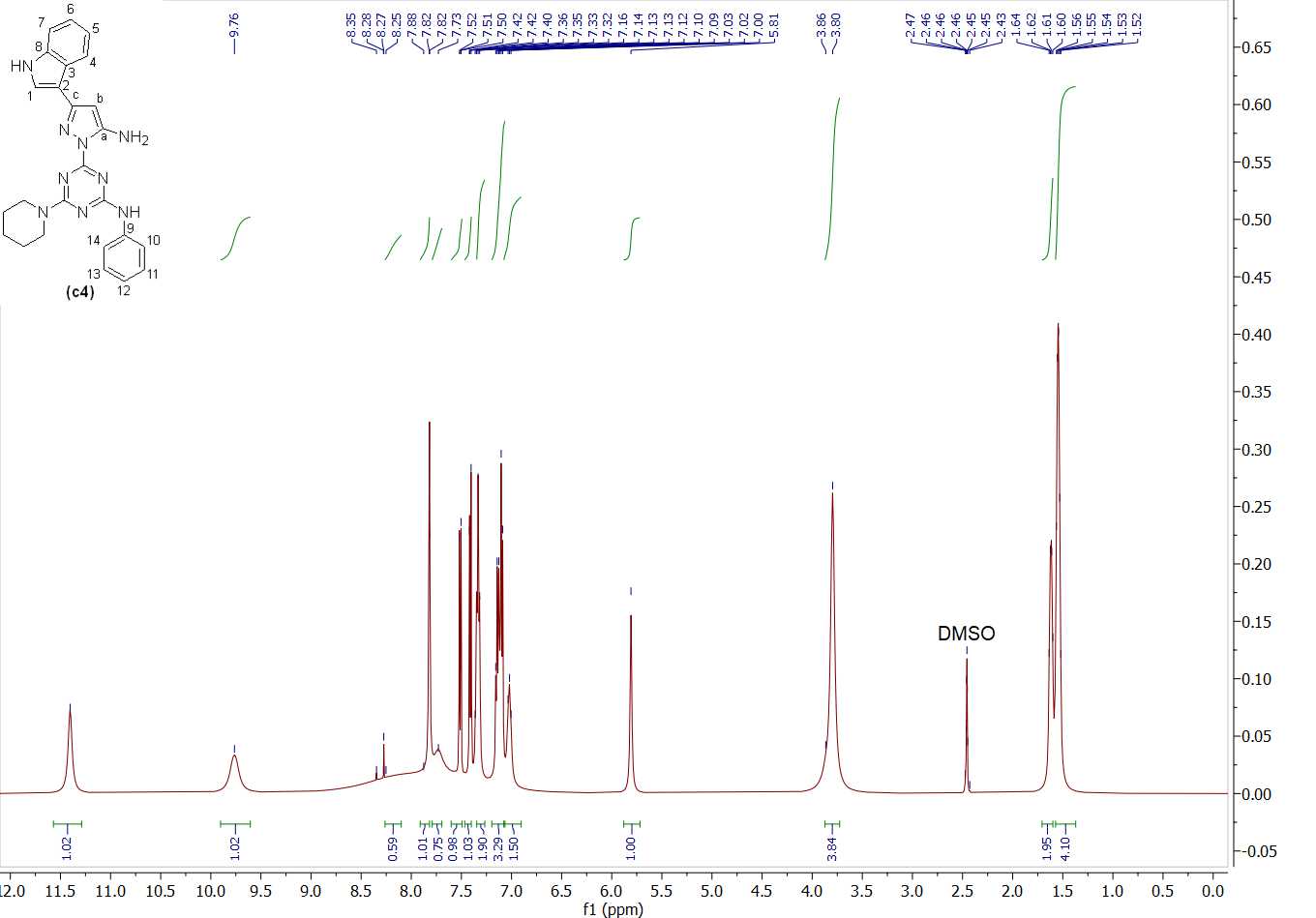
**


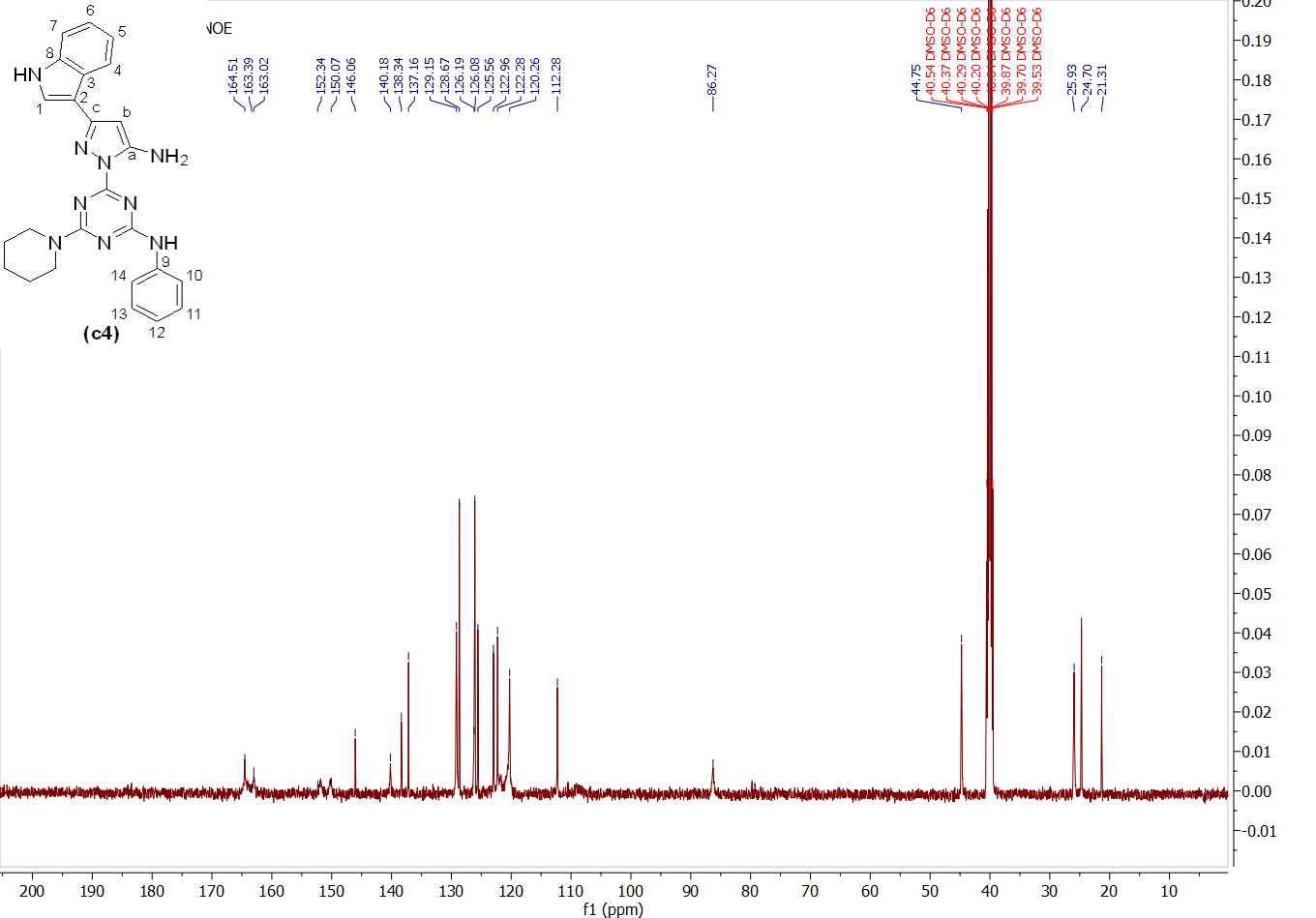


**Figure S4:** ^1^H-NMR and ^13^C-NMR of **3d**


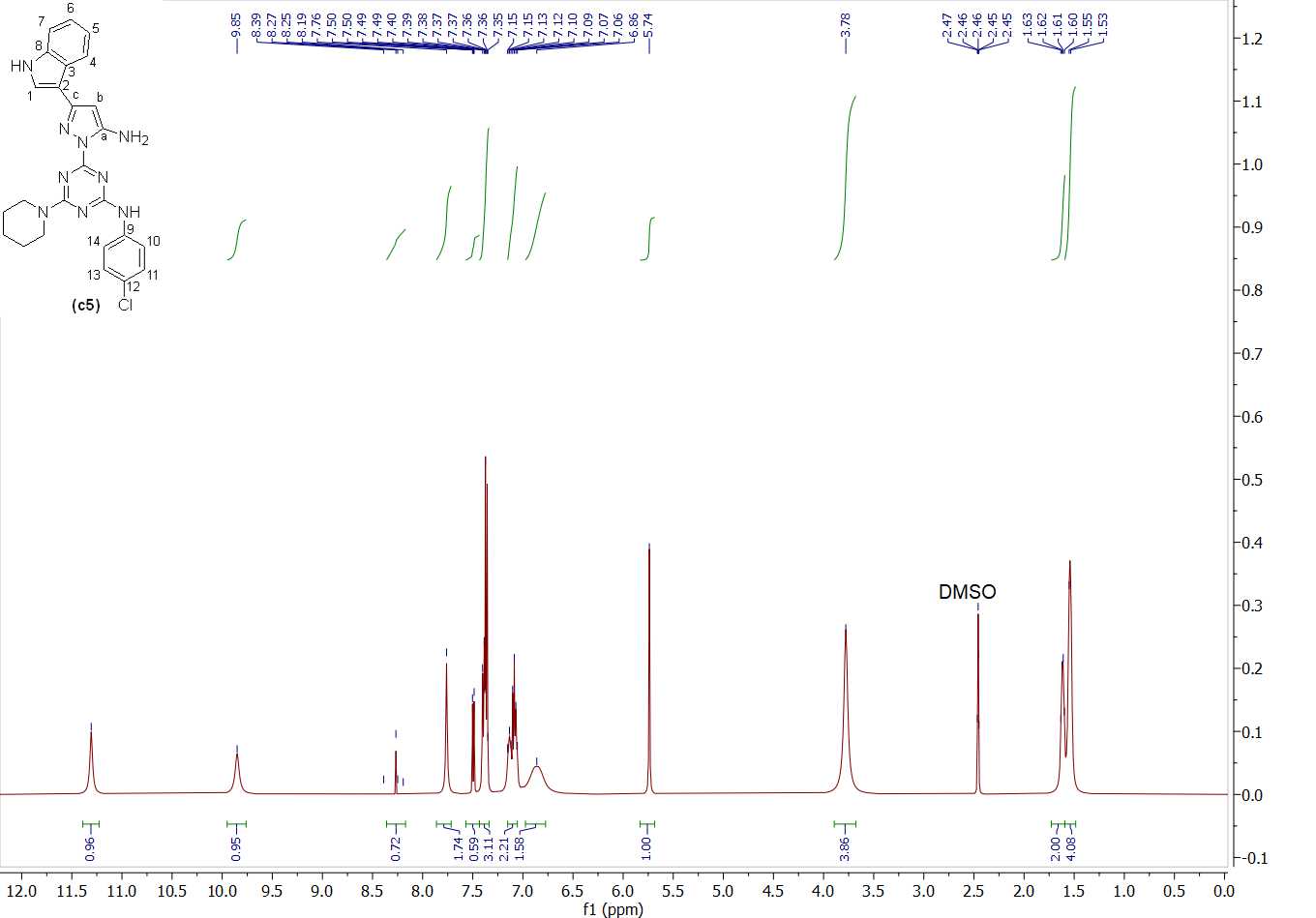


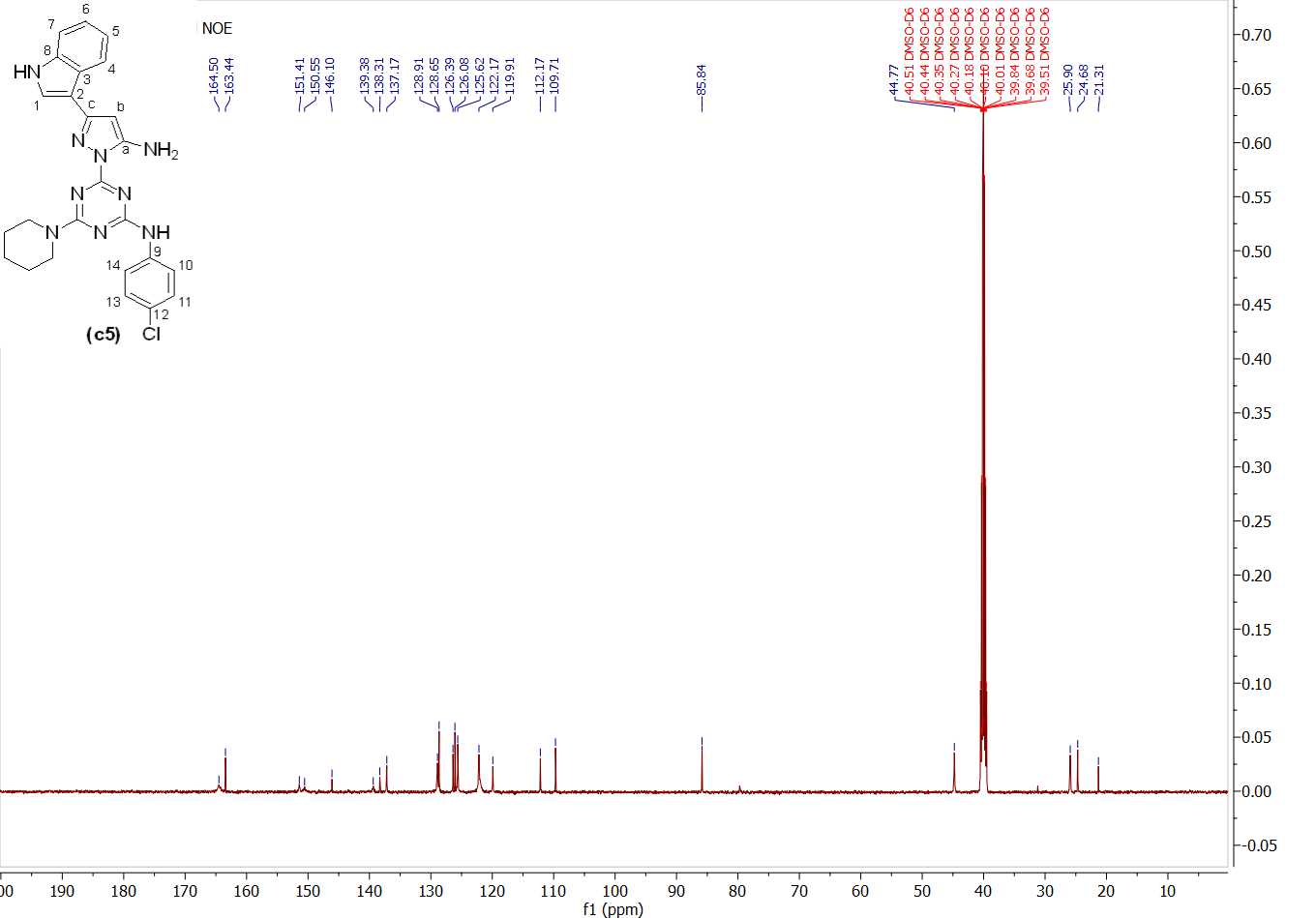


**Figure S5:** ^1^H-NMR and ^13^C-NMR of **3e**


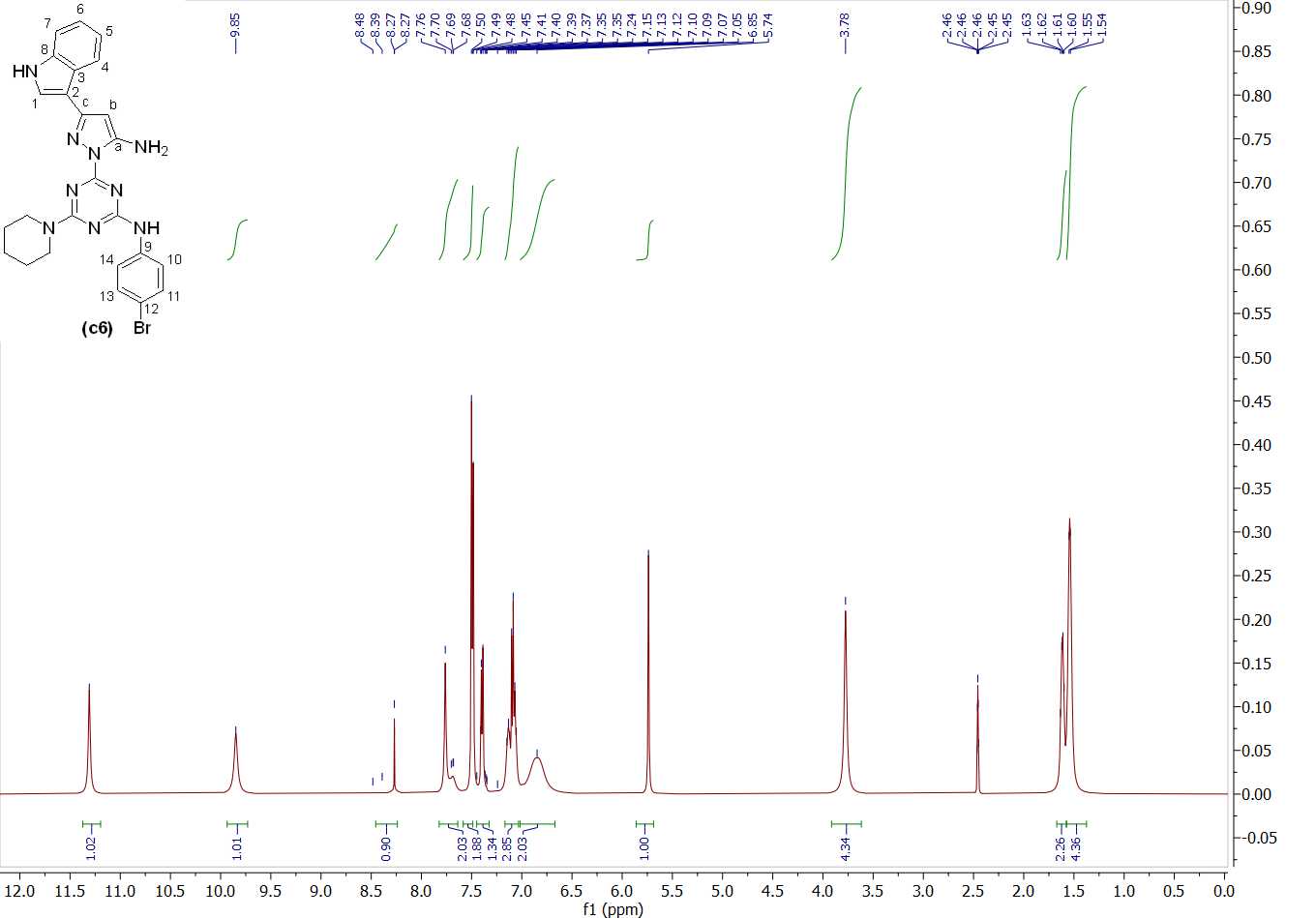


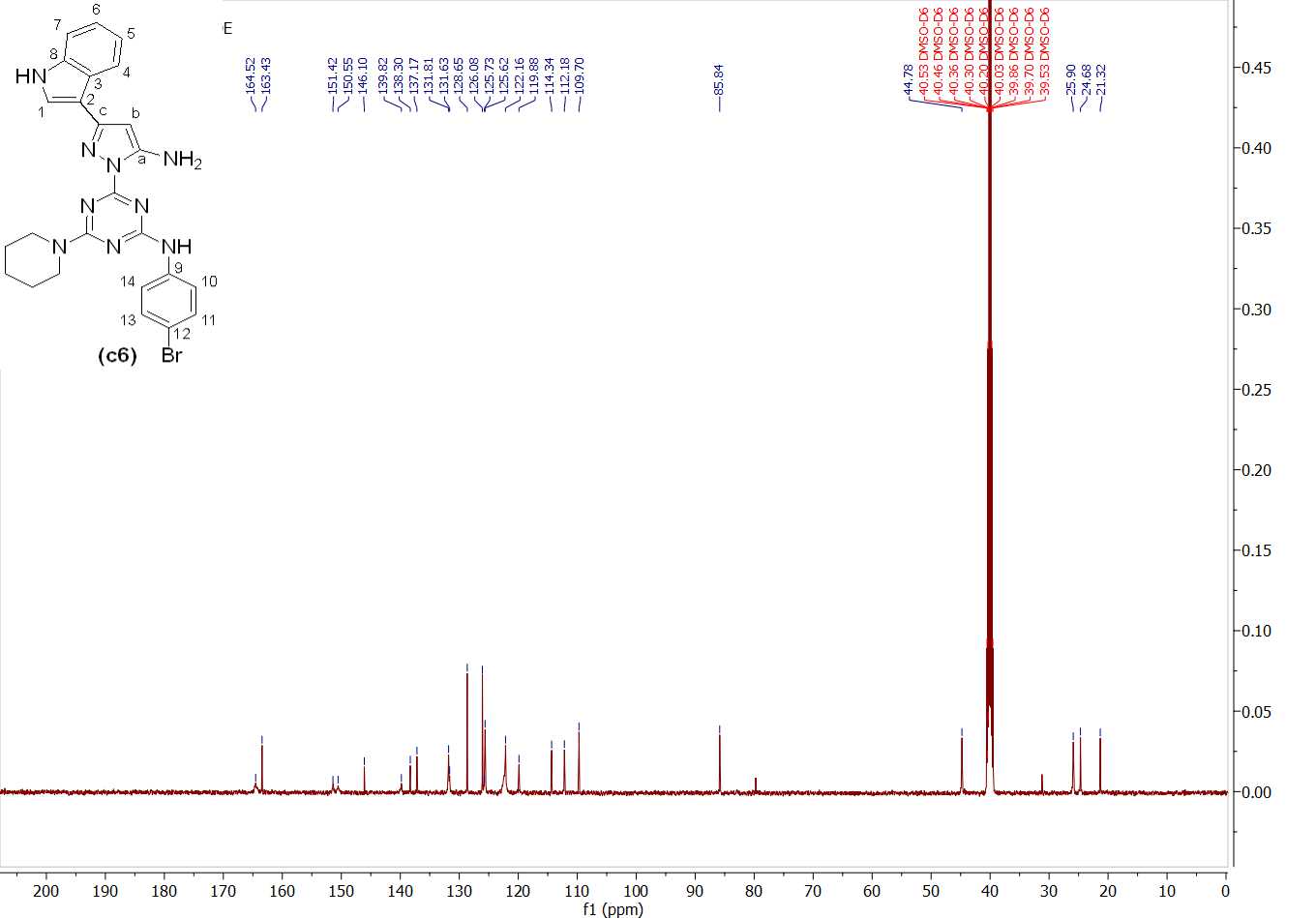


**Figure S6:** ^1^H-NMR and ^13^C-NMR of **3f**


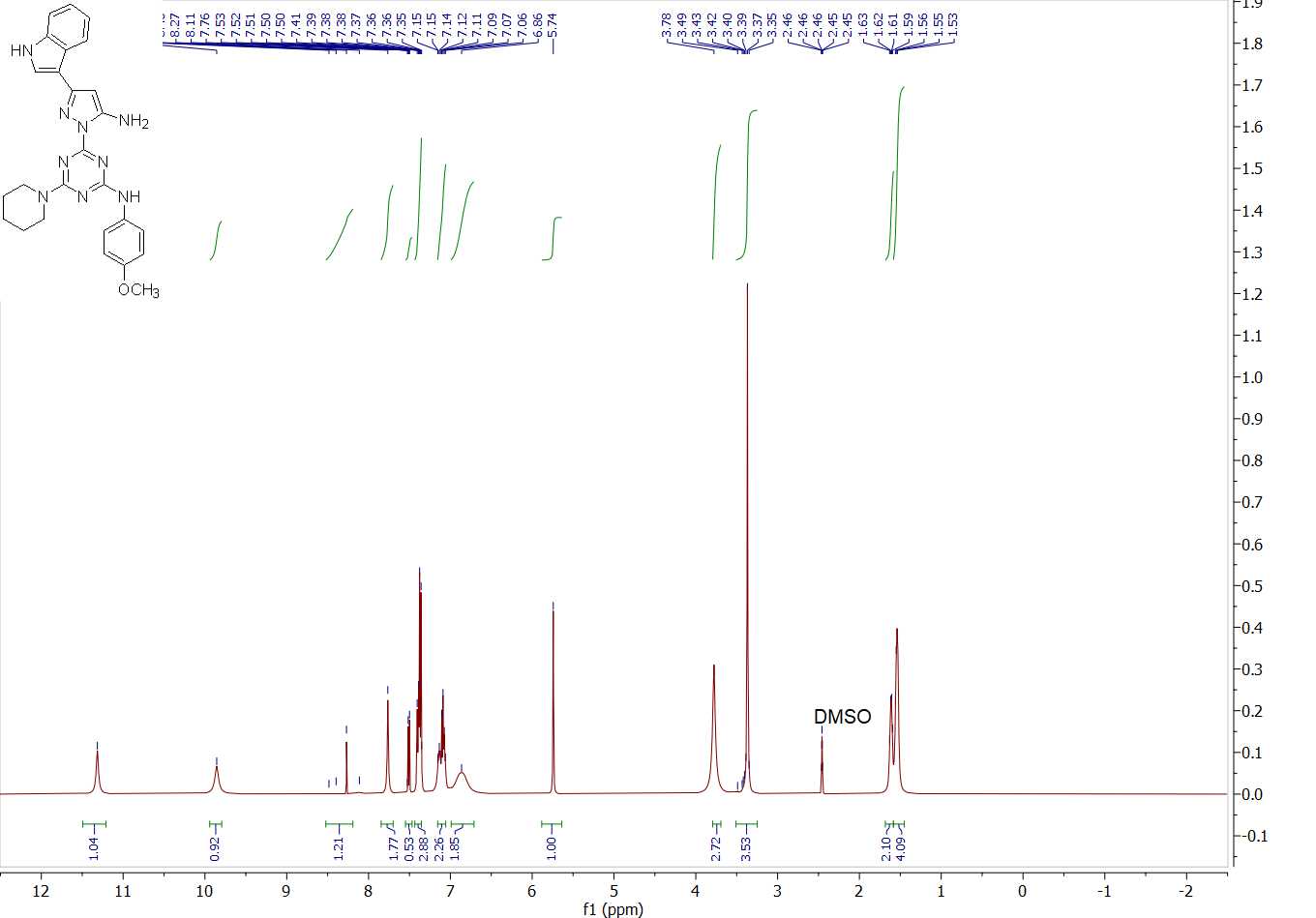


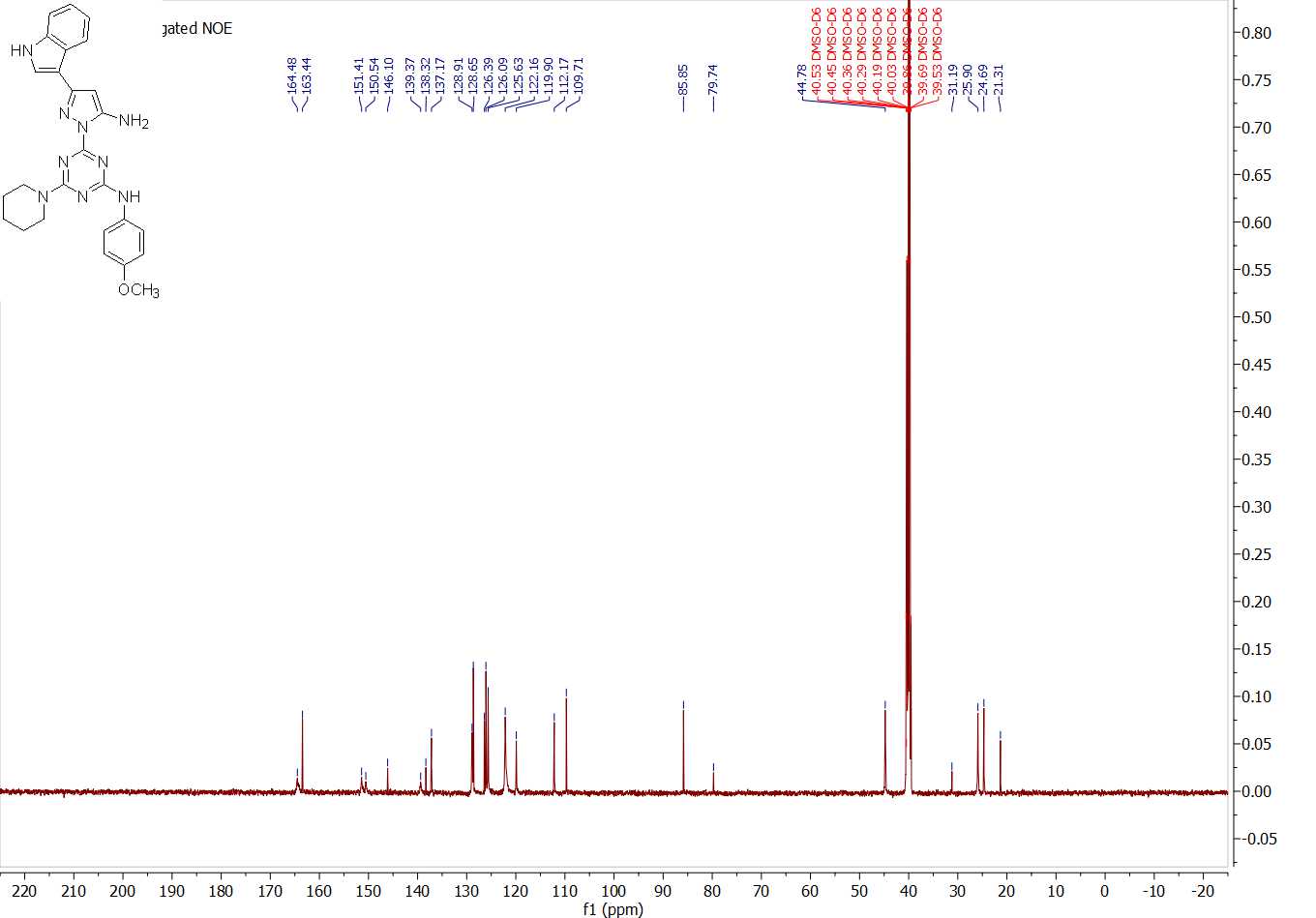


**Figure S7:** ^1^H-NMR and ^13^C-NMR of **3g**


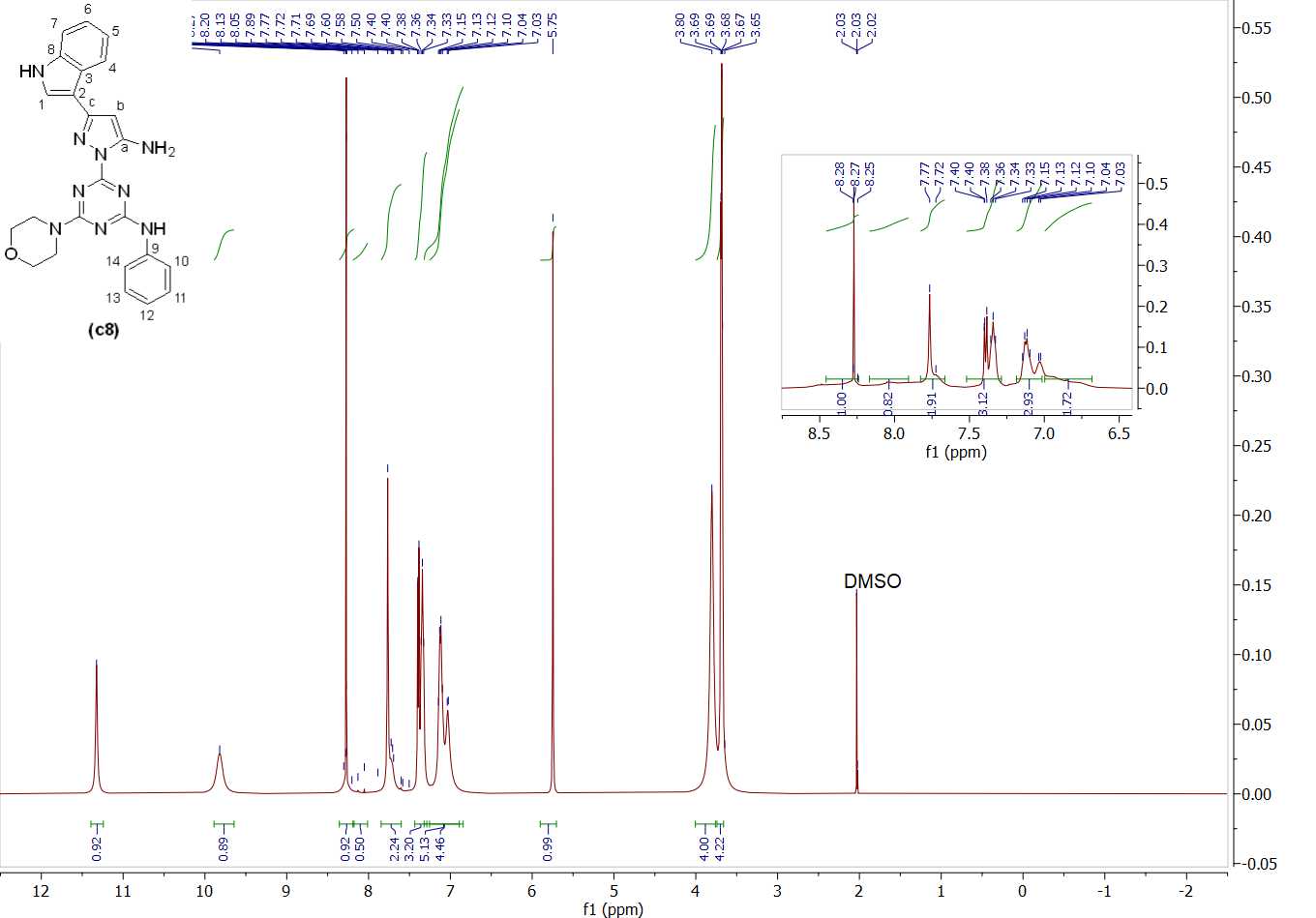


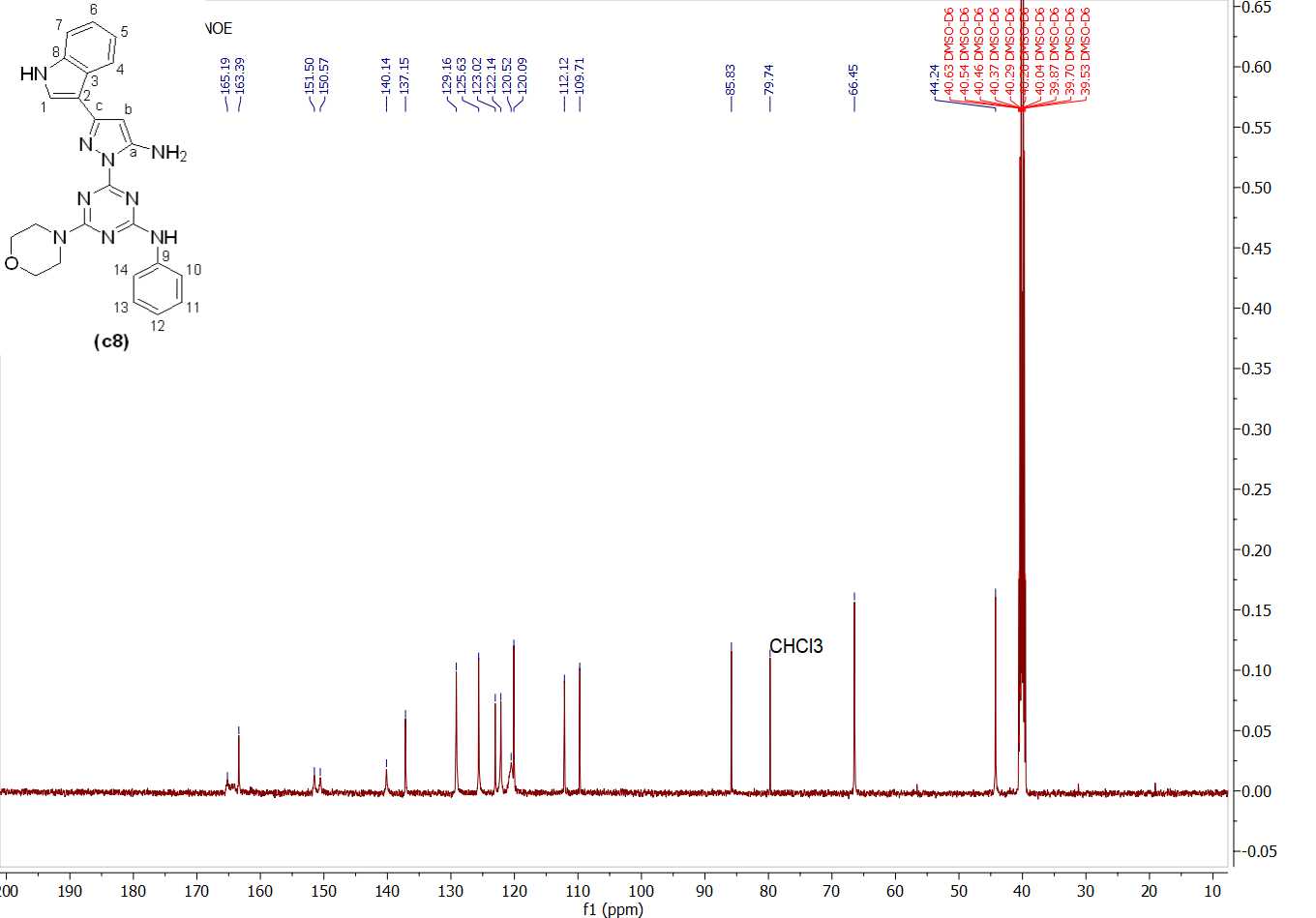


**Figure S8:** ^1^H-NMR and ^13^C-NMR of **3h**


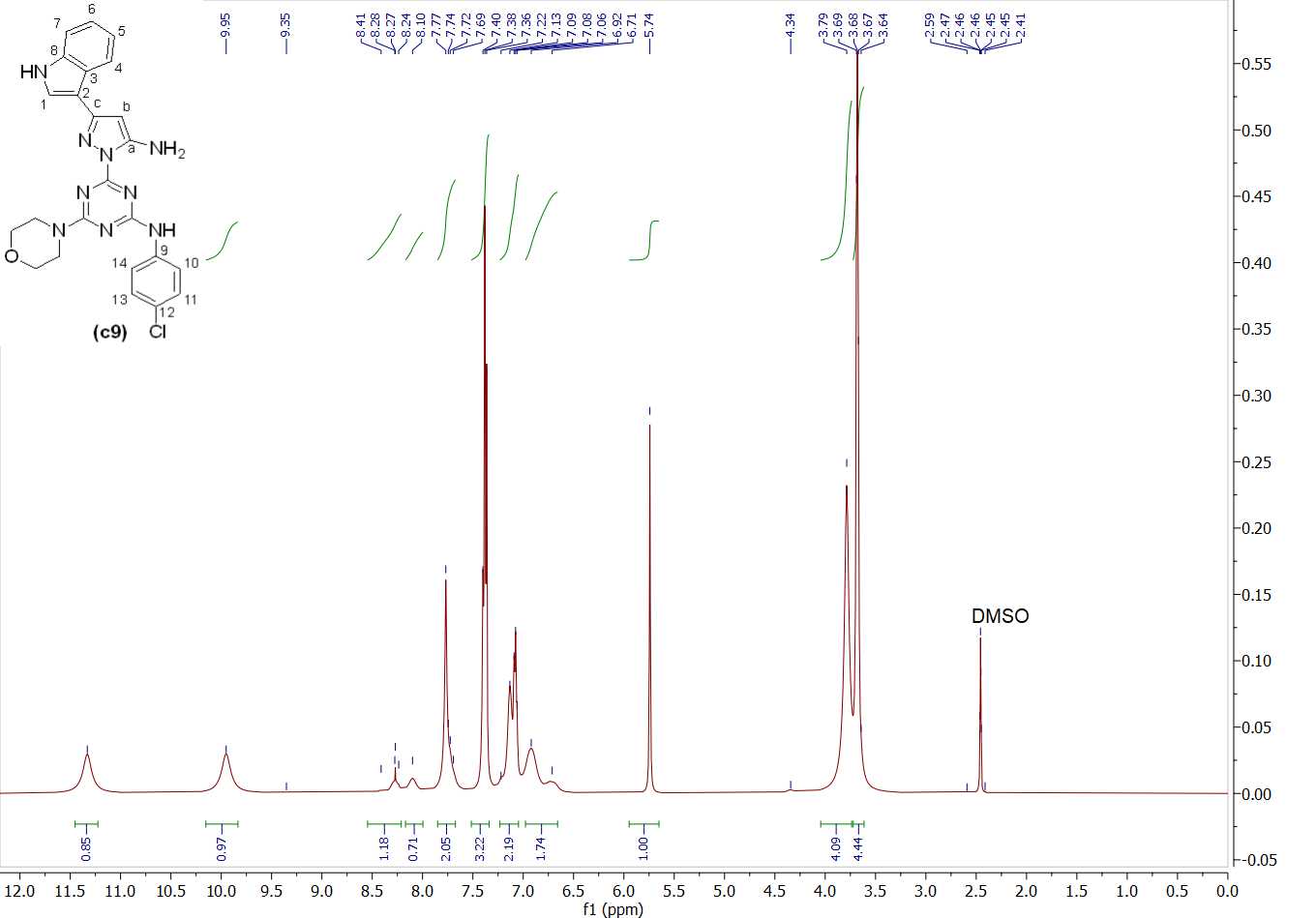


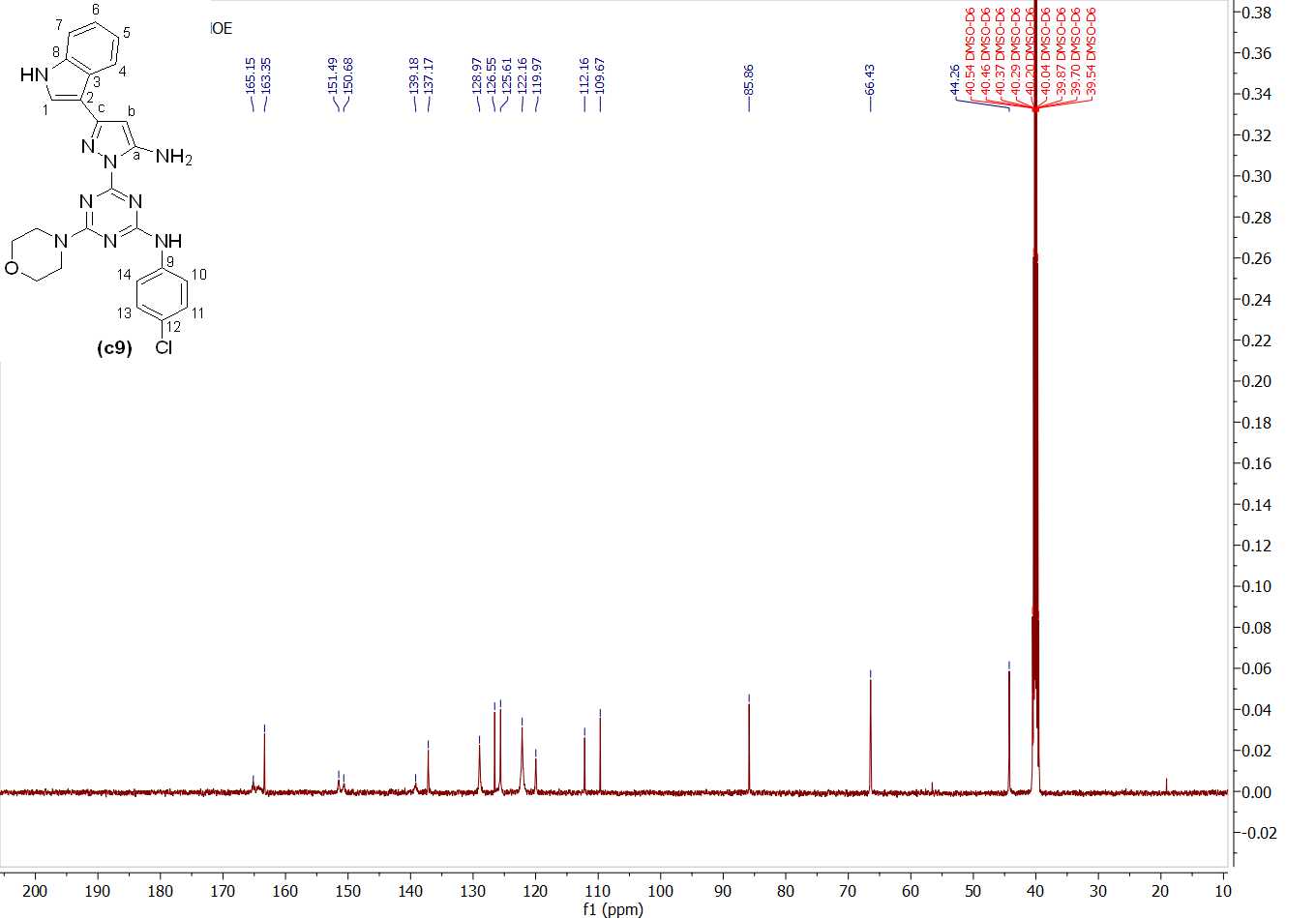


**Figure S9:** ^1^H-NMR and ^13^C-NMR of **3i**


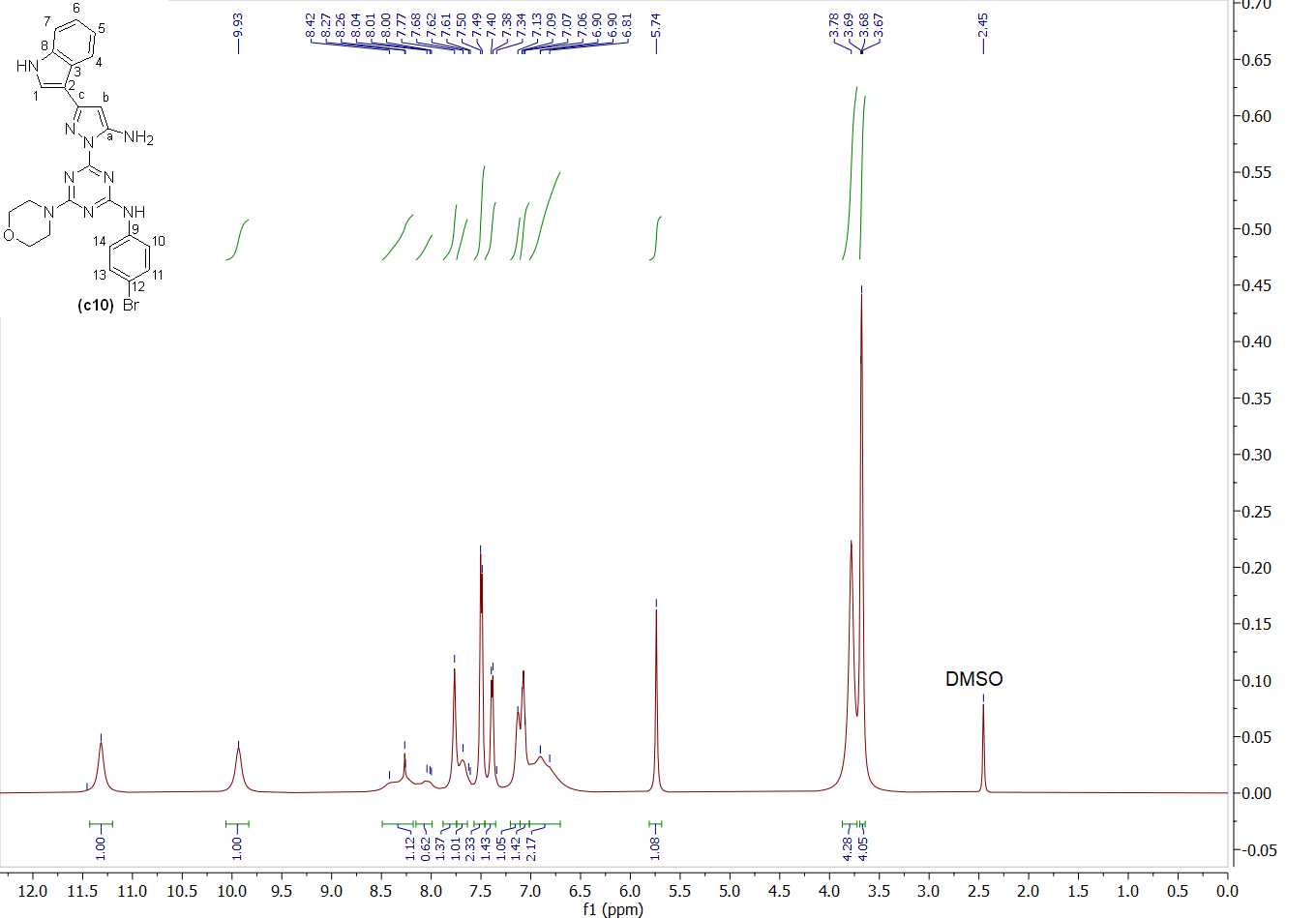


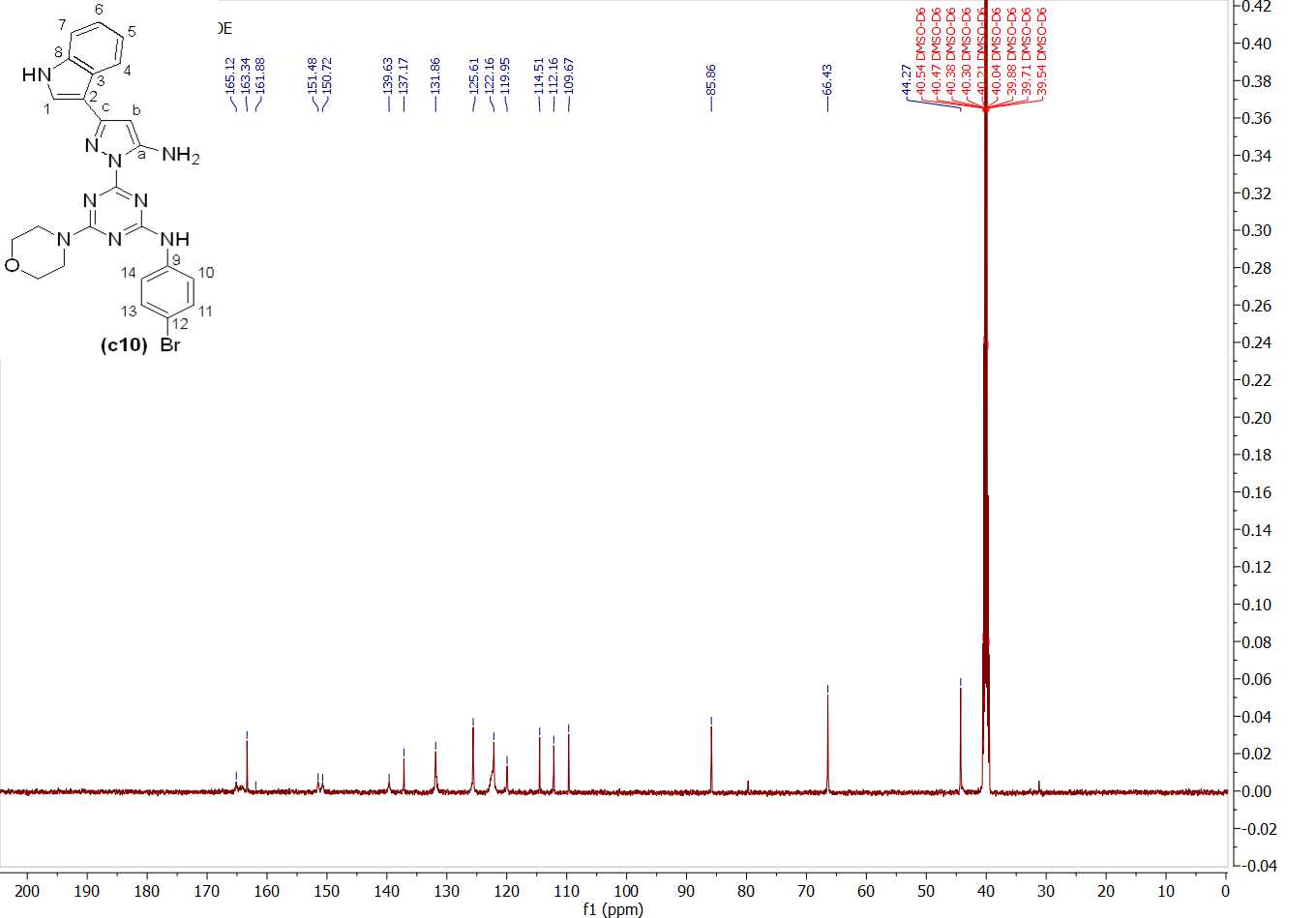


**Figure S10:** ^1^H-NMR and ^13^C-NMR of **3j**


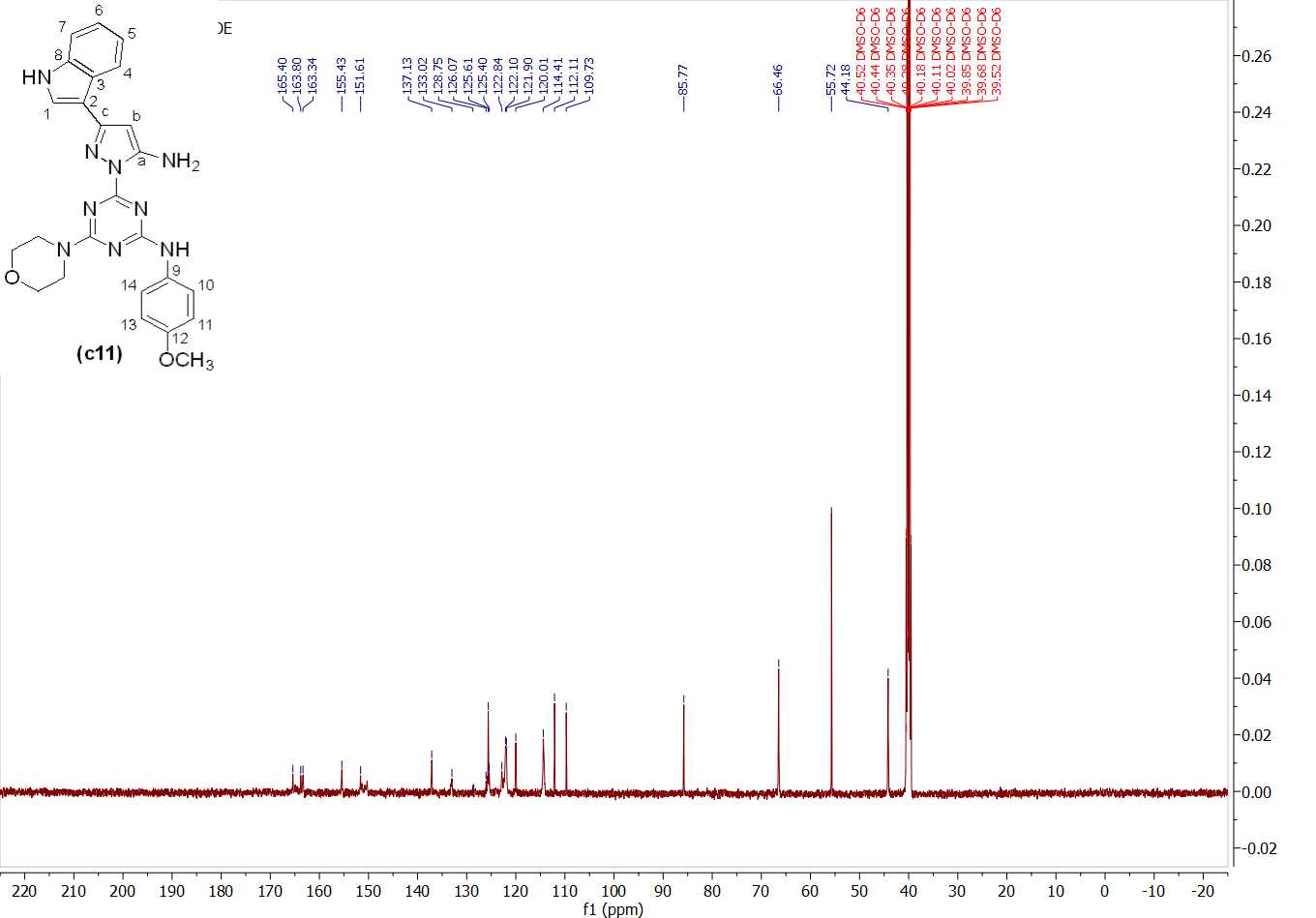


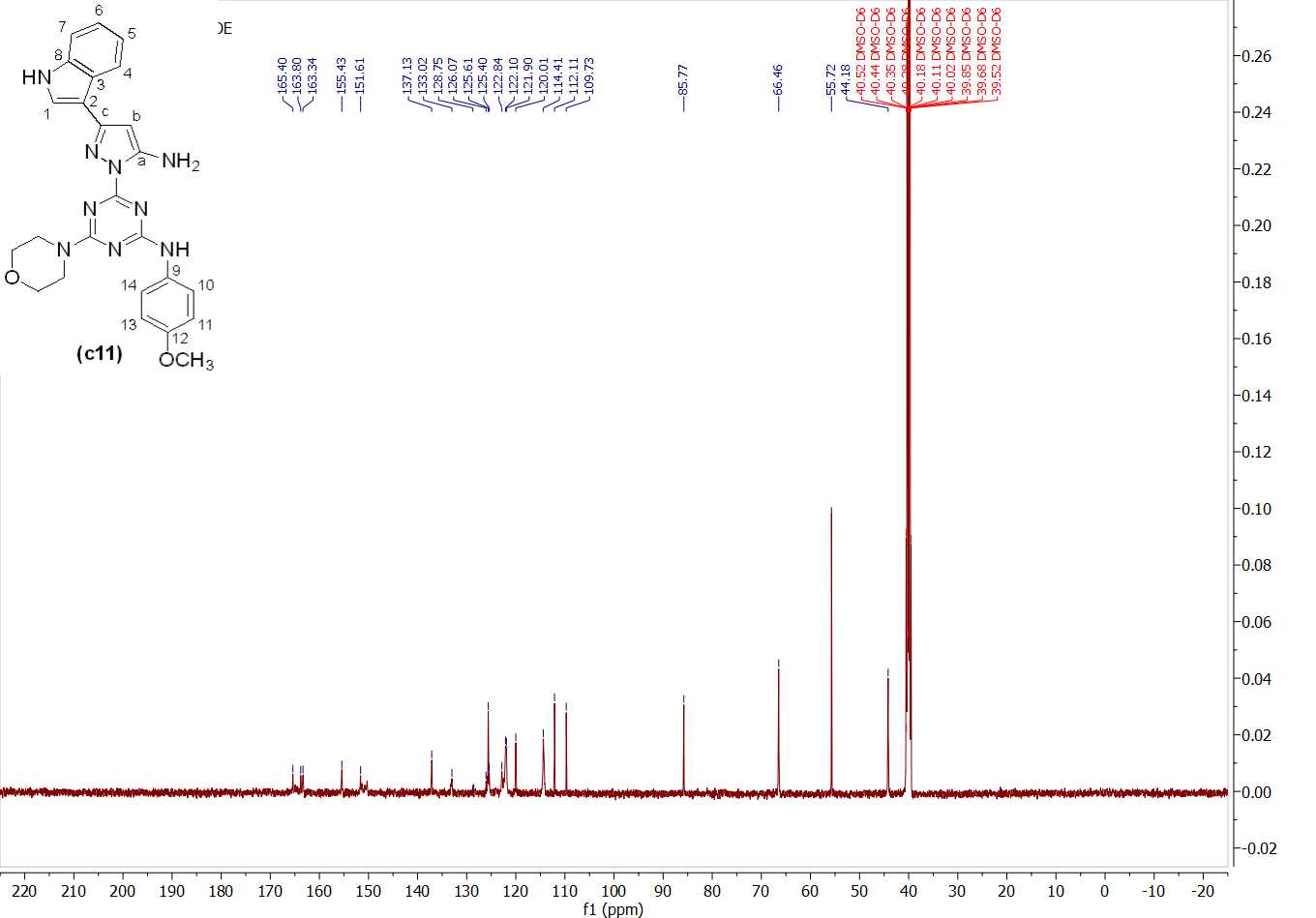


**Figure S11:** ^1^H-NMR and ^13^C-NMR of **3k**


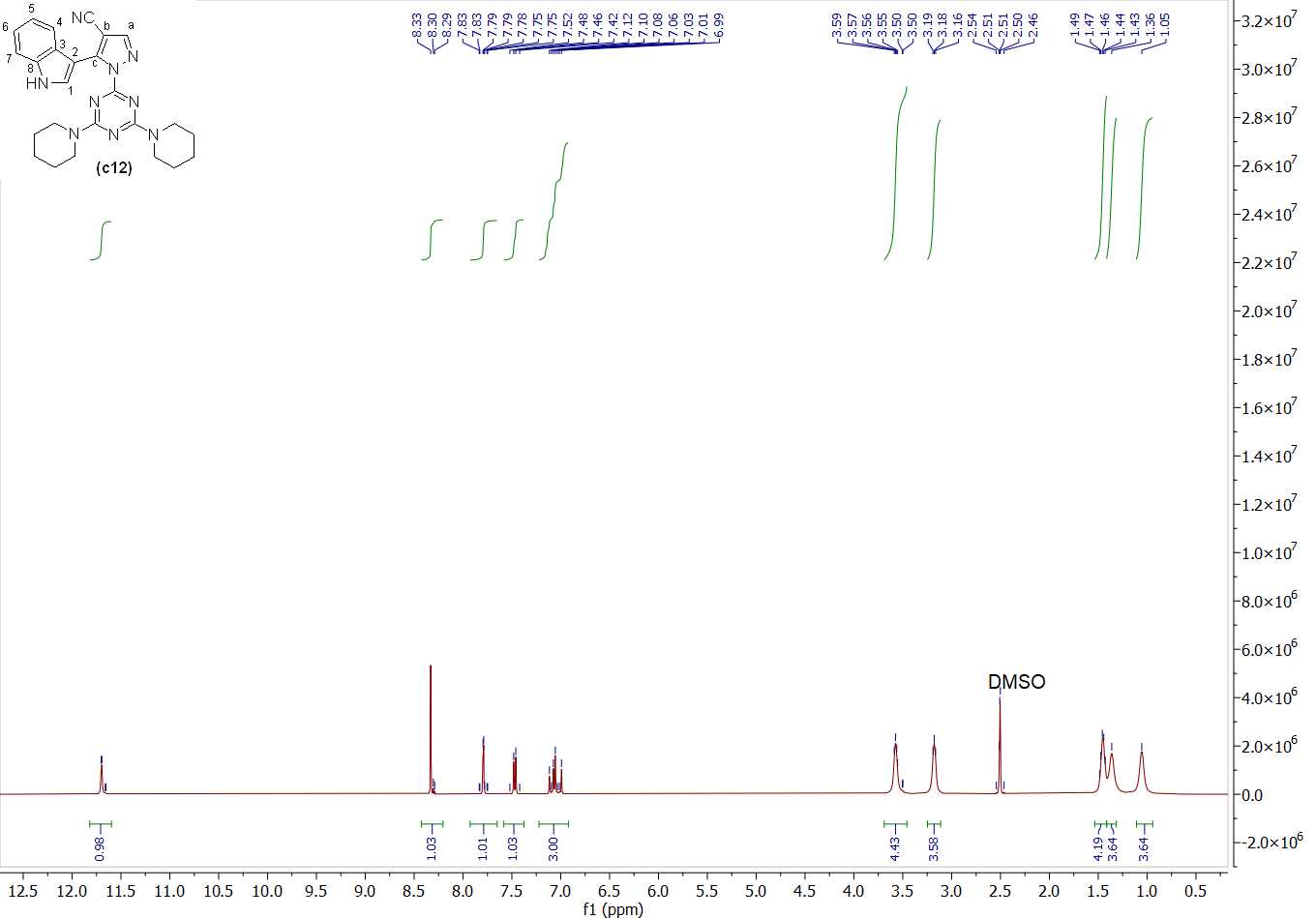


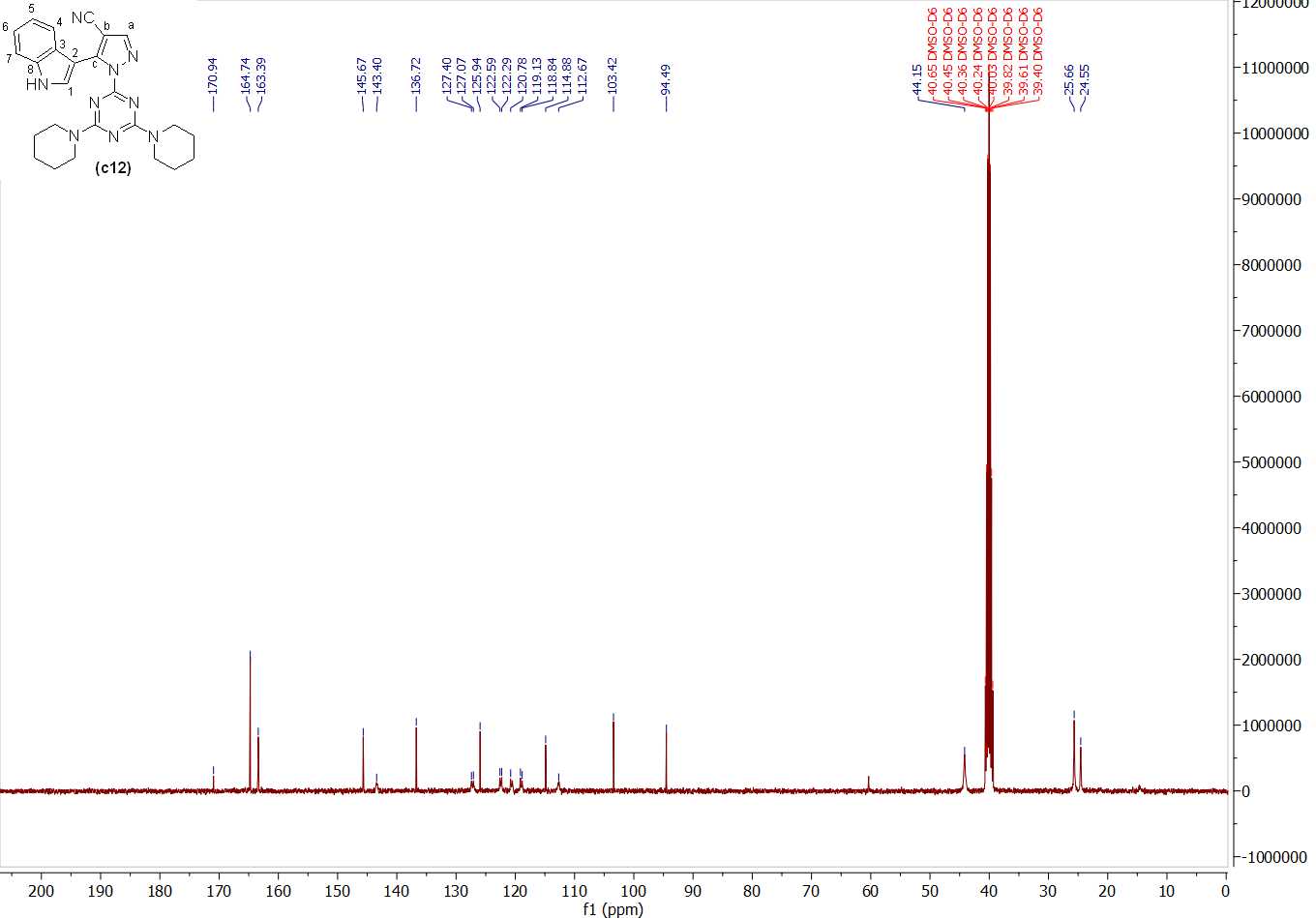


**Figure S12:** ^1^H-NMR and ^13^C-NMR of **5a**


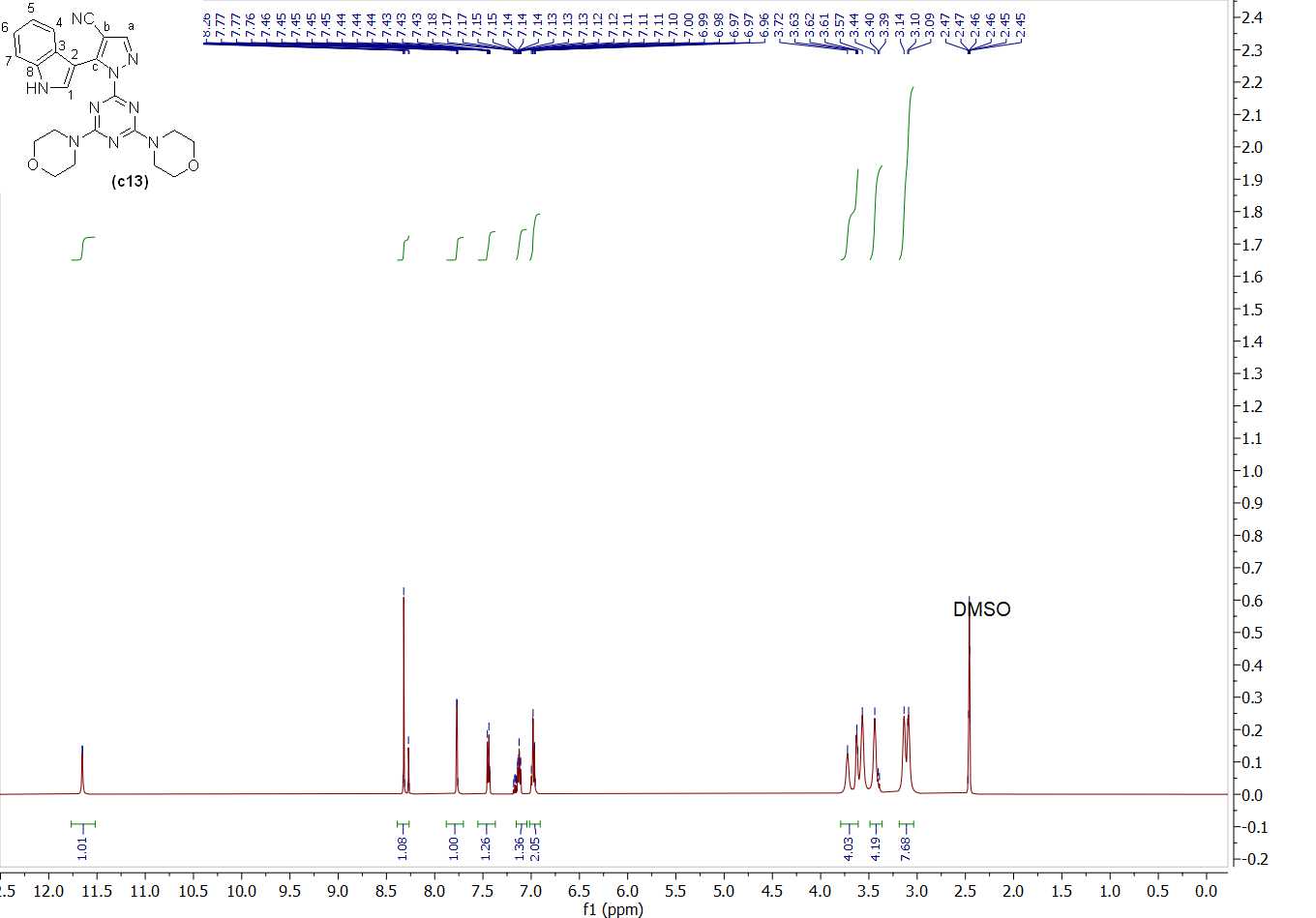


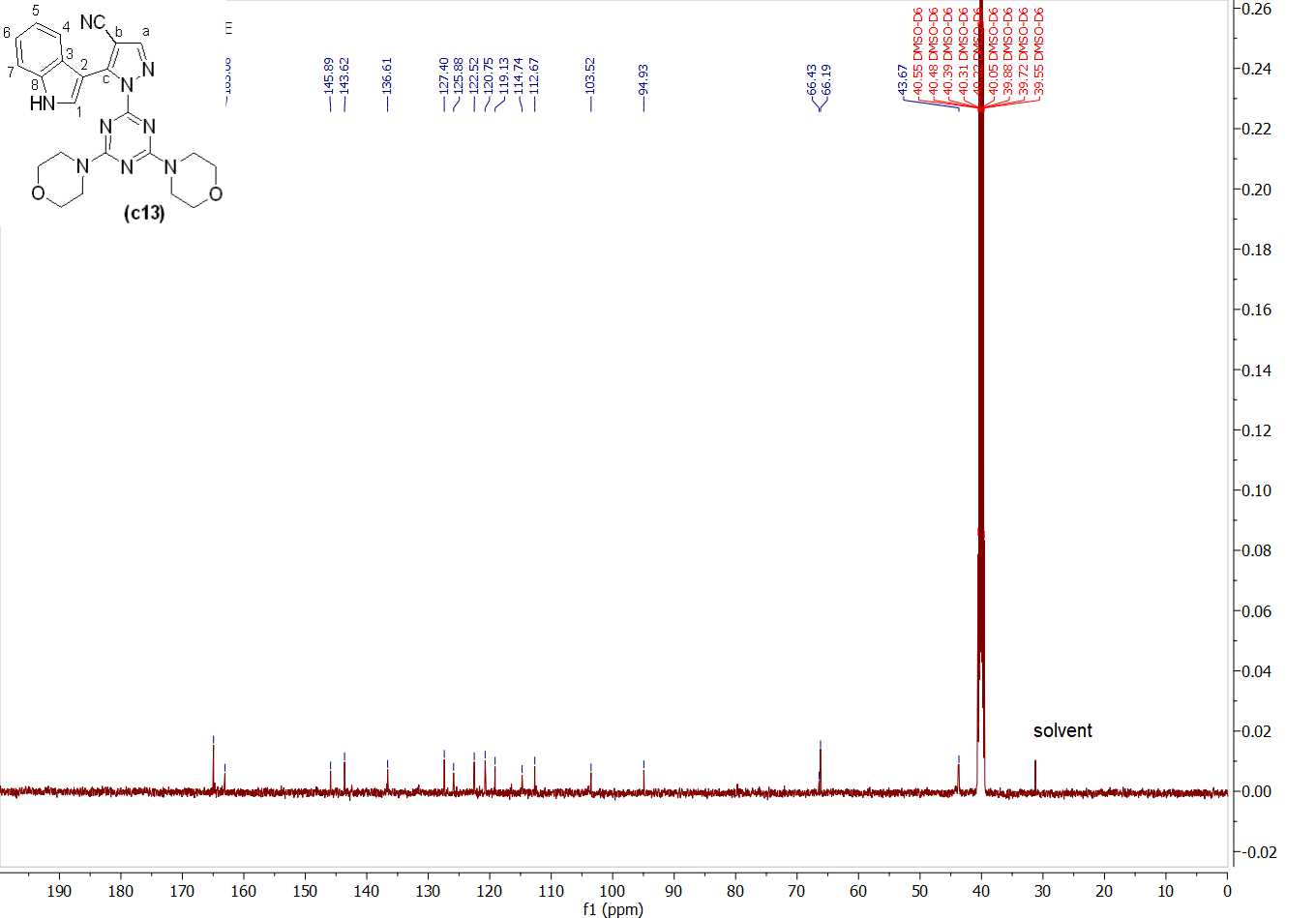


**Figure S13:** ^1^H-NMR and ^13^C-NMR of **5b**


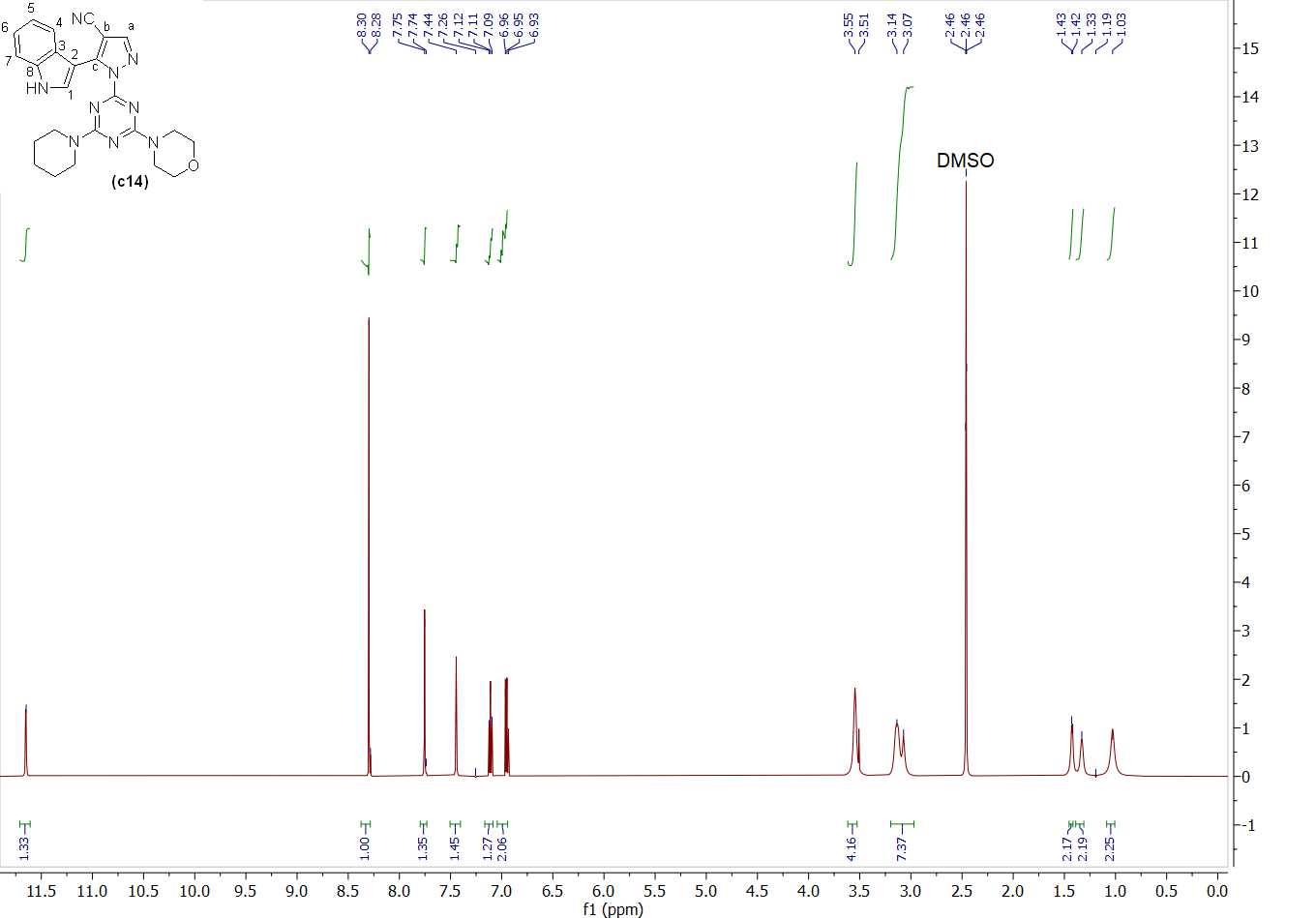


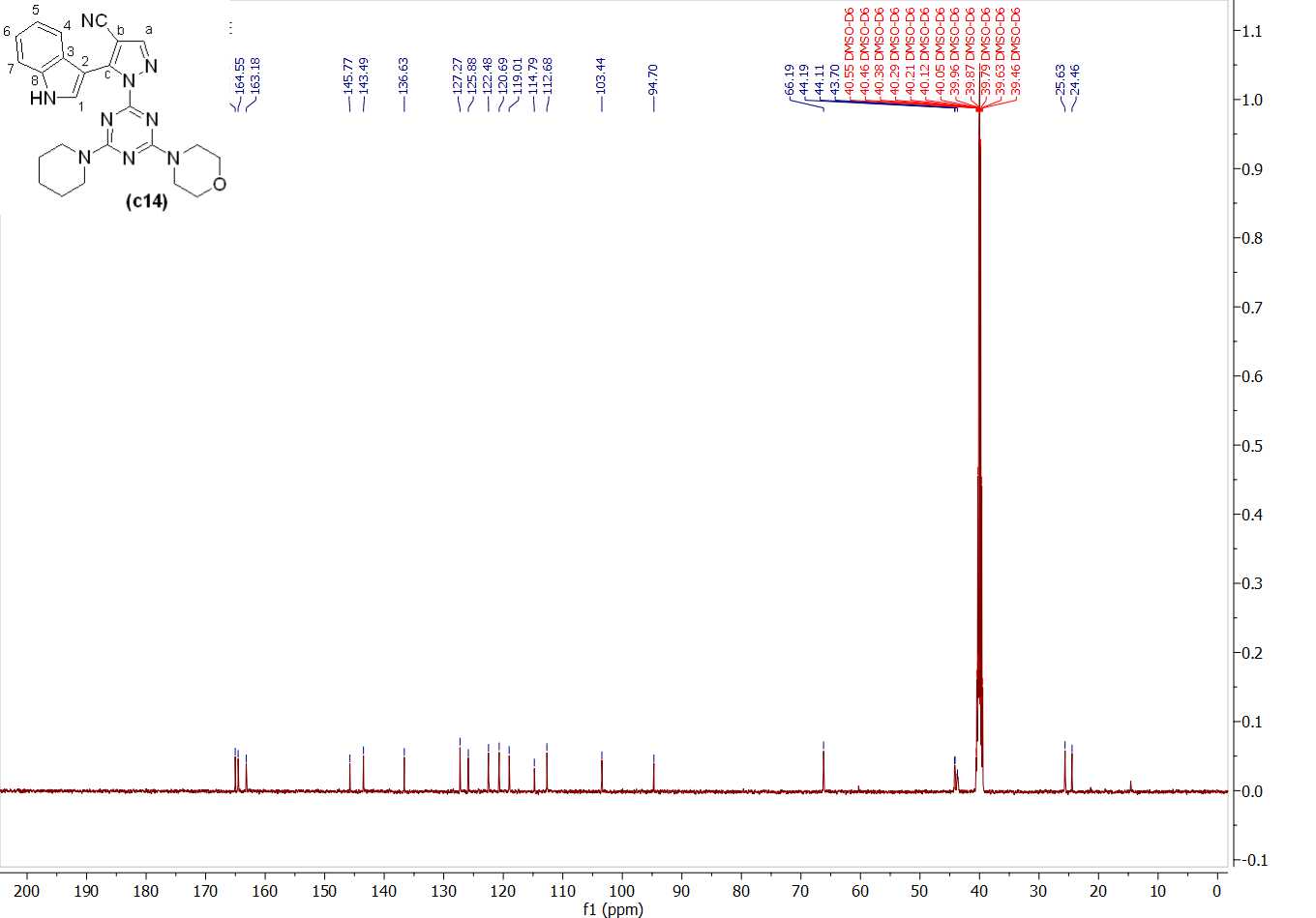


**Figure S14:** ^1^H-NMR and ^13^C-NMR of **5c**


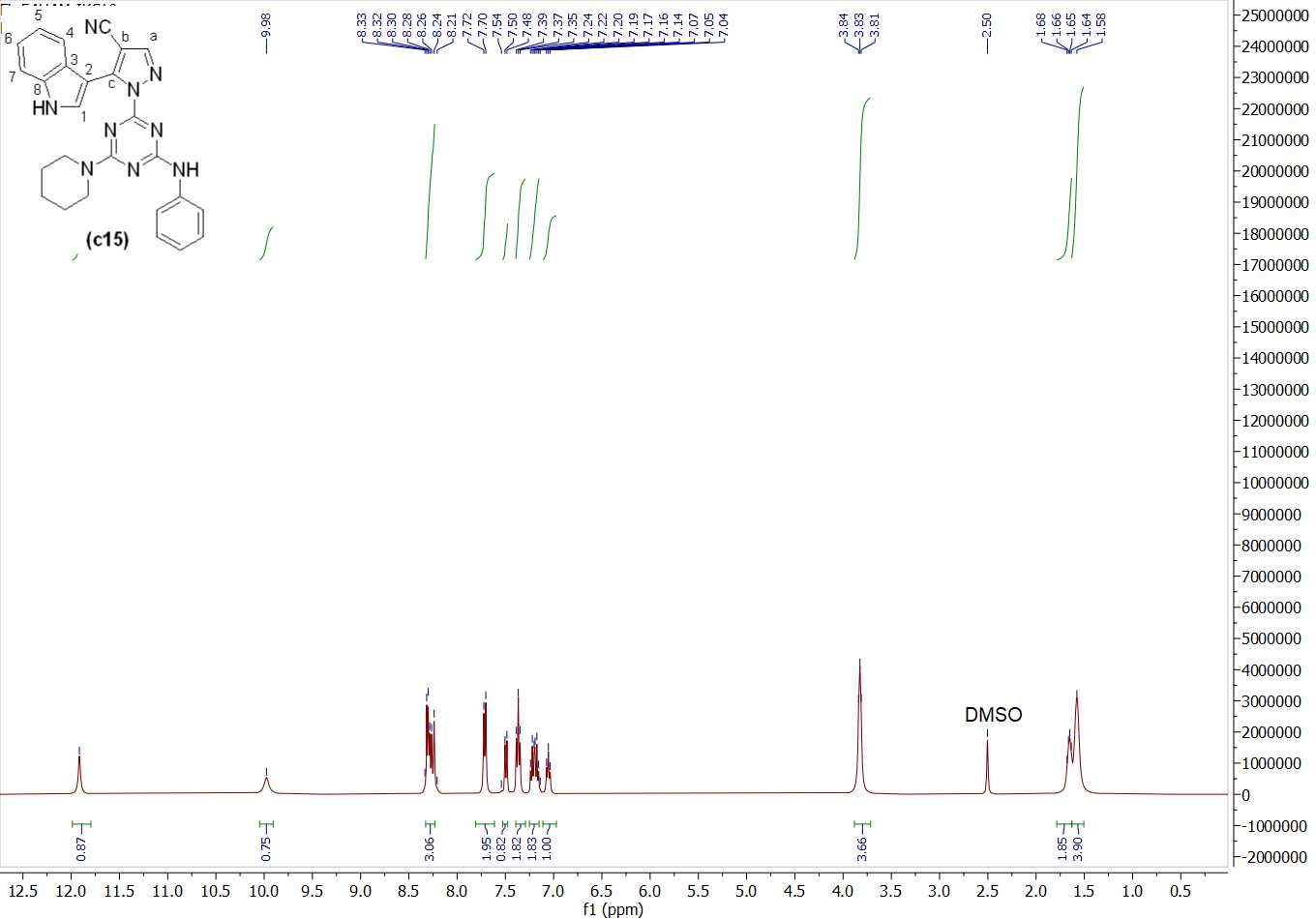


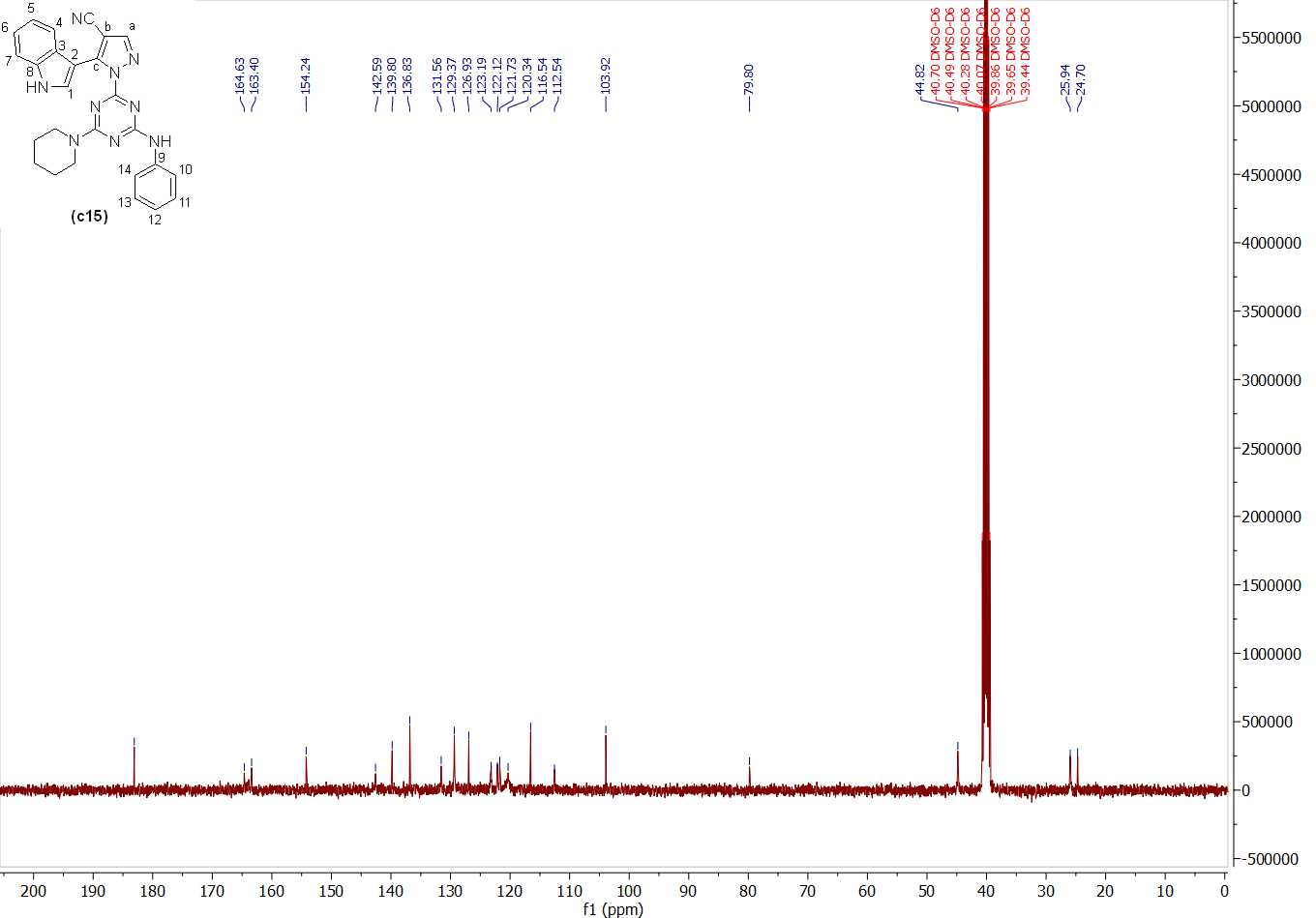


**Figure S15:** ^1^H-NMR and ^13^C-NMR of **5d**


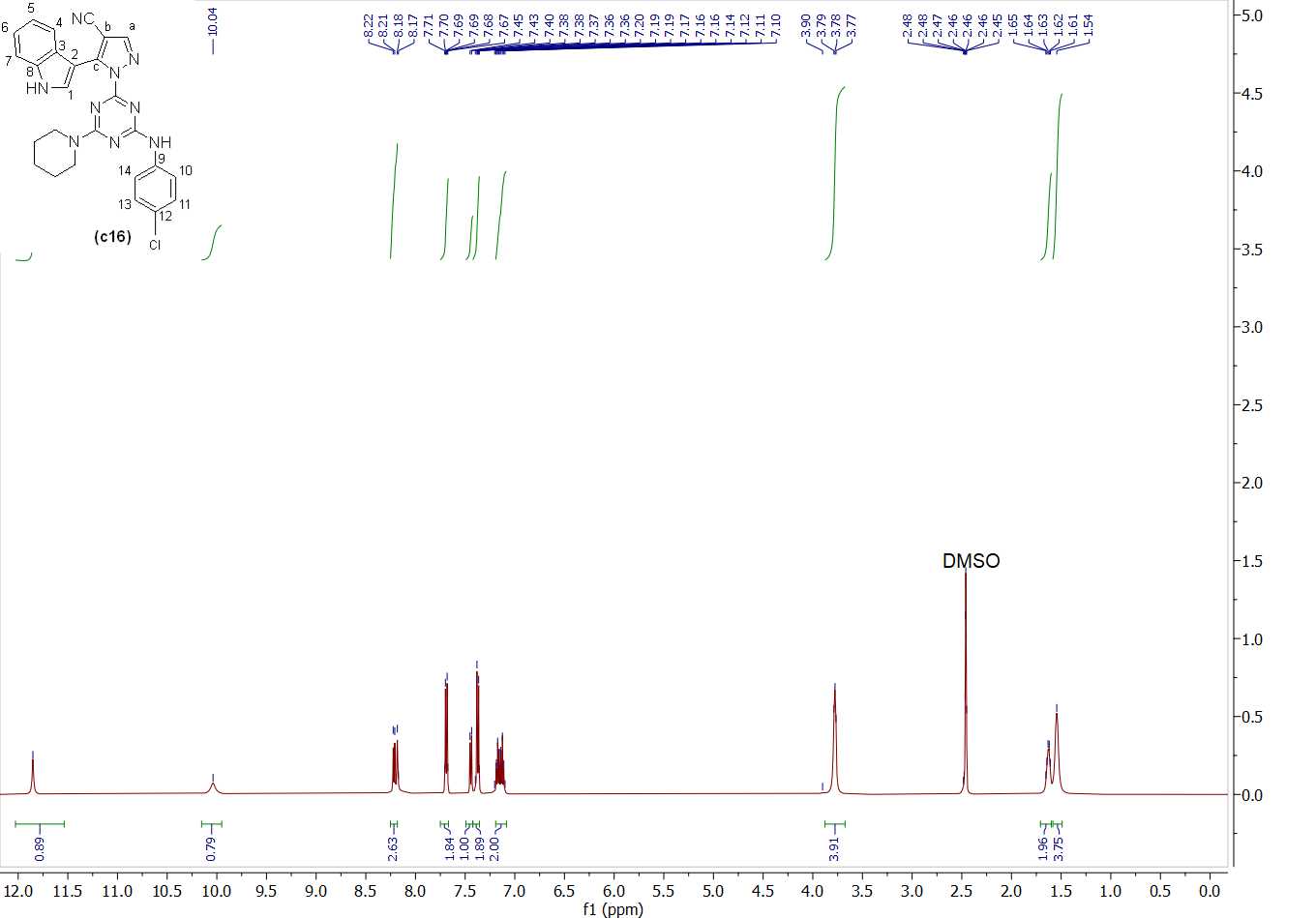


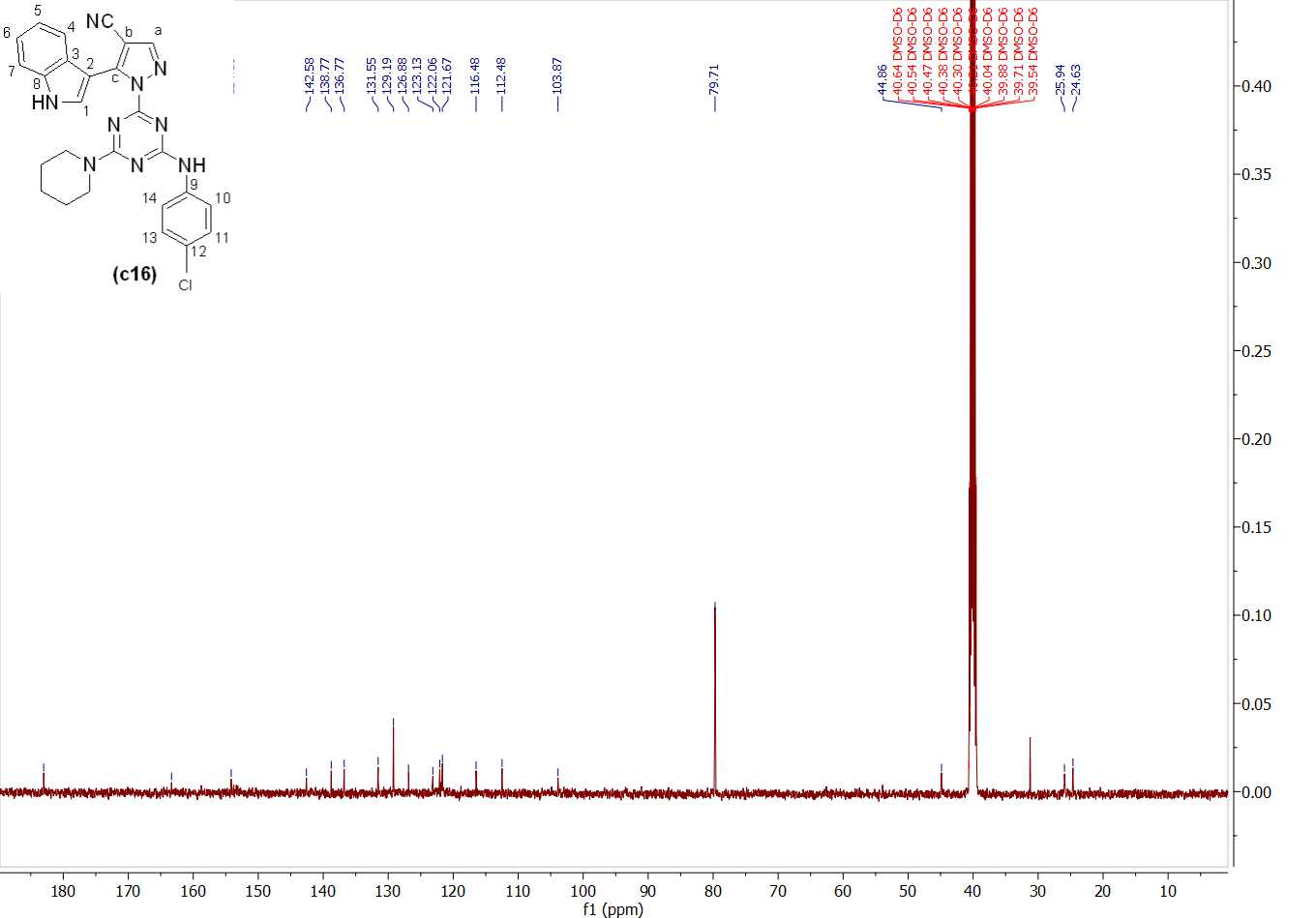


**Figure S16:** ^1^H-NMR and ^13^C-NMR of **5e**


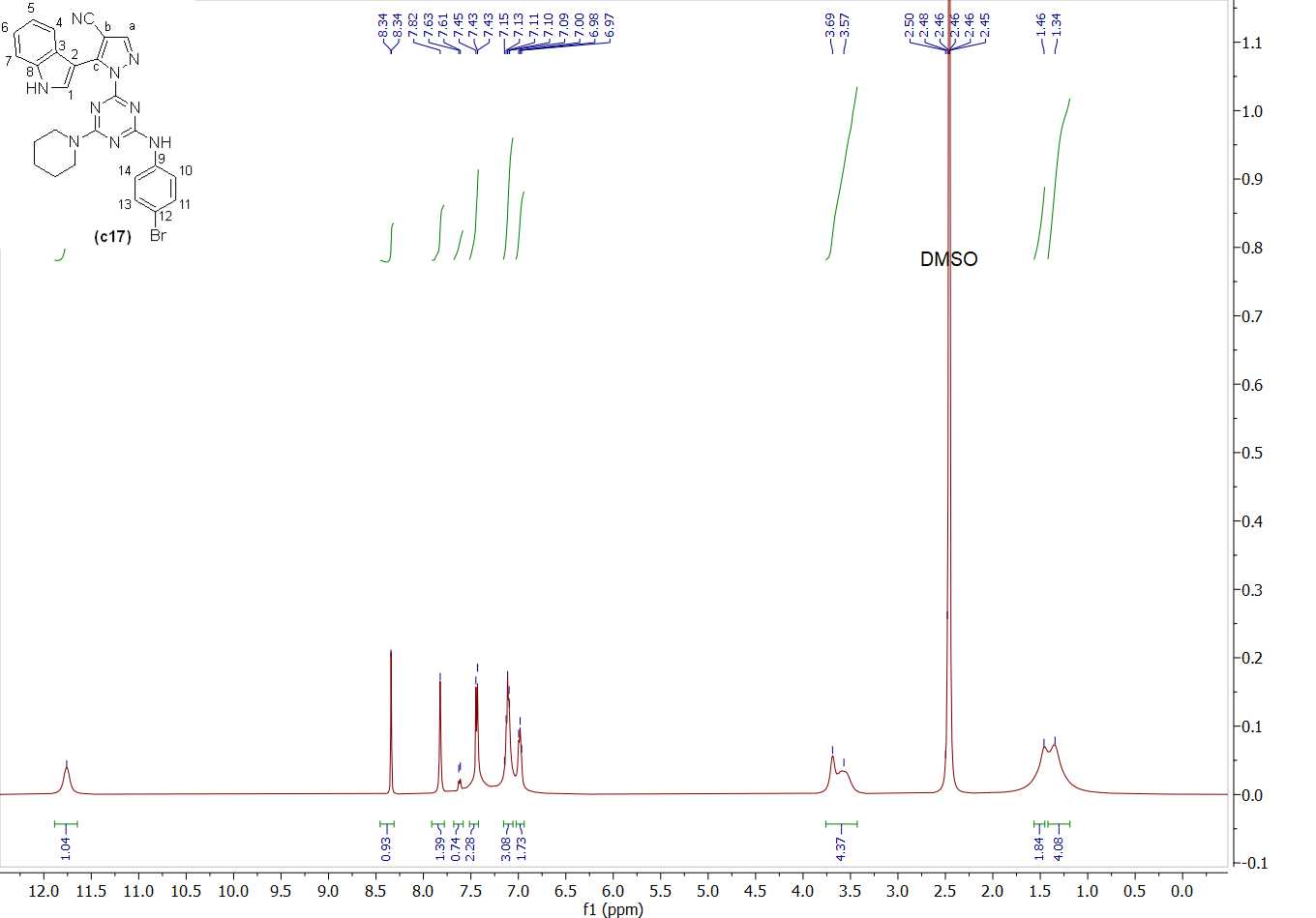


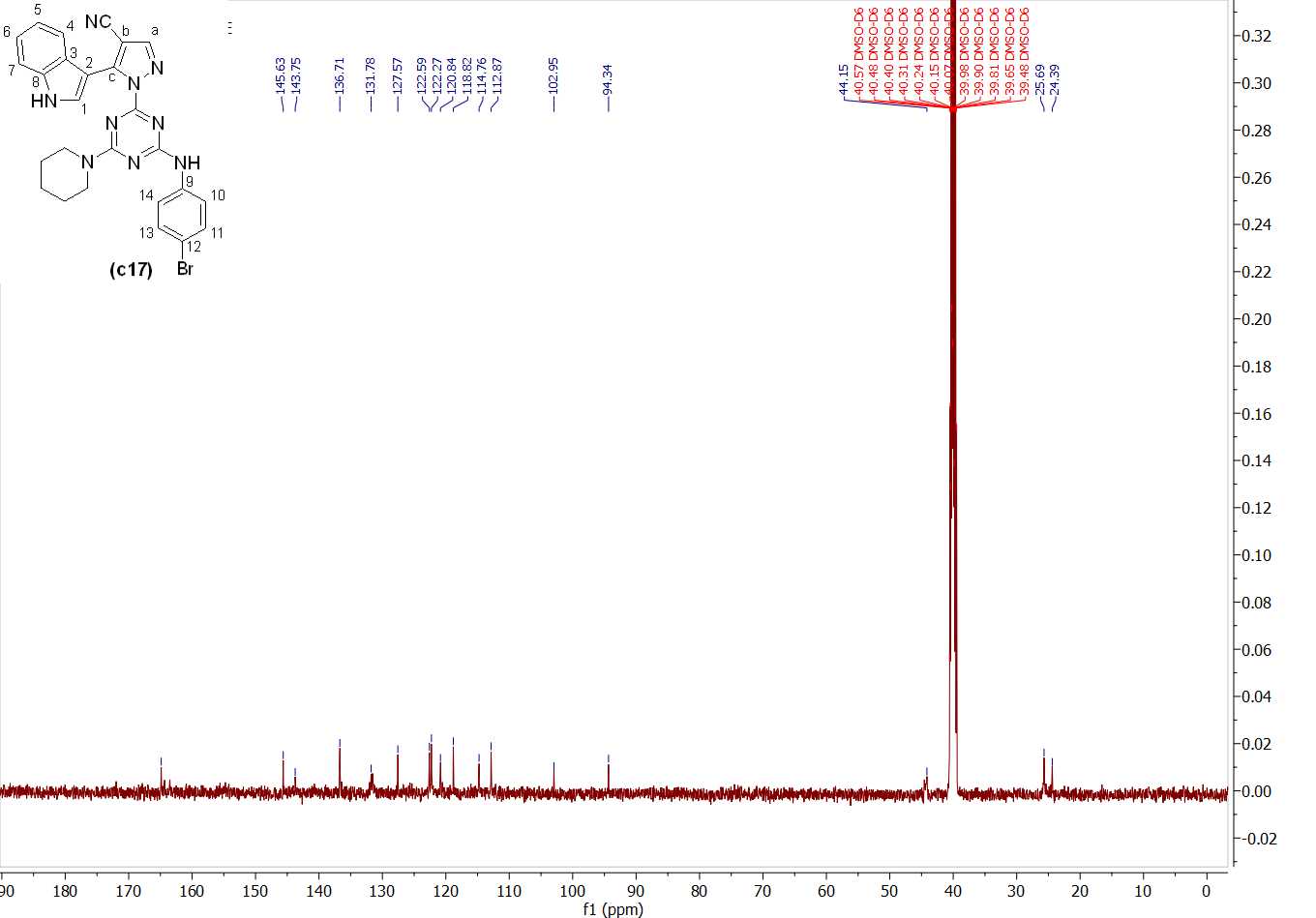


**Figure S17:** ^1^H-NMR and ^13^C-NMR of **5f**


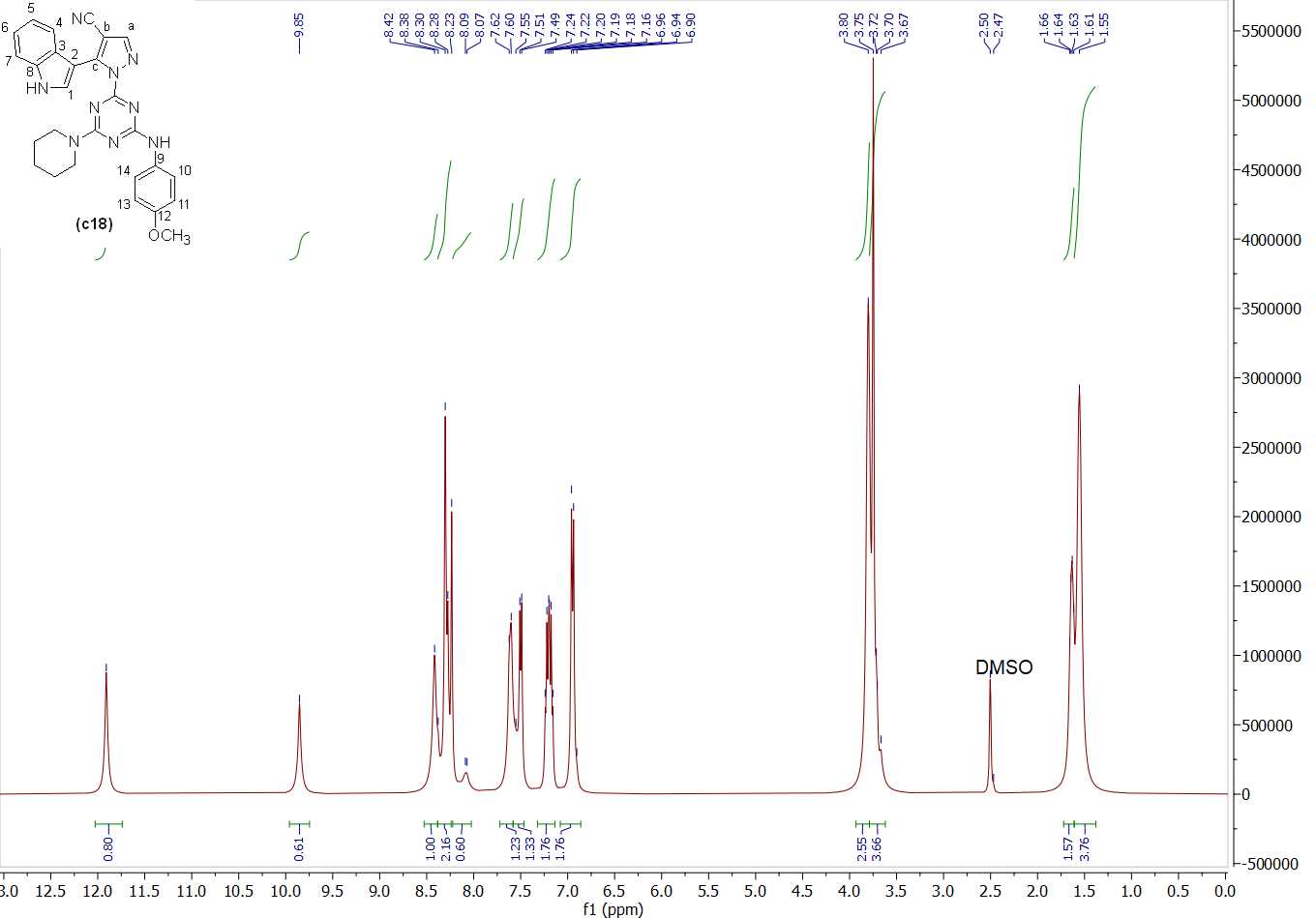


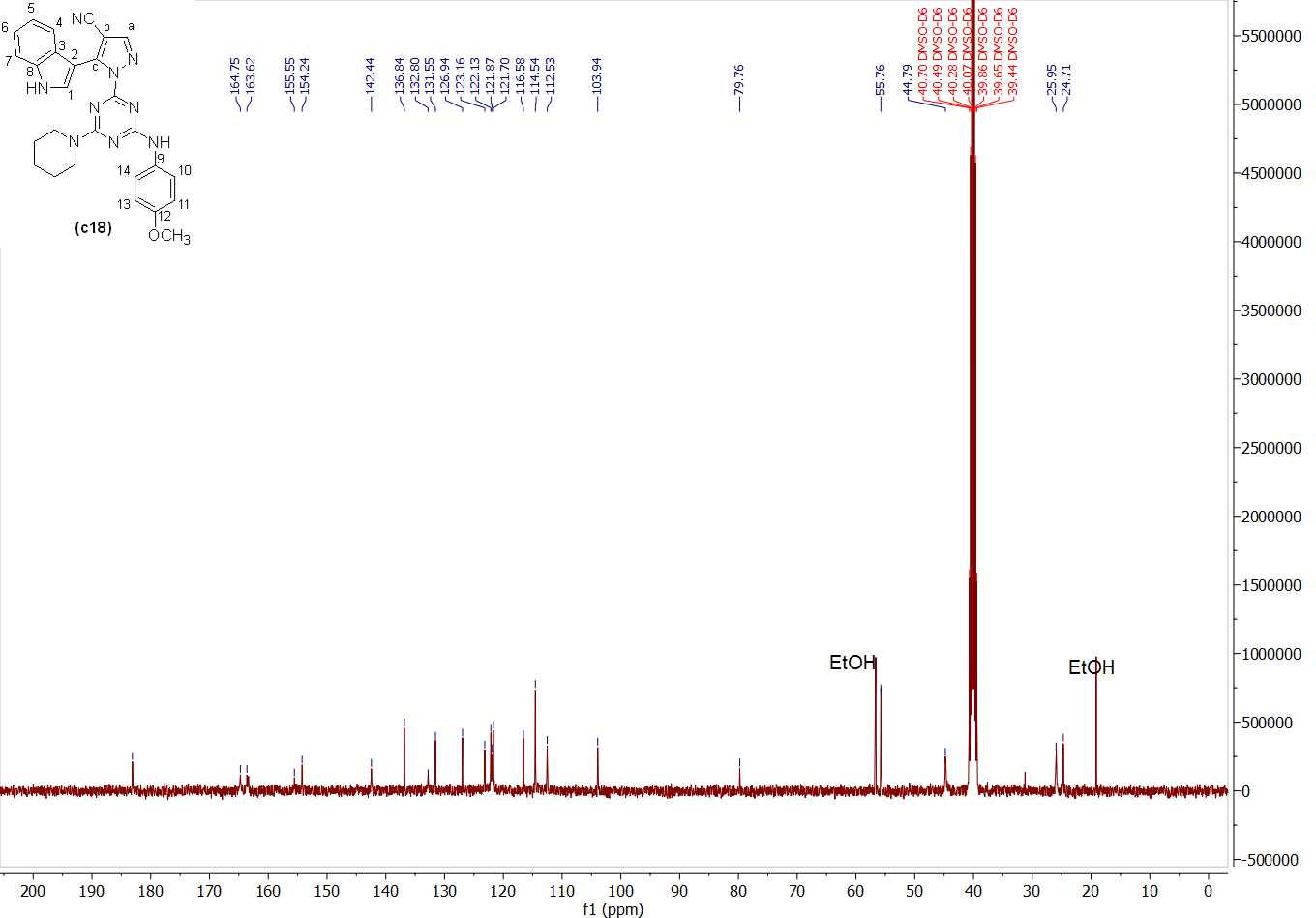


**Figure S18:** ^1^H-NMR and ^13^C-NMR of **5g**


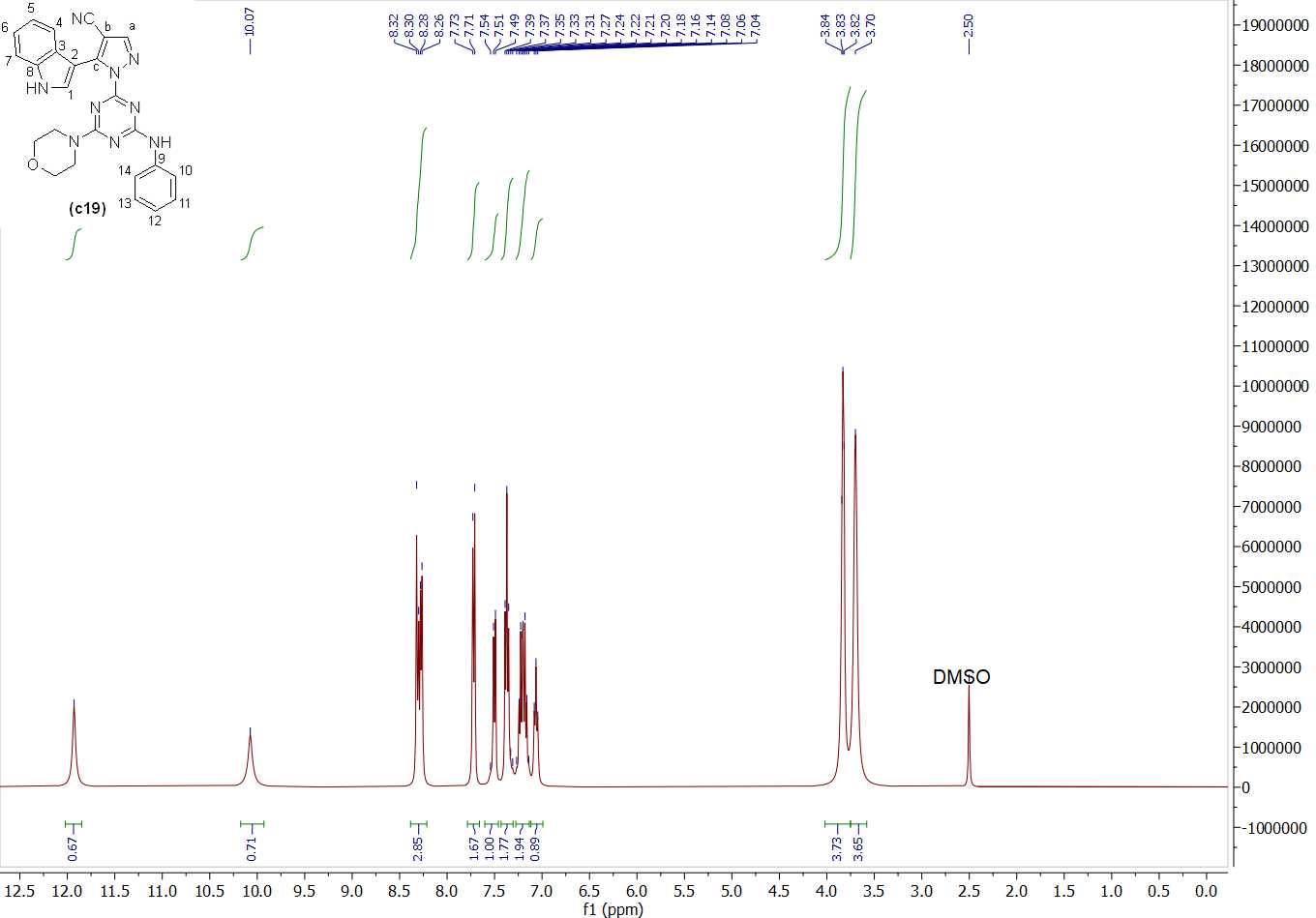


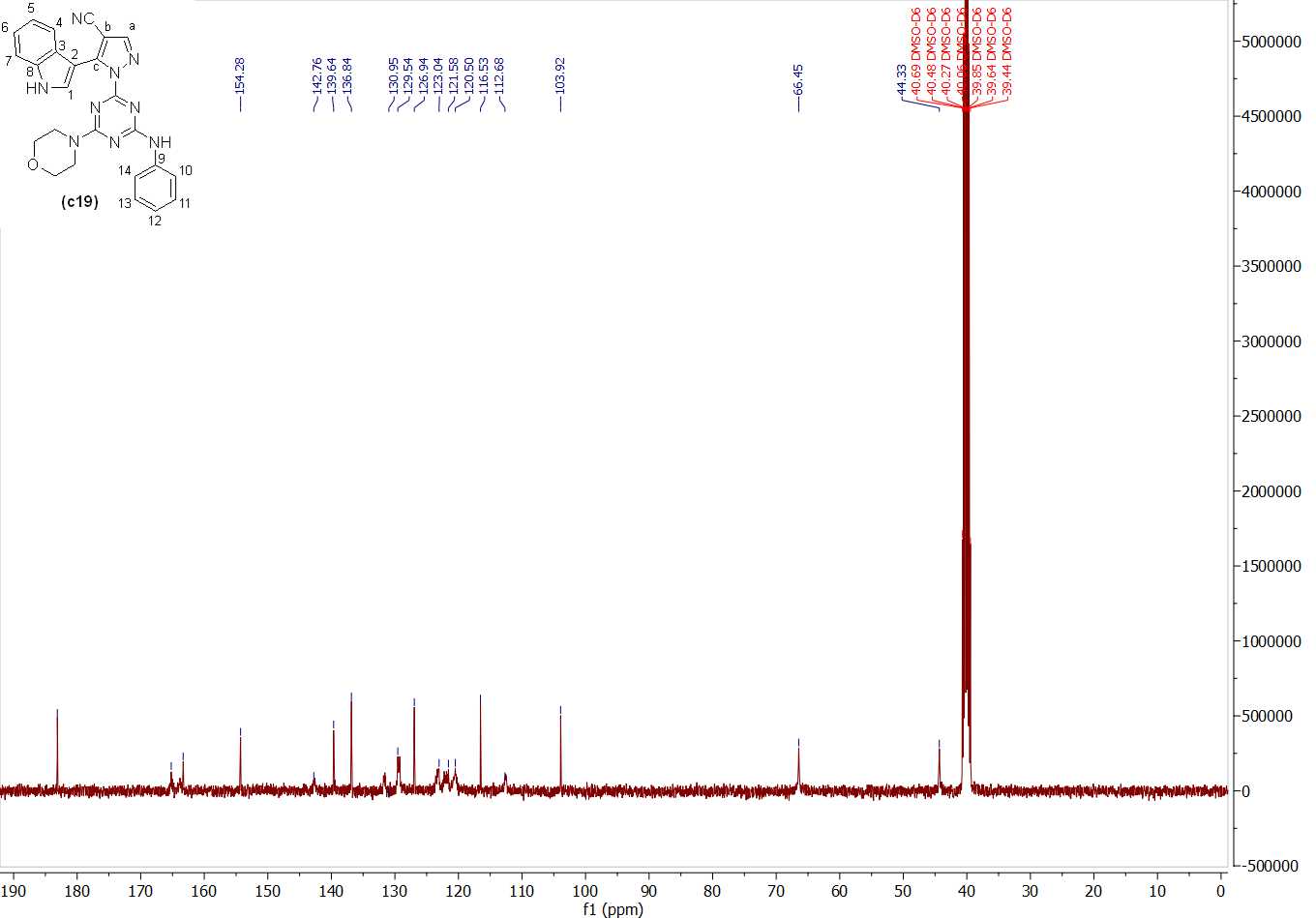


**Figure S19:** ^1^H-NMR and ^13^C-NMR of **5h**


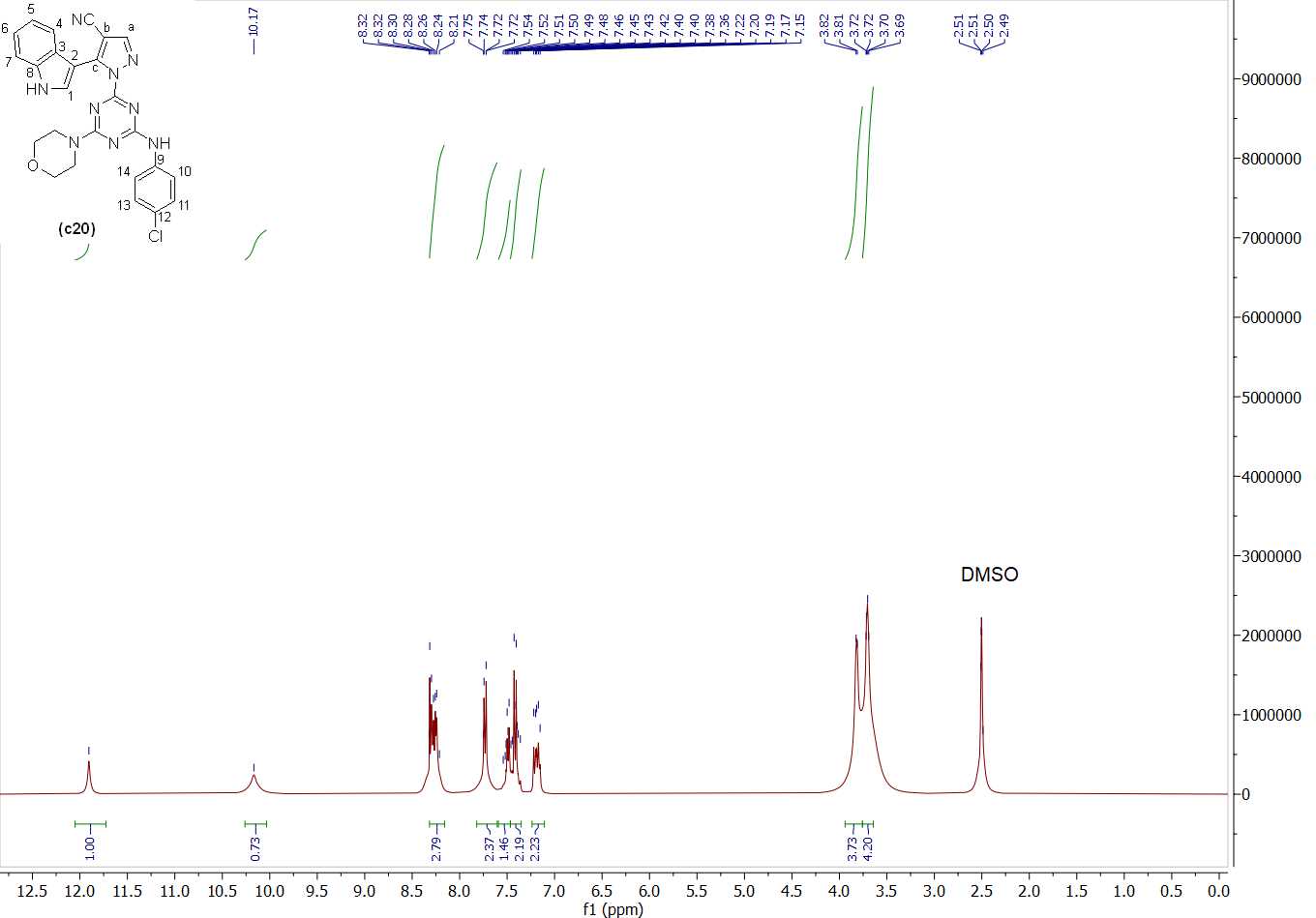


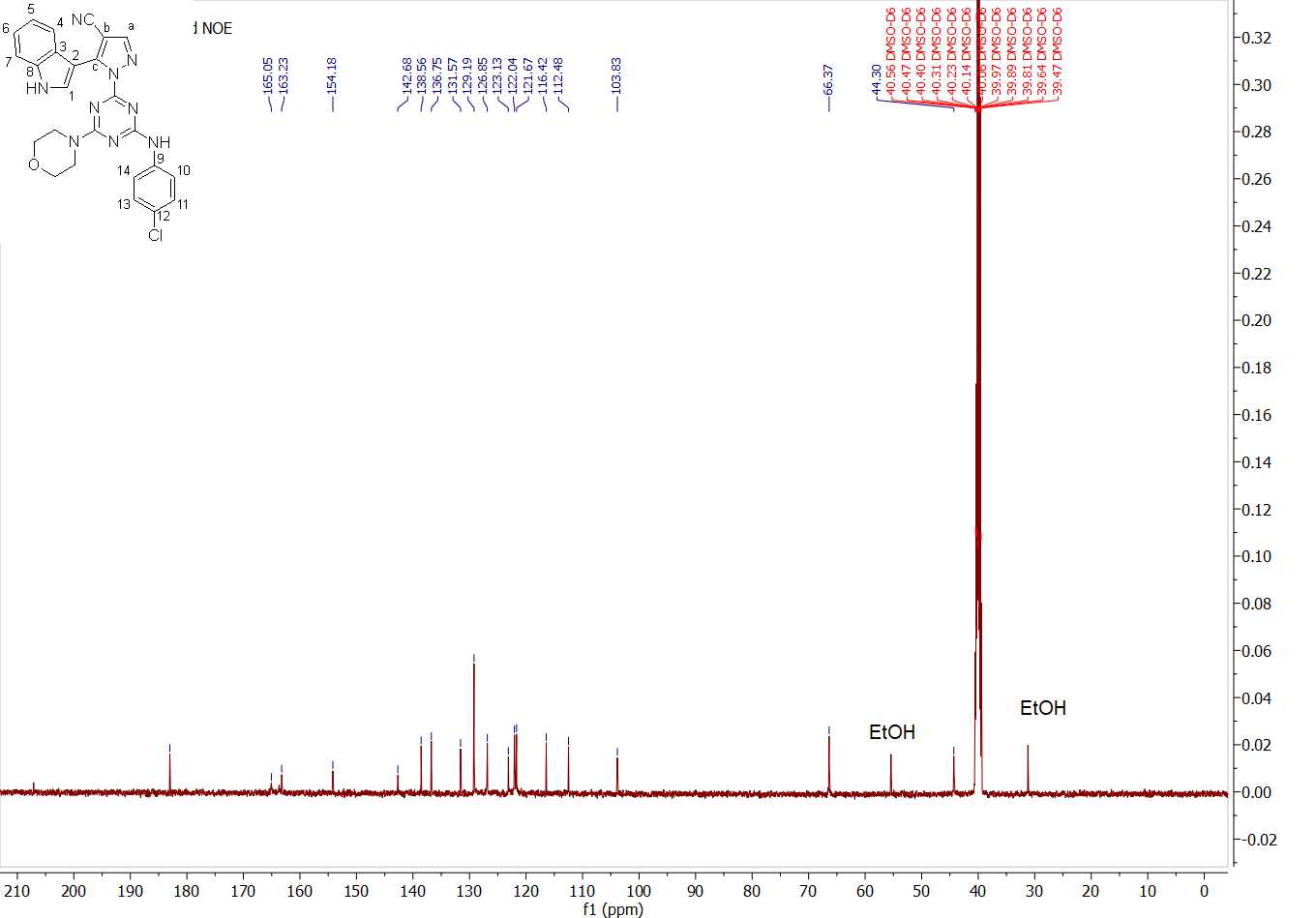


**Figure S20:** ^1^H-NMR and ^13^C-NMR of **5i**


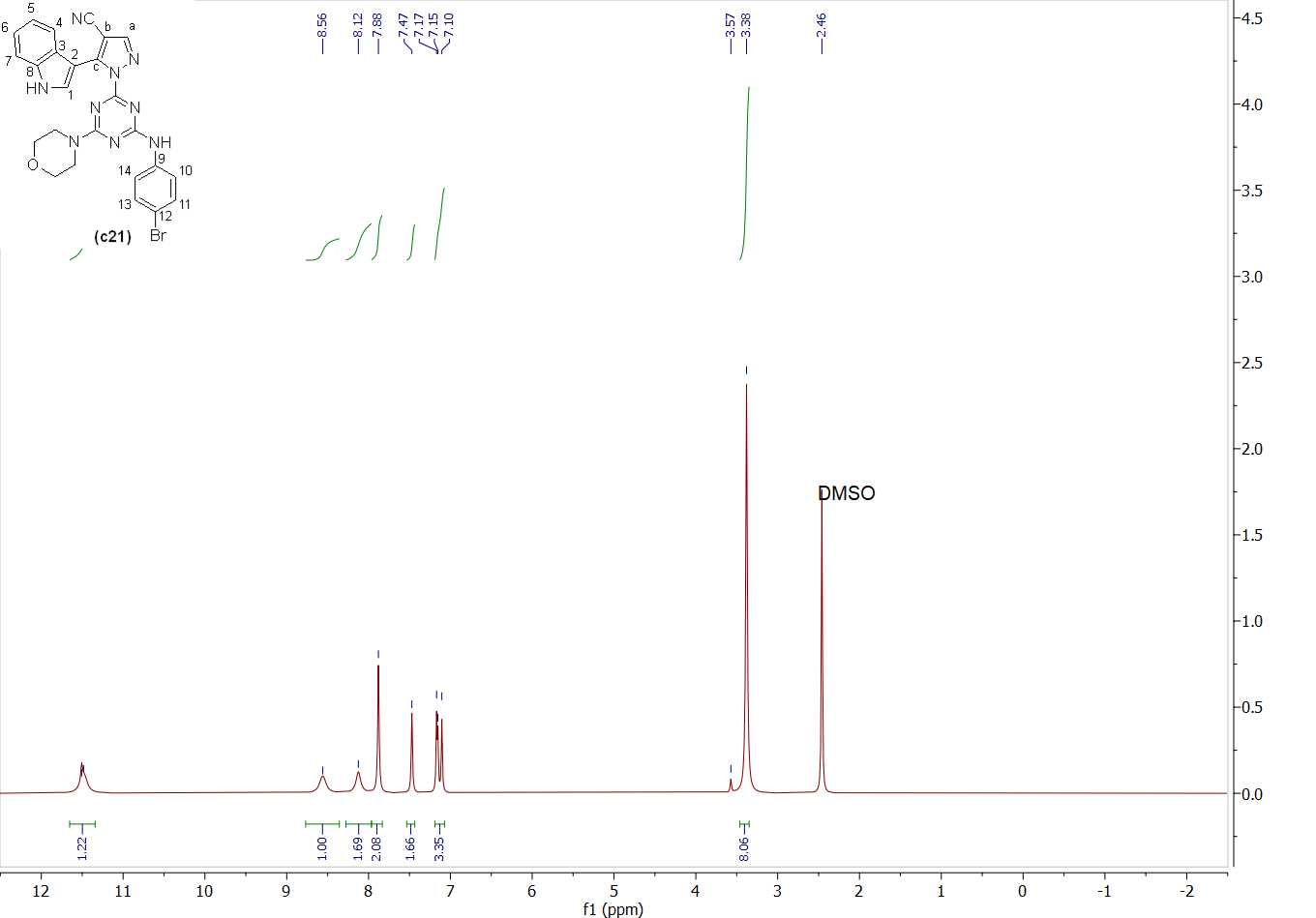


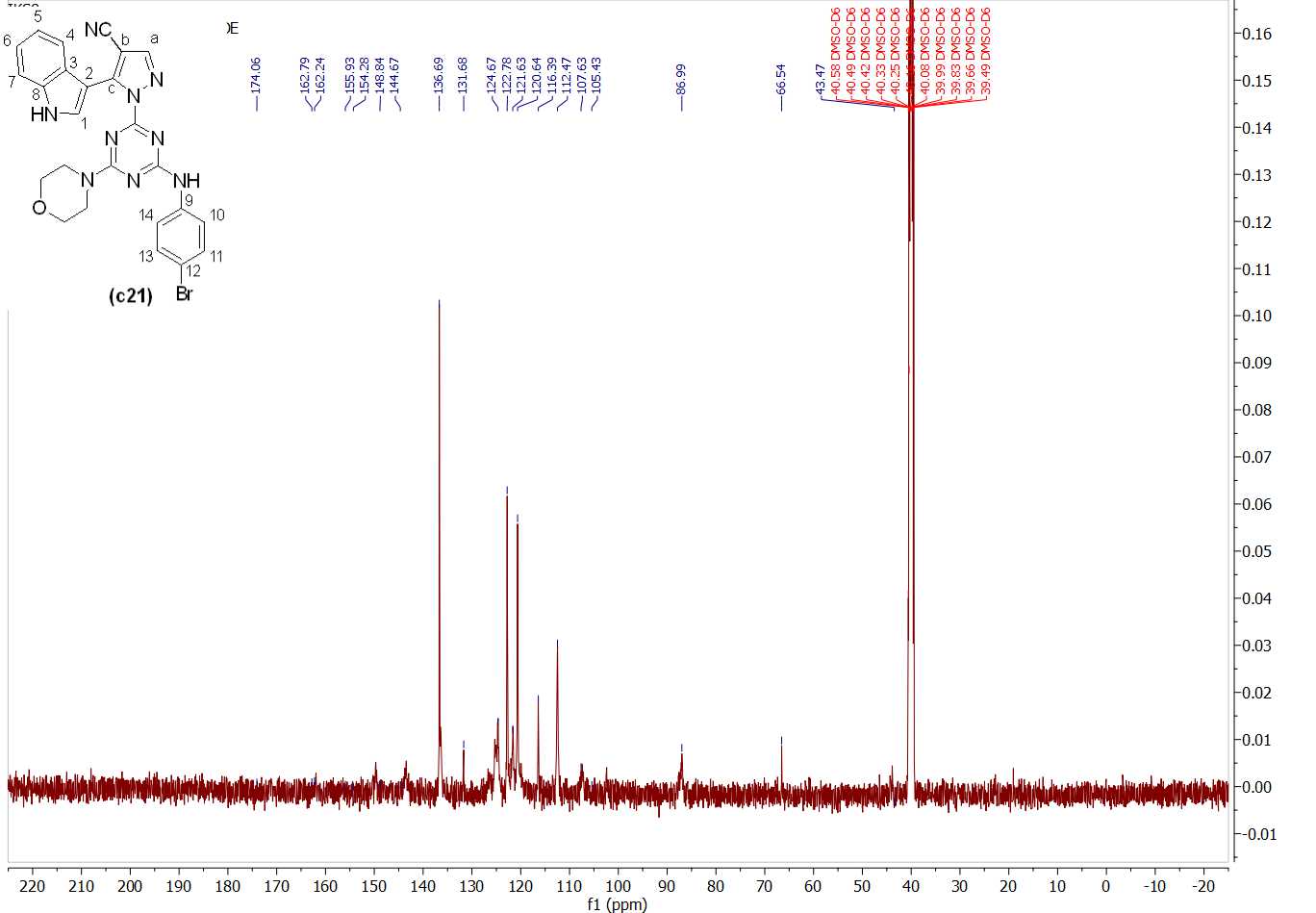


**Figure S21:** ^1^H-NMR and ^13^C-NMR of **5j**


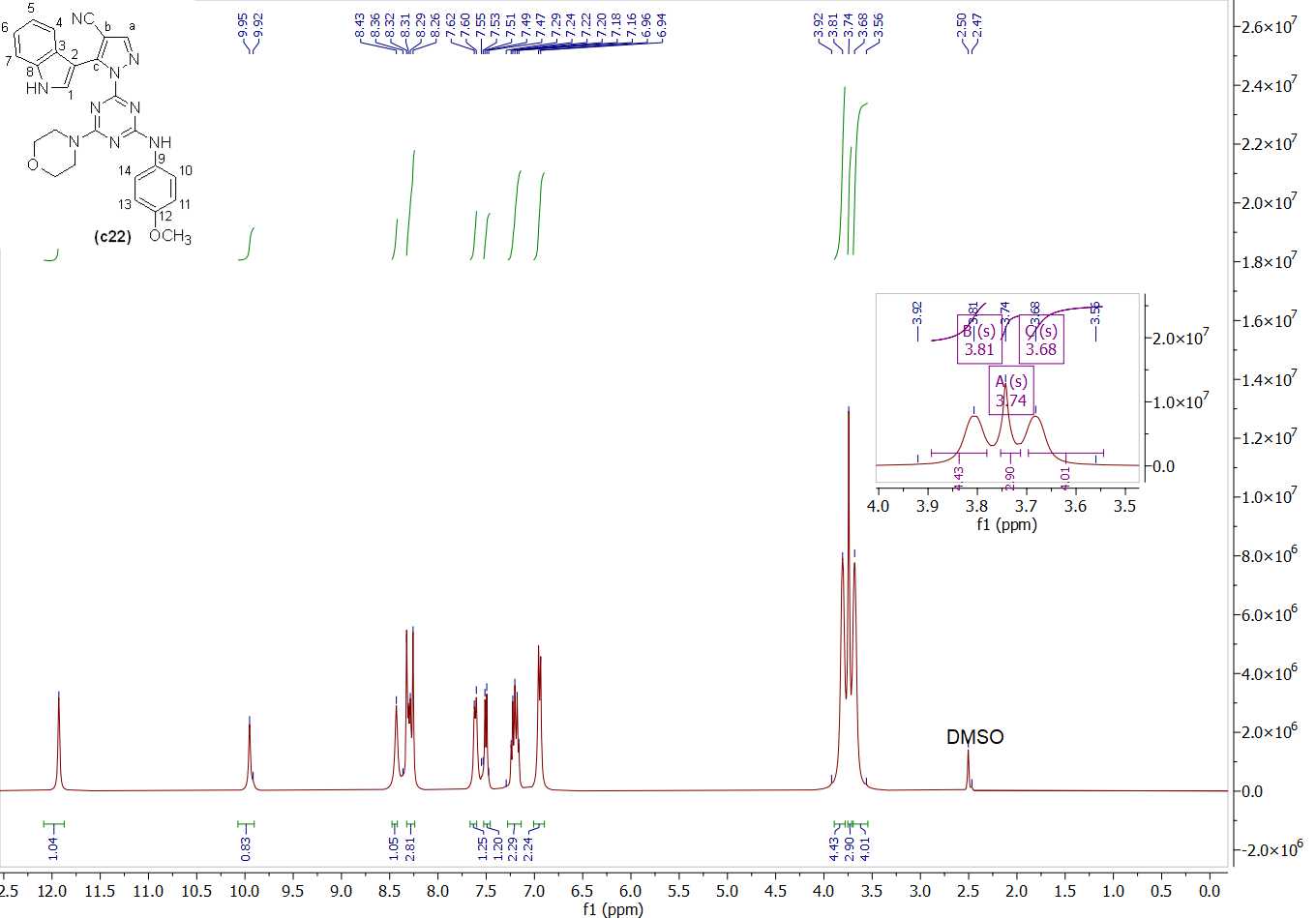


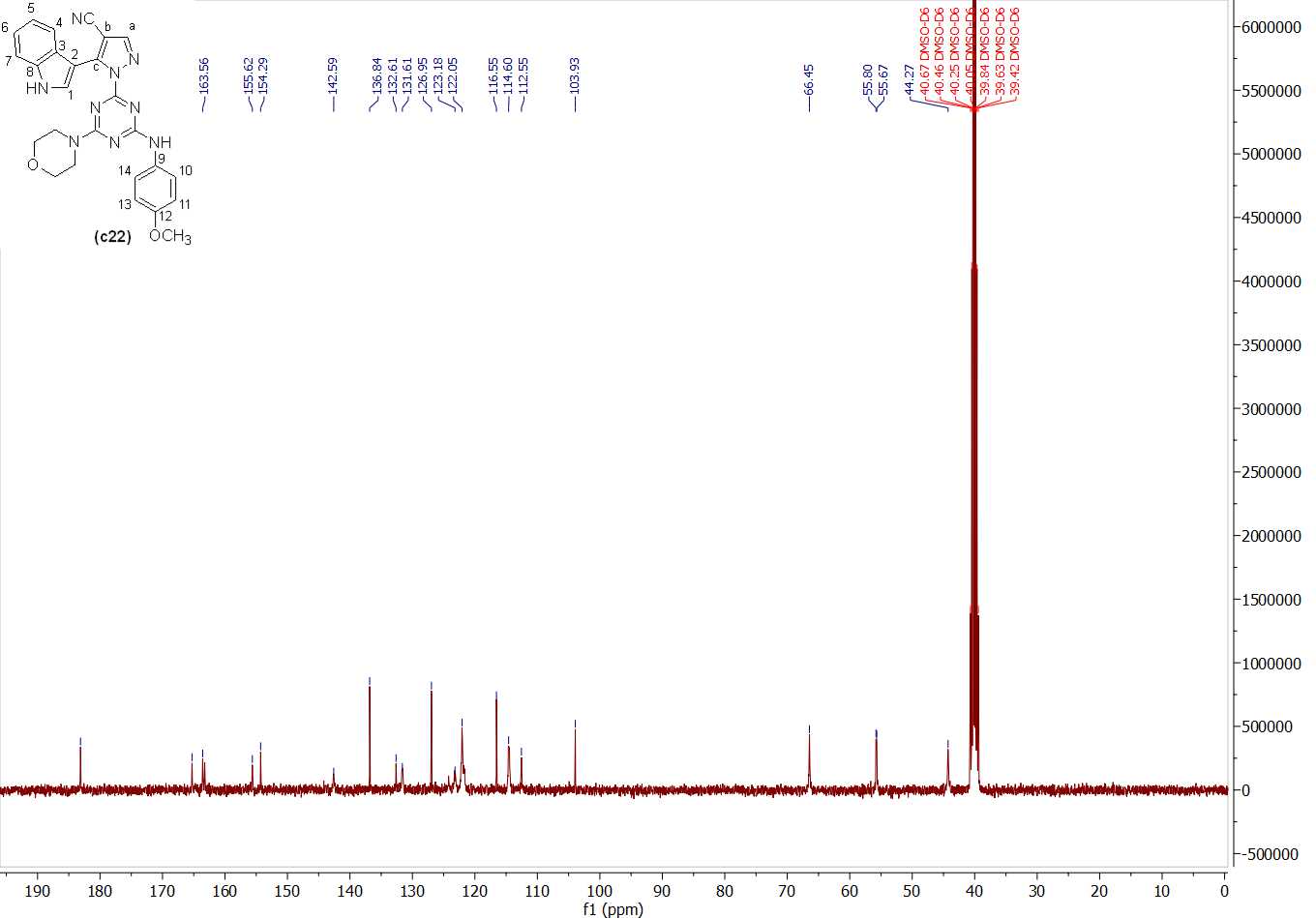


**Figure S22:** ^1^H-NMR and ^13^C-NMR of **5k**
