# Supplementary material for: Pyrazolyl-s-triazine with indole motif as a novel of epidermal growth factor receptor/cyclin-dependent kinase 2 dual inhibitors
Source: Front Chem. 2022 Nov 25;10:1078163. doi: 10.3389/fchem.2022.1078163 (PMC9732672; doi:10.3389/fchem.2022.1078163)
Supplement: Supplementary file 2 [file Image1.pdf]

Synthesis of **Pyrazolyl-s-Triazine-Indole** derivatives

Novel Pyrazolyl-s-Triazine-based derivatives exhibited potent cytotoxicity against lung A549 cell line

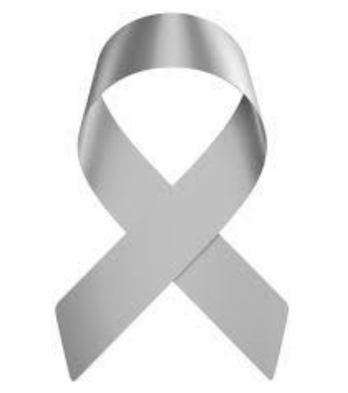

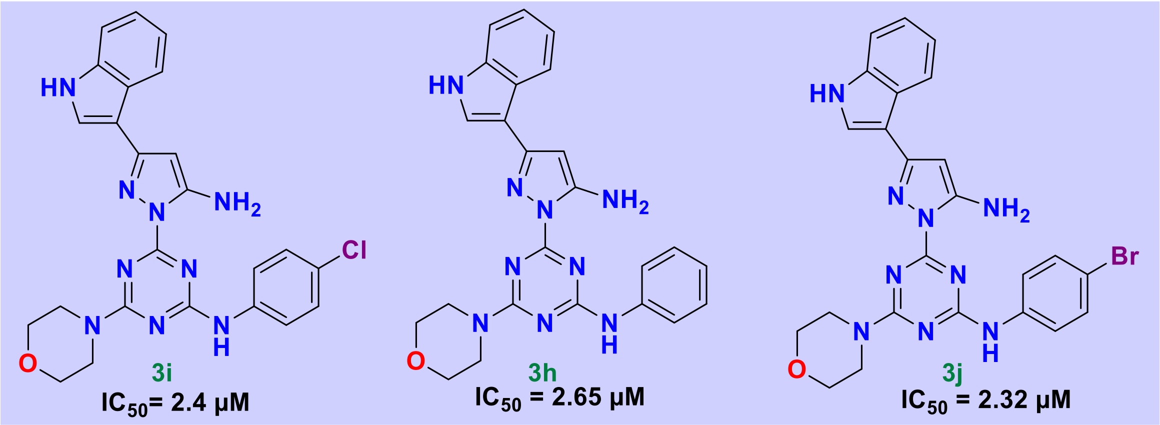

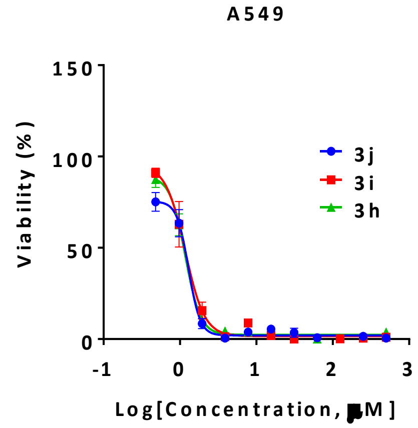

Apoptosis-inducing activity through **EGFR/CDK-2** inhibition

- **Apoptosis-inducing activity of 3j, 3i and 3h cells by 50.9, 71.6, and 51.4-folds in A549 cells with gene expression investigation**

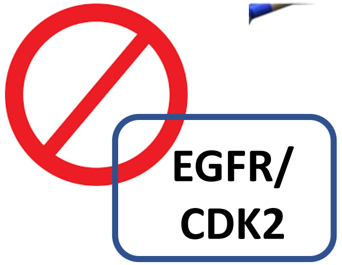

3i/ EGFR

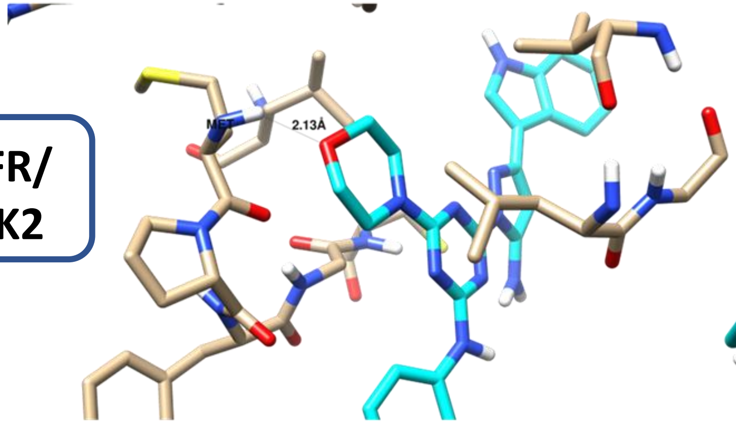

3i/ CDK-2

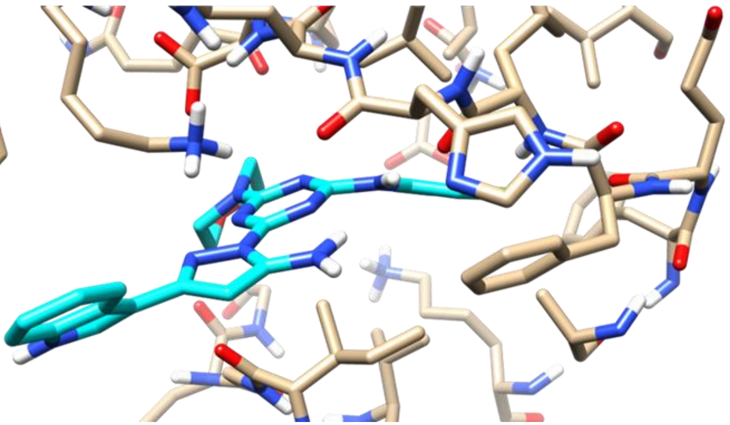

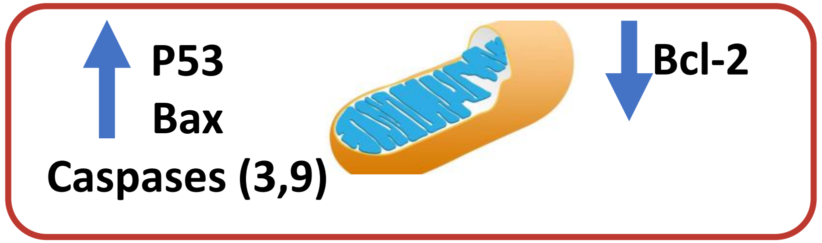

Apoptosis
